# Design, synthesis, and insecticidal activity evaluation of piperine derivatives

**DOI:** 10.3389/fchem.2022.973630

**Published:** 2022-07-26

**Authors:** Chiying Zhang, Qingqiang Tian, Yahui Li

**Affiliations:** ^1^ Key Laboratory of Agri-Food Safety of Anhui Province, School of Resources and Environment, Anhui Agricultural University, Hefei, China; ^2^ State Key Laboratory Breeding Base of Green Pesticide and Agricultural Bioengineering, Key Laboratory of Green Pesticide and Agricultural Bio-engineering, Ministry of Education, Guizhou University, Guiyang, China

**Keywords:** piperine derivatives, insecticidal activity, linear bisamide, *Plutella xylostella*, Lepidoptera

## Abstract

Structural optimization of natural products has become one of the most effective ways to develop novel pesticides. In this study, 30 novel pesticide derivatives containing a linear bisamide were synthesized. Then, their insecticidal activities against *P. xylostella* were evaluated. Results indicate that different bisamide substitutes show different larvicidal structure–activity relationships. At the same time, 2-trifluoroethyl is the most efficient substituent. The bioactivity results showed that most of the desired compounds exhibited better insecticidal activity against *P. xylostella* than piperine. Among them, **compound D28** resulted in 90% mortality at 1 mg/ml concentration. This study provides a novel protocol for the discovery of new insecticides. The molecular docking results indicated that compound D28 could act on *γ*-aminobutyric acid receptors.

## 1 Introduction

*Plutella xylostella*, also known as “dangling silkworm” or “diamondback moth,” is a pest commonly found on vegetables, causing damage by larvae feeding on the leaves of cruciferous vegetables. In recent years, the damage of *Plutella xylostella* has become increasingly serious, with significant adverse effects on the yield and quality of cruciferous vegetables (Wang et al., 2011; Zhao et al., 2022). The primary commercial agents used to control *Plutella xylostella* are traditional insecticides such as emamectin. However, long-term use of these insecticides results in moderate to high resistance (Lima Neto et al., 2021; Shen et al., 2017). Consequently, developing novel insecticides is an important endeavor (He et al., 2016; Liu et al., 2020; Xia et al., 2022). During the past few decades, natural product structural optimization has been a promising way to develop high-efficiency pesticides (Swain, 1977; Sun et al., 2013). In agricultural pest prevention, natural products have unique advantages (Tang et al., 2012; Roman, 2016; Liu et al., 2018), such as 1) it is relatively safe for higher mammals and natural enemies of pests; 2) it is environmentally friendly (Tong et al., 2018); 3) it has a new insecticidal mode of action (Gaur and Bao, 2021); and 4) it reduces pesticide resistance (Chen et al., 2017). For example, Neemaceae showed broad-spectrum insecticidal activity against many plant pests. Euonymus has been successfully commercialized to control rice pests, stored grain pests, and tree pests (Wu et al., 2001; Tang et al., 2004).

Piperine, as a cinnamon amide alkaloid (Parmar et al., 1997), shows a broad range of bioactivities such as antiobesity (Sunila and Kuttan, 2004), antiparasitic (Ribeiro et al., 2004.; Franklim et al., 2013), and lipid-lowering effects (Kimura et al., 2006; Park et al., 2012). In addition, piperine and its derivatives exhibit effective insecticidal properties against various agricultural pests. For example, Barbosa et al. reported a series of piperine derivatives by modifying the piperidine ring of piperine, which showed effective insecticidal activity against *Ascia monuste orseis* (Paula et al., 2000). Ribero et al. (2004) designed a series of piperine derivatives with effective insecticidal activity against *Trypanosoma cruzi*. Xu and Yang, (2017) found that piperine derivatives show stomach toxicity activity against agricultural pests with effects comparable to the commercial botanical pesticide toosendanin (CN107892685A). Han et al. (2021) designed a series of compounds that combined the benzo[*d*][1,3]dioxole moiety of piperine, which showed effective insecticidal activity against *Ostrinia furnacalis*.

**FIGURE 1:**
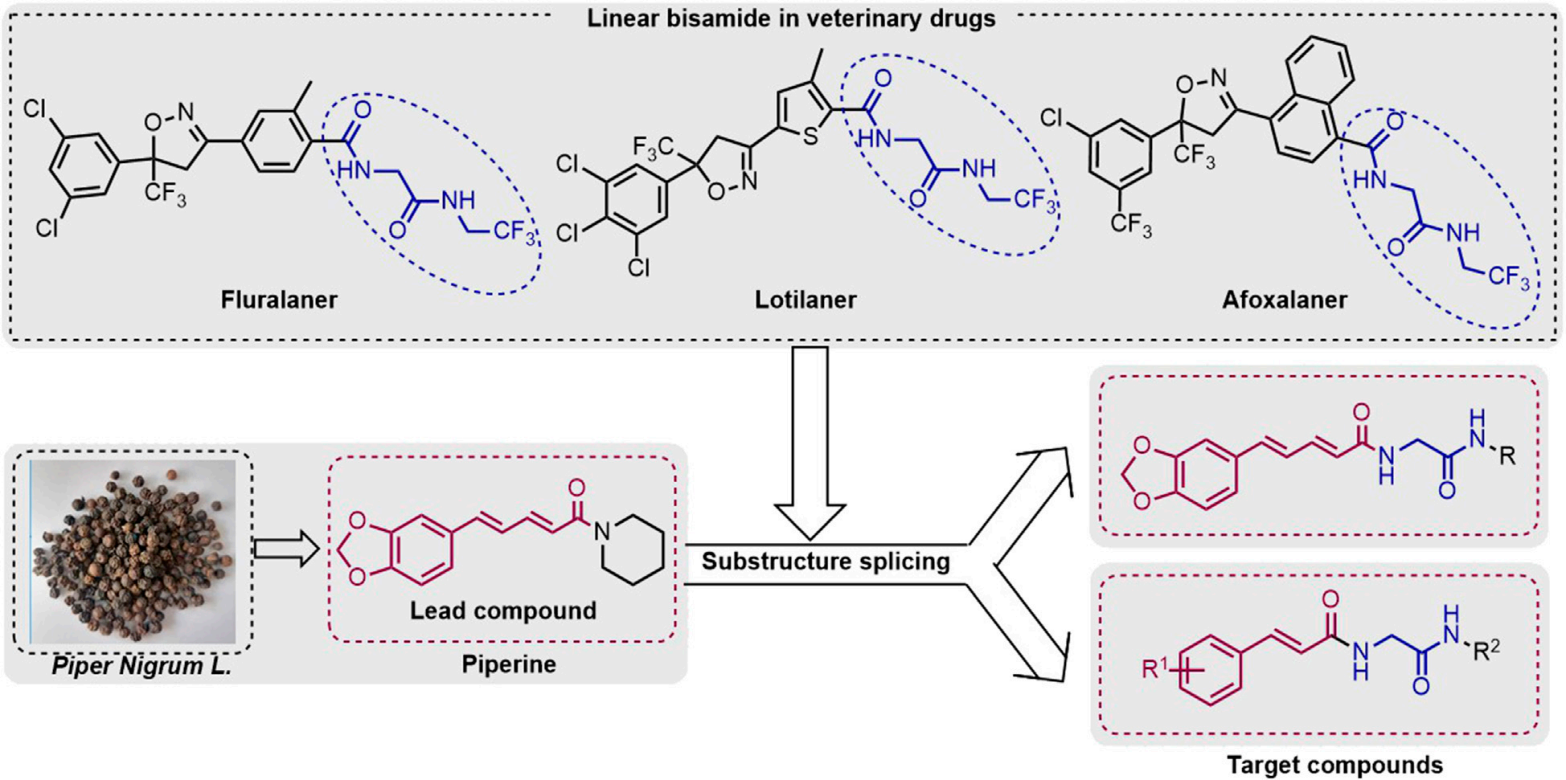
Overall design idea of this work.

Bisamide compounds show effective biological activities for agricultural pest control. However, the use of linear bisamides in the development of insecticides is still in its infancy. In contrast, linear bisamides are significant structural motifs in some veterinary drugs (for example, fluralaner, lotilaner, and afoxolaner) (Figure 1). Due to the novel action mechanism and viable activity of linear bisamides and the piperine skeleton, this study designed and synthesized a variety of piperine bisamide derivatives. The target compounds’ insecticidal activities against *P. xylostella* were systematically investigated.

## 2 Materials and methods

### 2.1 Chemical part

#### 2.1.1 General information

Most of the chemicals were purchased from Aladdin, energy-chemical, TCI, or Alfa Aesar. NMR spectra were recorded on Bruker Avance 600 spectrometers. Chemical shifts (ppm) were given relative to the solvent. Melting points of the target molecules were determined using a Shanghai Yice WRX-4 melting point meter. High-resolution mass spectrometry data were determined on a Thermo Scientific (UHPLC-Q-Orbitrap).

#### 2.1.2 Preparation of compound A

A solution of 20% KOH–EtOH (44 ml) was added to 5.7 g (20.0 mmol) of piperine at room temperature. The mixture was stirred at 80 C for 20 h. Then, 10% hydrochloric acid was added to adjust the pH of the mixture to 3. The mixture was filtered under reduced pressure, washed with water, and recrystallized in ethanol to obtain intermediate A in 81% yield (3.5 g).

***(2E,4E)-5-*** (***Benzo[d]*** [***1,3***] ***dioxol-5-yl***) ***penta-2,4-dienoic* (A)**: brown solid, 3.5g, yield 81%, mp: 163.4–166.5°C, ^1^H NMR (600 MHz, DMSO-*d*_*6*_) δ = 7.32–7.23 (m, 1H, Ar-H), 7.19 (s, 1H, Ar-H), 7.03–6.83 (m, 4H, -CH = CH-), 6.01 (s, 2H, -CH_2_), 5.90 (d, J = 15.1 Hz, 1H, Ar-H). ^13^C NMR (151 MHz, DMSO-*d*_*6*_) δ = 168.02, 148.52, 148.40, 144.97, 140.15, 130.96, 125.27, 123.42, 121.58, 108.91, 106.20, 101.77. HRMS (ESI) calcd for C_12_H_11_O_4_ [M + H]^+^: 219.06519, found 219.06514.

#### 2.1.3 General procedure for preparing compound C

A mixture of intermediate **A** (10.0 mmol, 2.18 g), glycine methyl ester hydrochloride (9.0 mmol, 1.13 g), HOBt (10.0 mmol, 1.35 g), DIEPA (10.0 mmol, 1.29 g), and 20 ml of DCM was stirred at 0°C for 15 min. Then, EDCI (10 mmol, 1.92 g) was added. The reaction was stirred at 25°C for 18 h. After the reaction was completed, the solvent was removed under reduced pressure, and the crude product was purified by column chromatography to obtain intermediate **B.** Then, a mixture of intermediate **B** (10.0 mmol, 2.88 g) and KOH (20.0 mmol, 1.12 g) was added in 30 ml of H_2_O/CH_3_OH/THF(*v:v:v* = 1:1:1) and stirred at room temperature for 12 h. Then, 10% of HCl solution was added until the solid no longer formed, and the crude product was purified by column chromatography to obtain intermediate **C**.

((***2E,4E***) ***-5-*** (***Benzo[d]*** [***1,3***] ***dioxol-5-yl***) ***penta-2,4-dienoyl***) ***glycine*** (***C***): brown solid, 2.56 g, yield 93%, mp: 214.5–216.7°C, ^1^H NMR (600 MHz, DMSO-*d*_*6*_) δ = 8.35 (s, 1H, -NH), 7.26–7.09 (m, 2H, Ar-H), 6.96 (d, *J* = 7.8 Hz, 1H, Ar-H), 6.92–6.65 (m, 3H, -CH = CH-), 6.13 (d, *J* = 15.0 Hz, 1H, -CH = CH-), 5.99 (s, 2H, -CH_2_), 3.82 (d, *J* = 5.5 Hz, 2H, -CH_2_). ^13^C NMR (151 MHz, DMSO-*d*_*6*_) δ = 171.72, 166.21, 148.33, 148.19, 140.49, 138.70, 131.18, 125.53, 124.22, 123.06, 108.88, 106.13, 101.67, 41.21. HRMS (ESI) calcd for C_14_H_14_O_5_N [M + H]^+^: 276.08665, found 276.08572.

#### 2.1.4 Preparation of target compounds D1–D28

A mixture of intermediate **C** (1.0 mmol, 275 mg), amine (1.2 mmol), HOBt (1.2 mol, 162.14 mg), DIEPA (1.2 mol, 155.1 mg), and 10 ml of DCM was stirred at 0°C for 15 min. Then, EDCI (1.2 mol, 230.0 mg) was added, and the reaction mixture was stirred at 25°C for 18 h. After the reaction was completed, the solvent was removed under reduced pressure to give the crude product, and the target compounds were obtained *via* recrystallization using ethyl acetate and petroleum ether as the solvent.

***(2E,4E)-5-*** (***Benzo[d]*** [***1,3***] ***dioxol-5-yl***) ***-N-*** (***2-oxo-2-*** (***Phenylamino***) ***ethyl***) ***penta-2,4-dienamide*** (**D1**): brown solid, 81.2 mg, yield 23.2%, mp: >250.0°C, ^1^H NMR (600 MHz, DMSO-*d*_*6*_) δ = 9.99 (s, 1H, NH), 8.39 (t, *J* = 5.8 Hz, 1H, -NH), 7.57 (d, *J* = 7.8 Hz, 2H, Ar-H), 7.28 (t, *J* = 7.9 Hz, 2H, Ar-H), 7.24 (d, *J* = 1.1 Hz, 1H, Ar-H), 7.19–7.15 (m, 1H, Ar-H), 7.02 (t, *J* = 7.4 Hz, 1H, Ar-H), 6.98–6.83 (m, 4H, Ar-H and -CH = CH-), 6.19 (d, *J* = 15.0 Hz, 1H, -CH = CH-), 6.02 (s, 2H, -CH_2_), 3.96 (d, *J* = 5.9 Hz, 2H, -CH_2_). ^13^C NMR (151 MHz, DMSO-*d*_*6*_) δ = 168.24, 166.12, 148.37, 148.19, 140.24, 139.33, 138.57, 131.28, 129.14, 125.66, 124.56, 123.66, 123.06, 119.61, 108.86, 106.15, 101.68, 43.33. HRMS (ESI) calcd for C_20_H_19_O_4_N_2_ [M + H]^+^: 351.13393, found 351.13257.

***(2E,4E)-5-*** (***Benzo[d]*** [***1,3***] ***dioxol-5-yl***) ***-N-*** (***2-oxo-2-*** (***o-tolylamino***) ***ethyl***) ***penta-2,4-dienamide*** (**D2**): red solid, 88.0 mg, yield 24.1%, mp: >250.0°C, ^1^H NMR (600 MHz, DMSO-*d*_*6*_) δ = 9.32 (s, 1H, -NH), 8.43 (s, 1H, -NH), 7.40 (d, *J* = 7.4 Hz, 1H, Ar-H), 7.25–7.11 (m, 4H, Ar-H), 7.05 (d, *J* = 6.9 Hz, 1H, Ar-H), 6.98–6.84 (m, 4H, Ar-H and -CH = CH-), 6.19 (d, *J* = 15.0 Hz, 1H, -CH = CH-), 6.01 (s, 2H, -CH_2_), 4.00 (d, *J* = 4.5 Hz, 2H, -CH_2_), 2.17 (s, 3H, -CH_3_). ^13^C NMR (151 MHz, DMSO-*d*_*6*_) δ = 168.32, 166.25, 148.37, 148.20, 140.32, 138.63, 136.51, 131.83, 131.26, 130.71, 126.39, 125.64, 125.53, 125.00, 124.50, 123.07, 108.86, 106.15, 101.68, 43.24, 18.13. HRMS (ESI) calcd for C_21_H_21_O_4_N_2_ [M + H]^+^: 365.14958, found 365.14801.

***(2E,4E)-5-*** (***Benzo[d]*** [***1,3***] ***dioxol-5-yl***) ***-N-*** (***2-oxo-2-*** (***m-tolylamino***) ***ethyl***) ***penta-2,4-dienamide*** (**D3**): red solid, 148.1 mg, yield 40.6%, mp: 225.6–226.1°C, ^1^H NMR (600 MHz, DMSO-*d*_*6*_) δ = 9.90 (s, 1H, -NH), 8.37 (s, 1H, -NH), 7.42–7.32 (m, 2H, Ar-H), 7.24 (s, 1H, Ar-H), 7.18–7.14 (m, 2H, Ar-H), 6.97 (d, *J* = 7.9 Hz, 1H, Ar-H), 6.95–6.82 (m, 4H, Ar-H and -CH = CH-), 6.18 (d, *J* = 15.0 Hz, 1H, -CH = CH-), 6.01 (s, 2H, -CH_2_), 3.94 (d, *J* = 5.1 Hz, 2H, -CH_2_), 2.24 (s, 3H, -CH_3_). ^13^C NMR (151 MHz, DMSO-*d*_*6*_) δ = 168.17, 166.13, 148.36, 148.19, 140.26, 139.23, 138.60, 138.32, 131.26, 128.99, 125.64, 124.53, 124.38, 123.09, 120.13, 116.80, 108.87, 106.12, 101.68, 43.32, 21.60. HRMS (ESI) calcd for C_21_H_21_O_4_N_2_ [M + H]^+^: 365.14958, found 365.14841.

***(2E,4E)-5-*** (***Benzo[d]*** [***1,3***] ***dioxol-5-yl***) ***-N-*** (***2-oxo-2-*** (***p-tolylamino***) ***ethyl***) ***penta-2,4-dienamide*** (**D4**): red solid, 116 mg, yield 31.5%, mp: 178.8–180.7°C, ^1^H NMR (600 MHz, DMSO-*d*_*6*_) δ = 9.90 (s, 1H, -NH), 8.37 (t, *J* = 5.5 Hz, 1H, -NH), 7.44 (d, *J* = 8.2 Hz, 2H, Ar-H), 7.24 (s, 1H, Ar-H), 7.16 (m, 1H, Ar-H), 7.08 (d, *J* = 8.1 Hz, 2H, Ar-H), 6.99–6.81 (m, 4H, Ar-H and -CH = CH-), 6.18 (d, *J* = 15.0 Hz, 1H, -CH = CH-), 6.02 (s, 2H, -CH_2_), 3.94 (d, *J* = 5.7 Hz, 2H, -CH_2_), 2.22 (s, 3H, -CH_3_). ^13^C NMR (151 MHz, DMSO-*d*_*6*_) δ = 167.98, 166.09, 148.37, 148.19, 140.21, 138.55, 136.81, 132.58, 131.28, 129.51, 125.66, 124.59, 123.06, 119.64, 108.86, 106.15, 101.68, 43.28, 20.84. HRMS (ESI) calcd for C_21_H_21_O_4_N_2_ [M + H]^+^: 365.14958, found 365.14871.

***(2E,4E)-5-*** (***Benzo[d]*** [***1,3***] ***dioxol-5-yl***) ***-N-*** (***2-*** ((***4-*** (***tert-butyl***) ***phenyl***) ***amino***) ***-2-oxoethyl***) ***penta-2,4-dienamide*** (**D5**): red solid, 198.3 mg, yield 48.7%, mp: 124.4–125.1°C, ^1^H NMR (600 MHz, DMSO-*d*_*6*_) δ = 9.92 (s, 1H, NH), 8.38 (t, *J* = 5.3 Hz, 1H, -NH), 7.47 (d, *J* = 8.3, 2H, Ar-H), 7.29 (d, *J* = 8.4 Hz, 2H, Ar-H), 7.23 (s, 1H, Ar-H), 7.17 (m, 1H, Ar-H), 7.00–6.84 (m, 4H, Ar-H and -CH = CH-), 6.19 (d, *J* = 15.0 Hz, 1H, -CH = CH-), 6.01 (s, 2H, -CH = CH-), 3.95 (d, *J* = 5.6 Hz, 2H, -CH_2_), 1.22 (s, 9H, -CH_3_). ^13^C NMR (151 MHz, DMSO-*d*_*6*_) δ = 168.01, 166.13, 148.36, 148.19, 146.06, 140.24, 138.57, 136.71, 131.27, 125.72, 124.57, 123.05, 119.45, 114.17, 108.86, 106.15, 101.67, 43.24, 34.41, 31.62. HRMS (ESI) calcd for C_24_H_27_O_4_N_2_ [M + H]^+^: 407.19653, found 407.19501.

***(2E,4E)-5-*** (***Benzo[d]*** [***1,3***] ***dioxol-5-yl***) ***-N-*** (***2-*** ((***3,5-dimethylphenyl***) ***amino***) ***-2-oxoethyl***) ***penta-2,4-dienamide*** (**D6**): yellow solid, 73.4 mg, yield 19.4%, mp: >250.0°C ^1^H NMR (600 MHz, DMSO-*d*_*6*_) δ = 9.81 (s, 1H, -NH), 8.36 (t, *J* = 5.5 Hz, 1H, -NH), 7.24 (s, 1H, Ar-H), 7.21–7.12 (m, 3H, Ar-H), 6.98–6.83 (m, 4H, Ar-H and -CH = CH-), 6.66 (s, 1H, Ar-H), 6.18 (d, *J* = 15.0 Hz, 1H, -CH = CH-), 6.01 (s, 2H, -CH_2_), 3.92 (d, *J* = 5.7 Hz, 2H, -CH_2_), 2.20 (s, 6H, -CH_3_).^13^C NMR (151 MHz, DMSO-*d*_*6*_) δ = 168.08, 166.13, 148.37, 148.19, 140.22, 139.15, 138.57, 138.09, 131.27, 125.65, 125.22, 124.56, 123.06, 117.43, 108.86, 106.14, 101.68, 43.37, 21.51. HRMS (ESI) calcd for C_22_H_23_O_4_N_2_ [M + H]^+^: 379.16523, found 379.16409.

***(2E,4E)-5-*** (***Benzo[d]*** [***1,3***] ***dioxol-5-yl***) ***-N-*** (***2-*** ((***3-ethynylphenyl***) ***amino***) ***-2-oxoethyl***) ***penta-2,4-dienamide*** (**D7**): red solid, 59.8 mg, yield 15.9%, mp: 203.5–205.5°C, ^1^H NMR (600 MHz, DMSO-*d*_*6*_) δ = 10.10 (s, 1H, -NH), 8.40 (t, *J* = 5.5 Hz, 1H, -NH), 7.76 (s, 1H, Ar-H), 7.55 (d, *J* = 8.2 Hz, 1H, Ar-H), 7.30 (t, *J* = 7.9 Hz, 1H, Ar-H), 7.24 (s, 1H, Ar-H), 7.19–7.12 (m, 2H, Ar-H), 6.99–6.96 (m, 1H, Ar-H), 6.94–6.90 (m, 1H, -CH = CH-), 6.89–6.86 (m, 2H, -CH = CH-), 6.18 (d, *J* = 15.0 Hz, 1H, -CH = CH-), 6.02 (s, 2H, -CH_2_), 4.13 (s, 1H, CH≡), 3.96 (d, *J* = 5.8 Hz, 2H, -CH_2_). ^13^C NMR (151 MHz, DMSO-*d*_*6*_) δ = 168.58, 166.15, 148.37, 148.20, 140.28, 139.54, 138.61, 131.28, 129.63, 126.92, 125.65, 124.50, 123.08, 122.47, 122.44, 120.19, 108.86, 106.15, 101.68, 83.79, 80.90, 43.37. HRMS (ESI) calcd for C_22_H_19_O_4_N_2_ [M + H]^+^: 375.13393, found 375.13269.

***(2E,4E)-5-*** (***Benzo[d]*** [***1,3***] ***dioxol-5-yl***) ***-N-*** (***2-oxo-2-*** ((***3,4,5-trimethoxyphenyl***) ***amino***) ***ethyl***) ***penta-2,4-dienamide*** (**D8**): red solid, 183.3 mg, yield 41.6%, mp: 189.3–192.9°C, ^1^H NMR (600 MHz, DMSO-*d*_*6*_) δ = 9.92 (s, 1H, -NH), 8.36 (t, *J* = 5.7 Hz, 1H, -NH), 7.24 (s, 1H, Ar-H), 7.18–7.14 (m, 1H, Ar-H), 6.99–6.94 (m, 3H, Ar-H), 6.93–6.84 (m, 3H, -CH = CH-), 6.18 (d, *J* = 15.0 Hz, 1H, -CH = CH-), 6.01 (s, 2H, -CH_2_), 3.92 (d, *J* = 5.8 Hz, 2H, -CH_2_), 3.70 (s, 6H, -CH_3_), 3.59 (s, 3H, -CH_3_). ^13^C NMR (151 MHz, DMSO-*d*_*6*_) δ = 168.08, 166.14, 153.15, 148.36, 148.19, 140.28, 138.61, 135.43, 131.25, 125.62, 124.50, 123.09, 108.87, 106.13, 101.68, 97.52, 60.54, 56.17, 43.32. HRMS (ESI) calcd for C_23_H_25_O_7_N_2_ [M + H]^+^: 441.16563, found 441.16418.

***(2E,4E)-5-*** (***Benzo[d]*** [***1,3***] ***dioxol-5-yl***) ***-N-*** (***2-*** ((***2-fluorophenyl***) ***amino***) ***-2-oxoethyl***) ***penta-2,4-dien-amide*** (**D9**): white solid, 122.4 mg, yield 33%, mp: 185.5–188.9°C, ^1^H NMR (600 MHz, DMSO-*d*_*6*_) δ = 9.77 (s, 1H, -NH), 8.41 (t, *J* = 5.7 Hz, 1H, -NH), 7.85 (d, *J* = 6.6 Hz, 1H, Ar-H), 7.32–7.06 (m, 5H, Ar-H), 6.99–6.82 (m, 4H, Ar-H and -CH = CH-), 6.18 (d, *J* = 15.0 Hz, 1H, -CH = CH-), 6.02 (s, 2H, -CH_2_), 4.03 (d, *J* = 5.8 Hz, 2H, -CH_2_). ^13^C NMR (151 MHz, DMSO-*d*_*6*_) δ = 168.74, 166.19, 154.01 (d, ^1^ *J*_*C-F*_ = 246.1 Hz), 148.37, 148.21, 140.36, 138.64, 131.27, 126.36 (d, ^2^ *J*_*C-F*_ = 12.1 Hz), 125.64, 124.78, 124.76, 124.44, 123.08, 115.95, 115.82, 108.86, 106.15, 101.68, 43.16.^19^F NMR (564 MHz, DMSO) δ = -124.94. HRMS (ESI) calcd for C_20_H_18_O_4_N_2_F [M + H]^+^: 369.12451, found 369.12305.

***(2E,4E)-5-*** (***Benzo[d]*** [***1,3***] ***dioxol-5-yl***) ***-N-*** (***2-*** ((***3-fluorophenyl***) ***amino***) ***-2-oxoethyl***) ***penta-2,4-dien-amide*** (**D10**): red solid, 67.1 mg, yield 18.2%, mp: 226.0–227.5°C, ^1^H NMR (600 MHz, DMSO-*d*_*6*_) δ = 10.22 (s, 1H, -NH), 8.41 (s, 1H, -NH), 7.56 (d, *J* = 11.6 Hz, 1H, Ar-H), 7.30 (dt, *J* = 17.2, 7.9 Hz, 2H, Ar-H), 7.24 (s, 1H, Ar-H), 7.18–7.14 (m, 1H, Ar-H), 6.97–6.83 (m, 5H, Ar-H and -CH = CH-), 6.18 (d, *J* = 15.0 Hz, 1H, -CH = CH-), 6.01 (s, 2H, -CH_2_), 3.96 (d, *J* = 5.6 Hz, 2H, -CH_2_). ^13^C NMR (151 MHz, DMSO-*d*_*6*_) δ = 168.66, 166.19, 162.57 (d, ^1^ *J*_*C-F*_ = 241.6 Hz), 148.37, 148.20, 141.04 (d, ^2^ *J*_*C-F*_ = 12.1 Hz), 140.34, 138.64, 131.26, 130.8 (d, ^3^ *J*_*C-F*_ = 9.1 Hz), 125.63, 124.43, 123.08, 115.31, 110.12 (d, ^2^ *J*_*C-F*_ = 21.2 Hz), 108.87, 106.37 (d, ^2^ *J*_*C-F*_ = 27.2 Hz), 106.15, 101.68, 43.36.^19^F NMR (564 MHz, DMSO) δ = -112.05. HRMS (ESI) calcd for C_20_H_18_O_4_N_2_F [M + H]^+^: 369.12451, found 369.12344.

***(2E,4E)-5-*** (***Benzo[d]*** [***1,3***] ***dioxol-5-yl***) ***-N-*** (***2-*** ((***4-fluorophenyl***) ***amino***) ***-2-oxoethyl***) ***penta-2,4-dien- amide*** (**D11**): white solid, 120.0 mg, yield 32.6%. mp: 251.8–253.6°C ^1^H NMR (600 MHz, DMSO-*d*_*6*_) δ = 10.05 (s, 1H, -NH), 8.40 (t, *J* = 5.8 Hz, 1H, -NH), 7.59–7.57 (m, 2H, Ar-H), 7.24 (d, *J* = 1.2 Hz, 1H, Ar-H), 7.14 (dt, *J* = 17.7, 9.8 Hz, 3H, Ar-H), 6.98–6.84 (m, 4H, Ar-H and -CH = CH-), 6.18 (d, *J* = 15.0 Hz, 1H, -CH = CH-), 6.02 (s, 2H, -CH_2_), 3.94 (d, *J* = 5.8 Hz, 2H, -CH_2_). ^13^C NMR (151 MHz, DMSO-*d*_*6*_) δ = 168.19, 166.11, 159.43 (d, ^1^ *J*_*C-F*_ = 240.1 Hz), 148.28 (d, ^2^ *J*_*C-F*_ = 27.3 Hz), 140.25, 138.59, 135.71, 131.27, 125.65, 124.53, 123.07, 121.38, 115.70 (d, ^2^ *J*_*C-F*_ = 22.7 Hz), 109.99, 108.86, 106.14, 101.68, 43.25.^19^F NMR (564 MHz, DMSO) δ = -119.48. HRMS (ESI) calcd for C_20_H_18_O_4_N_2_F [M + H]^+^: 369.12451, found 369.12338.

***(2E,4E)-5-*** (***Benzo[d]*** [***1,3***] ***dioxol-5-yl***) ***-N-*** (***2-*** ((***3-chlorophenyl***) ***amino***) ***-2-oxoethyl***) ***penta-2,4-dien-amide*** (**D12**): yellow solid, 82.5 mg, yield 21.5% mp: 223.8–225.7°C, ^1^H NMR (600 MHz, DMSO-*d*_*6*_) δ = 10.19 (s, 1H, -NH), 8.41 (t, *J* = 5.7 Hz, 1H, -NH), 7.77 (s, 1H, Ar-H), 7.43 (d, *J* = 8.2 Hz, 1H, Ar-H), 7.31 (t, *J* = 8.1 Hz, 1H, Ar-H), 7.24 (s, 1H, Ar-H), 7.18–7.14 (m, 1H, Ar-H), 7.08 (d, *J* = 8.0 Hz, 1H, Ar-H), 6.98–6.83 (m, 4H, Ar-H and -CH = CH-), 6.18 (d, *J* = 15.1 Hz, 1H, -CH = CH-), 6.02 (s, 2H, -CH_2_), 3.95 (d, *J* = 5.8 Hz, 2H, -CH_2_). ^13^C NMR (151 MHz, CDCl_3_) δ = 173.45, 170.92, 153.12, 152.95, 145.52, 145.07, 143.39, 138.25, 136.02, 135.62, 130.39, 129.19, 128.14, 127.84, 123.80, 122.72, 113.62, 110.90, 106.43, 48.12. HRMS (ESI) calcd for C_20_H_18_O_4_N_2_Cl [M + H]^+^: 385.09496, found 385.09415.

***(2E,4E)-N-*** (***2-*** ((***3-Acetylphenyl***) ***amino***) ***-2-oxoethyl***) ***-5-*** (***benzo[d]*** [***1,3***] ***dioxol-5-yl***) ***penta-2,4-dien-amide* (D13)**: brown solid, 137 mg, yield 35.0%, mp: >250.0°C, ^1^H NMR (600 MHz, DMSO-*d*_*6*_) δ = 10.20 (s, 1H, -NH), 8.40 (s, 1H, -NH), 8.15 (s, 1H, Ar-H), 7.83 (d, *J* = 7.6 Hz, 1H, Ar-H), 7.64 (d, *J* = 7.4 Hz, 1H, Ar-H), 7.45 (t, *J* = 7.8 Hz, 1H, Ar-H), 7.24 (s, 1H, Ar-H), 6.97 (d, *J* = 7.4 Hz, 2H, Ar-H), 6.90–6.83 (m, 3H, Ar-H and -CH = CH-), 6.19 (d, *J* = 15.0 Hz, 1H, -CH = CH-), 6.02 (s, 2H, -CH_2_), 3.98 (d, *J* = 5.3 Hz, 2H, -CH_2_), 2.53 (s, 3H, -CH_3_).^13^C NMR (151 MHz, DMSO-*d*_*6*_) δ = 198.01, 168.62, 166.16, 148.37, 148.20, 140.29, 139.70, 138.62, 137.82, 131.27, 129.60, 125.64, 124.50, 124.09, 123.72, 123.08, 118.84, 108.86, 106.15, 101.68, 43.37, 27.11. HRMS (ESI) calcd for C_22_H_21_O_5_N_2_ [M + H]^+^: 393.14450, found 393.14301.

***(2E,4E)-5-*** (***Benzo[d]*** [***1,3***] ***dioxol-5-yl***) ***-N-*** (***2-*** ((***4-*** (***methylthio***) ***phenyl***) ***amino***) ***-2-oxoethyl***) ***penta-2,4-dienamide* (D14)**: brown solid, 54.5 mg, yield 13.7%, mp: 208.9–211.3°C, ^1^H NMR (600 MHz, DMSO-*d*_*6*_) δ = 10.01 (s, 1H, -NH), 8.39 (t, *J* = 5.7 Hz, 1H, -NH), 7.53 (d, *J* = 8.6 Hz, 2H, Ar-H), 7.23 (s, 1H, Ar-H), 7.22–7.18 (m, 2H, Ar-H), 7.17–7.10 (m, 1H, Ar-H), 6.99–6.95 (m, 1H, Ar-H), 6.89 (m, 3H, -CH = CH-), 6.18 (d, *J* = 15.0 Hz, 1H, -CH = CH-), 6.01 (s, 2H, -CH_2_), 3.95 (d, *J* = 5.8 Hz, 2H, -CH_2_), 2.41 (s, 3H, -CH_3_). ^13^C NMR (151 MHz, DMSO-*d*_*6*_) δ = 168.16, 166.14, 148.37, 148.19, 140.26, 138.58, 136.87, 132.24, 131.27, 127.67, 125.64, 124.54, 123.05, 120.33, 108.86, 106.15, 101.68, 43.31, 16.05. HRMS (ESI) calcd for C_21_H_21_O_4_N_2_S [M + H]^+^: 397.12165, found 397.12054.

***(2E,4E)-5-*** (***benzo[d]*** [***1,3***] ***dioxol-5-yl***) ***-N-*** (***2-*** ((***3-*** (***benzyloxy***) ***phenyl***) ***amino***) ***-2-oxoethyl***) ***penta-2,4-dienamide* (D15)**: red solid, 99.2 mg, yield 21.7%, mp: 191.0–192.0°C, ^1^H NMR (600 MHz, DMSO) δ = 9.97 (s, 1H, -NH), 8.37 (t, *J* = 5.5 Hz, 1H, -NH), 7.41 (d, *J* = 7.6 Hz, 2H, Ar-H), 7.36 (t, *J* = 6.8 Hz, 3H, Ar-H), 7.30 (t, *J* = 6.8 Hz, 1H, Ar-H), 7.24 (s, 1H, Ar-H), 7.17 (dt, *J* = 15.2, 9.2 Hz, 2H, Ar-H), 7.10 (d, *J* = 7.9 Hz, 1H, Ar-H), 6.97 (d, *J* = 8.1 Hz, 1H, Ar-H), 6.95–6.78 (m, 4H, Ar-H and -CH = CH-), 6.69 (d, *J* = 8.0 Hz, 1H, -CH = CH-), 6.18 (d, *J* = 15.0 Hz, 1H, Ar-H), 6.02 (s, 2H, -CH_2_), 5.04 (s, 2H, -CH_2_), 3.95 (d, *J* = 5.8 Hz, 2H, -CH_2_). ^13^C NMR (151 MHz, DMSO-*d*_*6*_) δ = 168.31, 166.10, 159.06, 148.37, 148.19, 140.53, 140.25, 138.59, 137.49, 131.28, 129.97, 128.84, 128.21, 128.01, 125.65, 124.53, 123.09, 112.15, 109.97, 108.87, 106.38, 106.13, 101.68, 69.60, 43.34. HRMS (ESI) calcd for C_27_H_25_O_5_N_2_ [M + H]^+^: 457.17580, found 457.17459.

***(2E,4E)-5-*** (***Benzo[d]*** [***1,3***] ***dioxol-5-yl***) ***-N-*** (***2-*** ((***4-*** (***benzyloxy***) ***phenyl***) ***amino***) ***-2-oxoethyl***) ***penta-2,4-dienamide* (D16)**: yellow solid, 97.6 mg, yield 21.3%, mp: 231.4–233.5°C, ^1^H NMR (600 MHz, DMSO) δ = 9.86 (s, 1H, -NH), 8.37 (t, *J* = 5.6 Hz, 1H, -NH), 7.47 (d, *J* = 8.9 Hz, 2H, Ar-H), 7.41 (d, *J* = 7.4 Hz, 2H, Ar-H), 7.35 (dt, *J* = 7.1, 5.0 Hz, 3H, Ar-H), 7.29 (t, *J* = 7.1 Hz, 1H, Ar-H), 7.24 (s, 1H, Ar-H), 6.97 (d, *J* = 7.7 Hz, 1H, Ar-H), 6.95–6.85 (m, 5H, Ar-H and -CH = CH-), 6.18 (d, *J* = 15.0 Hz, 1H, -CH = CH-), 6.01 (s, 2H, -CH = CH-), 5.03 (s, 2H, -CH_2_), 3.92 (d, *J* = 5.8 Hz, 2H, -CH_2_). ^13^C NMR (151 MHz, DMSO-*d*_*6*_) δ = 167.76, 166.11, 154.75, 148.36, 148.19, 140.21, 138.55, 137.63,132.69, 131.27, 128.81, 128.06, 125.65, 124.60, 123.05, 121.16, 116.24, 115.35, 108.87, 106.15, 101.68, 69.89, 43.21. HRMS (ESI) calcd for C_27_H_25_O_5_N_2_ [M + H]^+^: 457.17580, found 457.17438.

***(2E,4E)-5-*** (***Benzo[d]*** [***1,3***] ***dioxol-5-yl***) ***-N-*** (***2-*** ((***4-*** ((***2,6-difluorobenzyl***) ***oxy***) ***phenyl***) ***amino***) ***-2-oxoethyl***) ***penta-2,4-dienamide* (D17)**: yellow solid, 89.7 mg, yield 18.2%, mp: 203.9–206.5°C, ^1^H NMR (600 MHz, DMSO-*d*_*6*_) δ = 9.89 (s, 1H, -NH), 8.38 (s, 1H, -NH), 7.49 (d, *J* = 8.9 Hz, 3H, Ar-H), 7.24 (s, 1H, Ar-H), 7.17–7.13 (m, 3H, Ar-H), 6.97 (t, *J* = 7.9 Hz, 3H, Ar-H), 6.92–6.84 (m, 3H, -CH = CH-), 6.19 (d, *J* = 15.0 Hz, 1H, -CH = CH-), 6.02 (s, 2H, -CH_2_), 5.04 (s, 2H, -CH_2_), 3.94 (d, *J* = 5.7 Hz, 2H, -CH_2_). ^13^C NMR (151 MHz, DMSO-*d*_*6*_) δ = 167.83, 166.08, 162.49, 160.84, 154.46, 148.28 (d, ^2^ *J*_*C-F*_ = 28.3 Hz), 140.21, 138.56, 133.17, 132.05, 131.28, 125.66, 124.59, 123.09, 121.10, 115.92 (d, ^1^ *J*_*C-F*_ = 199.3 Hz), 115.42, 112.18 (dd, *J* = 21.2, 4.5 Hz), 108.86, 106.13, 101.68, 58.38, 43.21.^19^F NMR (564 MHz, DMSO) δ = -115.17. HRMS (ESI) calcd for C_27_H_23_O_5_N_2_F_2_ [M + H]^+^: 493.15695, found 493.15533.

***(2E,4E)-5-*** (***Benzo[d]*** [***1,3***] ***dioxol-5-yl***) ***-N-*** (***2-oxo-2-*** ((***4-*** ((***4-*** (***trifluoromethyl***) ***benzyl***) ***oxy***) ***phenyl***) ***-amino***) ***ethyl***) ***penta-2,4-dienamide* (D18)**: brown solid, 118.5 mg, yield 22.5%, mp: 200.6–202.7°C, ^1^H NMR (600 MHz, DMSO-*d*_*6*_) δ = 9.90 (s, 1H, -NH), 8.39 (s, 1H, -NH), 7.72 (d, *J* = 7.4 Hz, 2H, Ar-H), 7.63 (d, *J* = 7.5 Hz, 2H, Ar-H), 7.50–7.46 (m, 2H, Ar-H), 7.24 (s, 1H, Ar-H), 7.19–7.13 (m, 1H, Ar-H), 6.96 (t, *J* = 8.7 Hz, 3H, Ar-H), 6.91–6.83 (m, 3H, -CH = CH-), 6.19 (d, *J* = 15.0 Hz, 1H, -CH = CH-), 6.02 (s, 2H, -CH_2_), 5.16 (s, 2H, -CH_2_), 3.94 (d, *J* = 5.6 Hz, 2H, -CH_2_). ^13^C NMR (151 MHz, DMSO-*d*_*6*_) δ = 167.81, 166.09, 154.39, 148.37, 148.19, 142.54, 140.21, 138.56, 132.96, 131.27, 128.38, 125.68 (t, ^4^ *J*_*C-F*_ = 4.53 Hz), 124.59, 123.08, 121.15, 115.37, 108.85, 106.12, 101.68, 68.96, 43.21.^19^F NMR (564 MHz, DMSO) δ = -60.97. HRMS (ESI) calcd for C_28_H_24_O_5_N_2_F_3_ [M + H]^+^: 525.16318, found 525.16119.

***(2E,4E)-5-*** (***Benzo[d]*** [***1,3***] ***dioxol-5-yl***) ***-N-*** (***2-oxo-2-*** ((***4-*** (***pyridin-2-ylmethoxy***) ***phenyl***) ***amino***) ***ethyl***) ***-penta-2,4-dienamide* (D19)**: brown solid, 95.2 mg, yield 20.8%, mp: 211.2–214.5°C ^1^H NMR (600 MHz, DMSO-*d*_*6*_) δ = 9.86 (s, 1H, -NH), 8.54 (d, *J* = 4.1 Hz, 1H, -NH), 8.36 (t, *J* = 5.5 Hz, 1H, Ar-H), 7.80 (td, *J* = 7.7, 1.7 Hz, 1H, Ar-H), 7.48 (d, *J* = 8.9 Hz, 3H, Ar-H), 7.33–7.29 (m, 1H, Ar-H), 7.24 (d, *J* = 1.2 Hz, 1H, Ar-H), 7.18–7.14 (m, 1H, Ar-H), 7.00–6.79 (m, 7H, Ar-H and -CH = CH-), 6.18 (d, *J* = 15.0 Hz, 1H, -CH = CH-), 6.02 (s, 2H, -CH_2_), 5.11 (s, 2H, -CH_2_), 3.93 (d, *J* = 5.8 Hz, 2H, -CH_2_). ^13^C NMR (151 MHz, DMSO-*d*_*6*_) δ = 167.79, 166.07, 157.25, 154.51, 149.49, 148.37, 148.18, 140.20, 138.56, 137.34, 132.88, 131.28, 125.66, 124.60, 123.32, 123.08, 122.05, 121.17, 115.30, 108.86, 106.13, 101.68, 70.95, 43.20. HRMS (ESI) calcd for C_26_H_24_O_5_N_3_ [M + H]^+^: 458.17105, found 458.16992.

***(2E,4E)-5-*** (***Benzo[d]*** [***1,3***] ***dioxol-5-yl***) ***-N-*** (***2-*** ((***3-*** (***benzyloxy***) ***phenyl***) ***amino***) ***-2-oxoethyl***) ***penta-2,4-dienamide* (D20)**: brown solid, 110.4 mg, yield 24.8%, mp: 182.9–185.0°C ^1^H NMR (600 MHz, DMSO-*d*_*6*_) δ = 10.02 (s, 1H, -NH), 8.39 (t, *J* = 5.6 Hz, 1H, -NH), 7.58 (d, *J* = 8.8 Hz, 2H, Ar-H), 7.33 (t, *J* = 7.9 Hz, 2H, Ar-H), 7.24 (s, 1H, Ar-H), 7.18–7.14 (m, 1H, Ar-H), 7.07 (t, *J* = 7.3 Hz, 1H, Ar-H), 7.02–6.82 (m, 8H, Ar-H and -CH = CH-), 6.19 (d, *J* = 15.0 Hz, 1H, -CH = CH-), 6.02 (s, 2H, -CH_2_), 3.95 (d, *J* = 5.7 Hz, 2H, -CH_2_). ^13^C NMR (151 MHz, DMSO-*d*_*6*_) δ = 168.08, 166.12, 157.78, 152.22, 148.37, 148.19, 140.24, 138.58, 135.24, 131.28, 130.36, 125.66, 124.56, 123.40, 123.06, 121.31, 119.85, 118.32, 108.86, 106.15, 101.68, 43.28. HRMS (ESI) calcd for C_26_H_23_O_5_N_2_ [M + H]^+^: 443.16015, found 443.15891.

***(2E,4E)-5-*** (***Benzo[d]*** [***1,3***] ***dioxol-5-yl***) ***-N-*** (***2-*** (***benzylamino***) ***-2-oxoethyl***) ***penta-2,4-dienamide* (D21)**: red solid, 62.2 mg, yield 17.1%, mp: 173.6–175.8°C, ^1^H NMR (600 MHz, DMSO-*d*_*6*_) δ = 8.38 (t, *J* = 5.3 Hz, 1H, -NH), 8.33 (t, *J* = 5.6 Hz, 1H, -NH), 7.29 (t, *J* = 7.4 Hz, 2H, Ar-H), 7.26–7.07 (m, 5H, Ar-H), 6.97 (d, *J* = 7.9 Hz, 1H, Ar-H), 6.94–6.82 (m, 3H, -CH = CH-), 6.15 (d, *J* = 15.0 Hz, 1H, -CH = CH-), 6.01 (s, 2H, -CH_2_), 4.27 (d, *J* = 5.8 Hz, 2H, -CH_2_), 3.81 (d, *J* = 5.7 Hz, 2H, -CH_2_). ^13^C NMR (151 MHz, DMSO-*d*_*6*_) δ = 169.42, 166.04, 148.37, 148.18, 140.10, 139.80, 138.50, 131.28, 128.64, 127.63, 127.15, 125.66, 124.73, 123.04, 108.86, 106.13, 101.68, 42.72, 42.52. HRMS (ESI) calcd for C_21_H_21_O_4_N_2_ [M + H]^+^: 365.14958, found 365.14828.

***(2E,4E)-5-*** (***Benzo[d]*** [***1,3***] ***dioxol-5-yl***) ***-N-*** (***2-*** ((***2-chlorobenzyl***) ***amino***) ***-2-oxoethyl***) ***penta-2,4-dienamide* (D22)**: red solid, 182 mg, yield 45.6%, mp: 180.1–183.0°C, ^1^H NMR (600 MHz, DMSO-*d*_*6*_) δ = 8.40 (s, 1H, -NH), 8.36 (t, *J* = 5.4 Hz, 1H, -NH), 7.40 (d, *J* = 7.6 Hz, 1H, Ar-H), 7.35–7.21 (m, 4H, Ar-H), 7.18–7.14 (m, 1H, Ar-H), 6.97 (d, *J* = 8.0 Hz, 1H, Ar-H), 6.95–6.79 (m, 3H, -CH = CH-), 6.15 (d, *J* = 15.0 Hz, 1H, -CH = CH-), 6.01 (s, 2H, -CH_2_), 4.32 (d, *J* = 5.7 Hz, 2H, -CH_2_), 3.84 (d, *J* = 5.8 Hz, 2H, -CH_2_). ^13^C NMR (151 MHz, DMSO-*d*_*6*_) δ = 169.72, 166.10, 148.37, 148.18, 140.14, 138.54, 136.66, 132.36, 131.27, 129.46, 129.18, 128.98, 127.53, 125.65, 124.67, 123.08, 108.86, 106.12, 101.68, 42.72, 40.43. HRMS (ESI) calcd for C_21_H_20_O_4_N_2_Cl [M + H]^+^: 399.11061, found 399.10913.

***(2E,4E)-5-*** (***Benzo[d]*** [***1,3***] ***dioxol-5-yl***) ***-N-*** (***2-oxo-2-*** (***phenethylamino***) ***ethyl***) ***penta-2,4-dienamide* (D23)**:white solid, 147.5 mg, yield 38.9%, mp: 166.5–168.4°C, ^1^H NMR (600 MHz, DMSO-*d*_*6*_) δ = 8.26 (t, *J* = 5.4 Hz, 1H, -NH), 7.93 (s, 1H, -NH), 7.27–7.23 (m, 3H, Ar-H), 7.21–7.09 (m, 4H, Ar-H), 7.00–6.82 (m, 4H, Ar-H and -CH = CH-), 6.15 (d, *J* = 15.0 Hz, 1H, -CH = CH-), 6.01 (s, 2H, -CH_2_), 3.73 (d, *J* = 5.8 Hz, 2H, -CH_2_), 3.29–3.25 (m, 2H, -CH_2_), 2.69 (t, *J* = 7.3 Hz, 2H, -CH_2_). ^13^C NMR (151 MHz, DMSO-*d*_*6*_) δ = 169.23, 165.96, 148.37, 148.18, 140.11, 139.83, 138.51, 131.28, 129.03, 128.75, 126.50, 125.65, 124.69, 123.07, 108.86, 106.12, 101.68, 42.69, 40.66, 35.62. HRMS (ESI) calcd for C_22_H_23_O_4_N_2_ [M + H]^+^: 379.16523, found 379.16385.

***(2E,4E)-5-*** (***Benzo[d]*** [***1,3***] ***dioxol-5-yl***) ***-N-*** (***2-oxo-2-*** ((***3-phenylpropyl***) ***amino***) ***ethyl***) ***penta-2,4-dienamide* (D24)**: white solid, 161.9 mg, yield 44.6%, mp: 166.5–168.4°C, ^1^H NMR (600 MHz, DMSO-*d*_*6*_) δ = 8.29 (t, *J* = 5.5 Hz, 1H, -NH), 7.90 (d, *J* = 4.9 Hz, 1H, -NH), 7.30–7.22 (m, 3H, Ar-H), 7.20–7.09 (m, 4H, Ar-H), 6.98–6.94 (m, 1H, Ar-H), 6.94–6.77 (m, 3H, -CH = CH-), 6.16 (d, *J* = 15.0 Hz, 1H, -CH = CH-), 6.01 (s, 2H, -CH_2_), 3.74 (d, *J* = 5.8 Hz, 2H, -CH_2_), 3.08–3.04 (m, 2H, -CH_2_), 2.57–2.51 (m, 2H, -CH_2_), 1.73–1.63 (m, 2H, -CH_2_). ^13^C NMR (151 MHz, DMSO-*d*_*6*_) δ = 169.20, 165.95, 148.36, 148.17, 142.15, 140.05, 138.47, 131.28, 128.70, 128.68, 126.12, 125.66, 124.75, 123.05, 108.86, 106.12, 101.68, 42.71, 38.60, 32.89, 31.30. HRMS (ESI) calcd for C_23_H_25_O_4_N_2_ [M + H]^+^: 393.18088, found 393.17938.

***(2E,4E)-5-*** (***Benzo[d]*** [***1,3***] ***dioxol-5-yl***) ***-N-*** (***2-*** (***naphthalen-2-ylamino***) ***-2-oxoethyl***) ***penta-2,4-dienamide* (D25)**: brown solid, 93.3 mg, yield 23.31%, mp: 141.9–144.2°C ^1^H NMR (600 MHz, DMSO-*d*_*6*_) δ = 10.01 (s, 1H, -NH), 8.50 (s, 1H, -NH), 8.07 (d, *J* = 6.8 Hz, 1H, Ar-H), 7.91 (d, *J* = 6.7 Hz, 1H, Ar-H), 7.75 (d, *J* = 7.5 Hz, 1H, Ar-H), 7.58–7.46 (m, 3H, Ar-H), 7.24 (s, 1H, Ar-H), 6.92 (dt, *J* = 37.5, 12.0 Hz, 6H, Ar-H and -CH = CH-), 6.23 (d, *J* = 14.8 Hz, 1H, -CH = CH-), 6.01 (s, 2H, -CH_2_), 4.15 (s, 2H, -CH_2_). ^13^C NMR (151 MHz, DMSO-*d*_*6*_) δ = 169.14, 166.35, 148.37, 148.20, 140.32, 138.62, 134.15, 133.80, 131.27, 128.52, 128.21, 126.46, 125.98, 125.66, 124.59, 124.05, 123.17, 123.07, 115.91, 108.87, 107.96, 106.15, 101.69, 43.37. HRMS (ESI) calcd for C_24_H_21_O_4_N_2_ [M + H]^+^: 401.14958, found 401.14816.

***(2E,4E)-5-*** (***Benzo[d]*** [***1,3***] ***dioxol-5-yl***) ***-N-*** (***2-*** (***benzo[d]*** [***1,3***] ***dioxol-5-ylamino***) ***-2-oxoethyl***) ***penta-2,4-dienamide* (D26)**: black solid, 110.4 mg, yield 27.9%, mp: 198.5–201.9°C ^1^H NMR (600 MHz, DMSO-*d*_*6*_) δ = 9.91 (s, 1H, -NH), 8.37 (s, 1H, -NH), 7.28–7.21 (m, 2H, Ar-H), 7.18–7.14 (m, 1H, Ar-H), 6.99–6.91 (m, 3H, Ar-H), 6.90–6.86 (m, 2H, -CH = CH-), 6.83 (t, *J* = 5.8 Hz, 1H, -CH = CH-), 6.18 (d, *J* = 15.1 Hz, 1H, -CH = CH-), 6.02 (s, 2H, -CH_2_), 5.95 (s, 2H, -CH_2_), 3.92 (d, *J* = 5.7 Hz, 2H, -CH_2_). ^13^C NMR (151 MHz, DMSO-*d*_*6*_) δ = 167.88, 166.08, 148.37, 148.19, 147.47, 143.33, 140.22, 138.56, 133.74, 131.28, 125.65, 124.57, 123.06, 112.44, 108.86, 108.44, 106.14, 101.82, 101.68, 101.37, 43.24. HRMS (ESI) calcd for C_21_H_19_O_6_N_2_ [M + H]^+^: 395.12376, found 395.12210.

***(2E,4E)-5-*** (***Benzo[d]*** [***1,3***] ***dioxol-5-yl***) ***-N-*** (***2-*** ((***1-methyl-1H-indol-5-yl***) ***amino***) ***-2-oxoethyl***) ***penta-2,4-dienamide* (D27)**: yellow solid, 142.8 mg, yield 35.4%, mp: 218.4–221.1°C, ^1^H NMR (600 MHz, DMSO-*d*_*6*_) δ = 9.81 (s, 1H, -NH), 8.38 (t, *J* = 5.6 Hz, 1H, -NH), 7.84 (s, 1H, Ar-H), 7.33 (d, *J* = 8.8 Hz, 1H, Ar-H), 7.28–7.22 (m, 2H, Ar-H), 7.19–7.15 (m, 1H, Ar-H), 7.00–6.82 (m, 4H, Ar-H and -CH = CH-), 6.34 (d, *J* = 2.8 Hz, 1H, -CH = CH-), 6.20 (d, *J* = 15.0 Hz, 1H, -CH = CH-), 6.02 (s, 2H, -CH_2_), 3.97 (d, *J* = 5.8 Hz, 2H, -CH_2_), 3.73 (s, 3H, -CH_3_).^13^C NMR (151 MHz, DMSO-*d*_*6*_) δ = 167.62, 166.07, 148.37, 148.18, 140.15, 138.52, 133.78, 131.47, 131.30, 130.56, 128.26, 125.69, 124.70, 123.06, 115.24, 111.42, 109.87, 108.86, 106.14, 101.68, 100.65, 43.33, 32.92. HRMS (ESI) calcd for C_23_H_22_O_4_N_3_ [M + H]^+^: 404.16048, found 404.15900.

***(2E,4E)-5-*** (***Benzo[d]*** [***1,3***] ***dioxol-5-yl***) ***-N-*** (***2-oxo-2-*** ((***2,2,2-trifluoroethyl***) ***amino***) ***ethyl***) ***penta-2,4-dienamide* (D28)**: yellow solid, 70.5 mg, yield 19.7%, mp: 154.2–156.1°C ^1^H NMR (600 MHz, DMSO-*d*_*6*_) δ = 8.52 (t, *J* = 5.9 Hz, 1H, -NH), 8.32 (t, *J* = 5.6 Hz, 1H, -NH), 7.23 (s, 1H, Ar-H), 7.17–7.13 (m, 1H, Ar-H), 6.97 (d, *J* = 7.9 Hz, 1H, Ar-H), 6.91–6.87 (m, 3H, -CH = CH-), 6.14 (d, *J* = 15.0 Hz, 1H, -CH = CH-), 6.01 (s, 2H, -CH_2_), 3.90–3.86 (m, 2H, -CH_2_), 3.83 (d, *J* = 5.8 Hz, 2H, -CH_2_). ^13^C NMR (151 MHz, DMSO-*d*_*6*_) δ = 170.38, 166.05, 148.37, 148.19, 140.25, 138.59, 131.26, 125.62, 124.52, 123.07, 108.86, 106.13, 101.68, 42.33.^19^F NMR (564 MHz, DMSO) δ = -70.71. HRMS (ESI) calcd for C_16_H_16_O_4_N_2_F_3_ [M + H]^+^: 357.10567, found 357.10431.

#### 2.1.5 Preparation of intermediates F1 and F2

A mixture of intermediate **E** (10.0 mmol), glycine methyl ester hydrochloride (9.0 mmol, 1.13 g), HOBt (10.0 mmol, 1.35 g), DIEPA (10.0 mmol, 1.29 g), and 20 ml of DCM was stirred at 0°C for 15 min. Then, EDCI (10 mol, 1.92 g) was added, and the mixture was stirred at 25°C for 18 h. After the reaction was completed, the solvent was removed under reduced pressure. Then, a mixture of KOH (20.0 mmol, 1.12 g) and 30 ml of H_2_O/CH_3_OH/THF (*v*:*v*:*v* = 1:1:1) was added to the crude products, and the reaction mixture was stirred at room temperature for 12 h. Then, 10% of HCl solution was added until a solid precipitate formed. The solvent was removed under reduced pressure, and the crude product was purified by column chromatography to obtain intermediates **F1** and **F2.**

***Cinnamoylglycine* (F1)**: white solid, 1.75 g, yield 85%, mp: 198.4–201.5°C, ^1^H NMR (600 MHz, DMSO-*d*_*6*_) δ = 8.42 (s, 1H, -NH), 7.55 (d, *J* = 7.4 Hz, 2H, Ar-H), 7.47–7.28 (m, 4H, Ar-H and -CH = CH-), 6.71 (d, *J* = 15.8 Hz, 1H, -CH = CH-), 3.87 (d, *J* = 5.1 Hz, 2H, -CH_2_). ^13^C NMR (151 MHz, DMSO-*d*_*6*_) δ = 171.64, 165.77, 139.63, 135.25, 129.95, 129.35, 127.99, 122.17, 41.30. HRMS (ESI) calcd for C_11_H_12_O_3_N [M + H]^+^: 206.08117, found 206.08066.

***(E)-*** (***3-*** (***3,4-Difluorophenyl***) ***acryloyl***) ***glycine* (F2)**: white solid, 1.98 g, yield 82%, mp: 210.0–213.1°C, ^1^H NMR (600 MHz, DMSO-*d*_*6*_) δ = 8.38 (s, 1H, -NH), 7.72–7.62 (m, 1H, Ar-H), 7.49–7.27 (m, 3H, Ar-H and -CH = CH-), 6.68 (d, *J* = 15.6 Hz, 1H, -CH = CH-), 3.87 (d, *J* = 3.0 Hz, 2H, -CH_2_). ^13^C NMR (151 MHz, DMSO-*d*_*6*_) δ = 171.58, 165.39, 151.07 (dd, *J* = 46.8, 12.1 Hz), 149.47 (dd, *J* = 45.3, 12.1 Hz), 137.52, 133.21 (dd, *J* = 6.0, 4.5 Hz), 125.19 (dd, *J* = 7.6, 4.5 Hz), 123.52, 118.42 (d, ^3^ *J*_*C-F*_ = 18.1 Hz), 116.69 (d, ^3^ *J*_*C-F*_ = 16.6 Hz), 41.31.^19^F NMR (564 MHz, DMSO) δ = -136.97, -138.11. HRMS (ESI) calcd for C_11_H_10_O_3_NF_2_ [M + H]^+^: 242.06233, found 242.06166.

#### 2.1.6 Preparation of target compounds J1 and J2

A mixture of intermediate **F** (1.0 mmol), amine (1.2 mmol), HOBt (1.2 mol, 162.14 mg), DIEPA (1.2 mol, 155.1 mg), and 10 ml of DCM was stirred at 0°C for 15 min. Then, EDCI (1.2 mol, 230.0 mg) was added, and the reaction mixture was stirred at 25°C for 18 h. After the reaction was completed, the solvent was removed under reduced pressure to give the crude product. Then, the target compounds **J1** and **J2** were obtained *via* recrystallization using ethyl acetate and petroleum ether as the solvent.

***N-*** (***2-oxo-2-*** ((***4-Phenoxyphenyl***) ***amino***) ***ethyl***) ***cinn-amamide* (J1)**: white solid, 58.2 mg, yield 15.6%, mp: 213.4–217.5°C, ^1^H NMR (600 MHz, DMSO-*d*_*6*_) δ = 10.07 (s, 1H, -NH), 8.45 (t, *J* = 5.5 Hz, 1H, -NH), 7.59–7.55 (m, 4H, Ar-H), 7.47–7.30 (m, 6H, Ar-H), 7.07 (t, *J* = 7.4 Hz, 1H, Ar-H), 6.97–6.93 (m, 4H, Ar-H), 6.76 (d, *J* = 15.8 Hz, 1H, -CH = CH-), 4.01 (d, *J* = 5.8 Hz, 2H, -CH_2_). ^13^C NMR (151 MHz, DMSO-*d*_*6*_) δ = 167.96, 165.88, 157.76, 152.24, 139.54, 135.28, 135.19, 130.37, 129.95, 129.37, 128.00, 123.42, 122.31, 121.34, 119.87, 118.32, 43.31. HRMS (ESI) calcd for C_23_H_21_O_3_N_2_ [M + H]^+^: 373.15467, found 373.15344.

***(E)-N-*** (***2-*** ((***4-*** (***benzyloxy***) ***phenyl***) ***amino***) ***-2-oxo-ethyl***) ***-3-*** (***3,4-difluorophenyl***) ***acrylamide* (J2)**: white solid, 49.5 mg, yield 11.7%, mp: 246.2–248.3°C, ^1^H NMR (600 MHz, DMSO-*d*_*6*_) δ = 9.90 (s, 1H, -NH), 8.39 (t, *J* = 5.6 Hz, 1H, Ar-H), 7.69–7.65 (m, 1H, Ar-H), 7.51–7.39 (m, 7H, Ar-H), 7.35 (t, *J* = 7.5 Hz, 2H, Ar-H), 7.29 (t, *J* = 7.2 Hz, 1H, Ar-H), 6.94 (d, *J* = 8.9 Hz, 2H, Ar-H), 6.75 (d, *J* = 15.8 Hz, 1H, -CH = CH-), 5.03 (s, 2H, -CH_2_), 3.98 (d, *J* = 5.6 Hz, 2H, -CH_2_). ^13^C NMR (151 MHz, DMSO-*d*_*6*_) δ = 167.53, 165.45, 154.77, 151.12 (dd, *J* = 43.8, 12.1 Hz), 149.48 (dd, *J* = 40.8, 13.6 Hz), 137.48 (d, ^2^ *J*_*C-F*_ = 39.3 Hz), 133.29 (dd, *J* = 6.0, 3.0 Hz), 132.64, 128.81, 128.32 (d, ^1^ *J*_*C-F*_ = 117.8 Hz), 128.18, 128.06, 125.15 (dd, *J* = 6.0, 3.0 Hz), 123.77, 121.16, 118.45 (d, ^2^ *J*_*C-F*_ = 18.1 Hz), 116.68 (d, ^2^ *J*_*C-F*_ = 18.1 Hz), 115.35, 69.87, 43.25.^19^F NMR (564 MHz, DMSO) δ = -137.01, -138.10.

### 2.2 Bioactivity assay against *P*. *xylostella*

The biological activity was evaluated using the leaf dipping method. The stock solution of insecticides was diluted using an aqueous solution of 0.05% Triton X-80. Cabbage leaf discs were dipped in solutions with the insecticide concentration (0.2 mg/ml) for 15 s and allowed to dry for 2 h. Control discs were treated with a 0.05% Triton X-80 solution. All the dipped leaf discs were dried at room temperature before being placed in Petri dishes (10 cm in diameter). Each set of concentrations was replicated three times. Next, 10 s-instar larvae were transferred to each Petri dish. The dishes were then stored in an incubator at 25 ± 2°C, 70 ± 20% RH (relative humidity) and kept under a 14:10 h light/dark photoperiod. Larvae mortality was recorded at 48 h.

### 2.3 Docking

A bioinformatics analysis for the molecular docking of **D28** with GABA_A_ receptor was conducted according to the method used by Sun et al. (2021). A homology model of GABA_A_ was constructed using the online server SWISS-MODEL (https://swissmodel.expasy.org/). Molecular docking of **D28** with GABA_A_ receptor was performed using Autodock software (version 4.2) (Bajaj et al., 1996). The energetically minimized three-dimensional structure of AITC was constructed using Chem 3D ultra 2010. The pdb files of **D28** and GABA_A_ receptor were set as the ligand and the receptor, respectively, followed by sequenced procedures using Autogrid and Autodock. Docking results with minimized reaction energy were selected, and binding sites were analyzed with PyMOL software (Seeliger and de Groot, 2010).

## 3 Results and discussion

### 3.1 Chemistry

**FIGURE 2:**
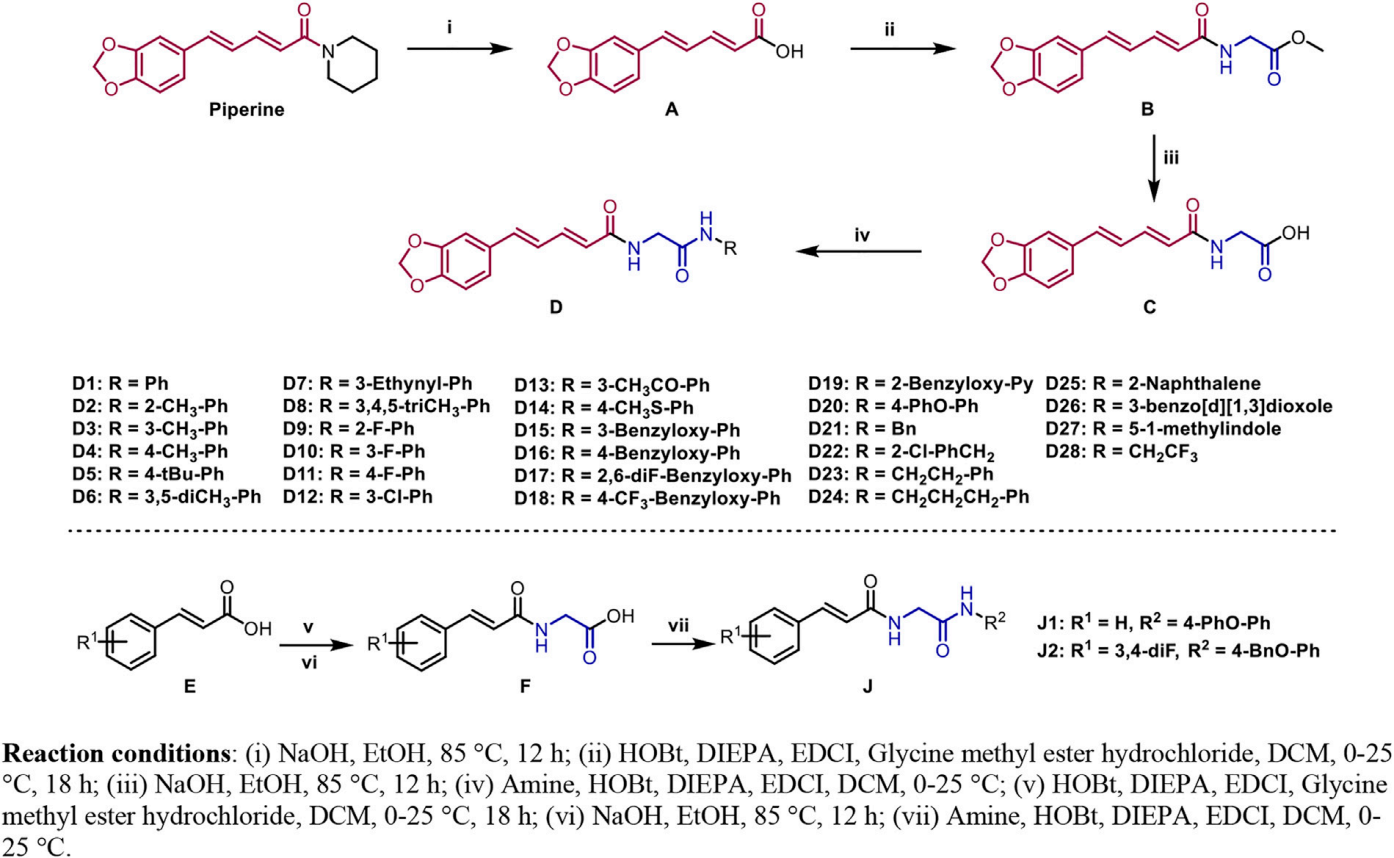
Synthesis route of the target compounds. Reaction conditions: (i) NaOH and EtOH, 85°C, 12 h; (ii) HOBt, DIEPA, EDCI, glycine methyl ester hydrochloride, and DCM, 0–25°C, 18 h; (iii) NaOH and EtOH, 85°C, 12 h; (iv) amine, HOBt, DIEPA, EDCI, and DCM, 0–25°C; (v) HOBt, DIEPA, EDCI, glycine methyl ester hydrochloride, and DCM, 0–25°C, 18 h; (vi) NaOH and EtOH, 85°C, 12 h; (vii) amine, HOBt, DIEPA, EDCI, and DCM, 0–25°C.

The synthetic routes of compounds **D1**–**D28**, **J1**, and **J2** are outlined in Figure 2. The pathway started from the reaction of piperine and NaOH in EtOH under 85°C for 12 h, which produced intermediate **A**. Then, intermediate **A** reacted with methyl glycinate to produce intermediate **B** in the presence of HOBt, DIPEA, and EDCI using CH_2_Cl_2_ as the solvent. Next, intermediate **B** reacted with KOH in H_2_O/CH_3_OH/THF (*v:v:v* = 1:1:1) to produce intermediate **C**. The target compounds **D1**–**D28** were obtained *via* a condensation reaction between intermediate **C** and different amines. The compounds **J1** and **J2** were prepared using **E1** and **E2** as the starting materials that was followed by two condensation reactions to form the target compounds **J1** and **J2**. All desired products were confirmed by ^1^H-NMR, ^13^C-NMR, ^19^F-NMR, and HRMS.

### 3.2 Insecticidal biological activity

The results of the insecticidal activity against *P. xylostella* are shown in Table 1. These compounds showed better insecticidal activities against *P. xylostella* than piperine, although most of the target compounds had low insecticidal activities with a mortality rate of less than 20% at a concentration of 0.2 mg/ml. Compounds **D12**, **D23**, and **D28** showed the highest activity among these compounds, with a mortality rate of 26.7, 26.7, and 43.3% at 48h, respectively. To further compare the difference in insecticidal activities of compound **D28** and piperine, the mortality rates of these two compounds were tested at 1 mg/ml (Table 2), and compound **D28** showed 90% mortality. From the structure–activity relationship, R showed significant effects on the insecticidal activities of the target compounds. When R was trifluoroethyl, compound **D28** showed the best insecticidal activities, followed by arenethyl. When chlorine was substituted at the 3-position of the benzene ring, compound **D12** showed higher insecticidal activity. When the substituent R was CH_3_, compounds with CH_3_ at the meta-position showed higher activity, for instance, **D3** (3-CH_3_) > **D2** (2-CH_3_) ≈ **D4** (4-CH_3_). Meanwhile, when R was benzyloxy-substituted phenyl, it seemed to have higher activity than alkyl-substituted phenyl. For instance, **D18** (4-CF_3_-Benzyloxy-Ph) > **D8** (3,4,5-triCH_3_-Ph) > **D21** (Bn).

**TABLE 1:** Insecticidal activity of piperine and target compounds against *P. xylostella* on larvae (mortality (%) ± SD) (48 h).

Compound	*Insecticidal activity against Plutella xylostella* ***^a^***
	0.2 mg/ml (%)
Piperine*^b^*	0
D1	13.8^a^± 1.9
D2	3.5 ± 1.9
D3	17.2 ± 3.3
D4	3.5 ± 3.9
D5	3.6 ± 0
D6	6.9 ± 0
D7	0
D8	13.3 ± 3.9
D9	13.3 ± 1.9
D10	20 ± 0
D11	6.9 ± 3.3
D12	26.7 ± 1.9
D13	3.5 ± 1.9
D14	3.5 + 1.9
D15	3.3 ± 1.9
D16	3.6 ± 0
D17	20.7 ± 1.9
D18	20.7 ± 1.9
D19	20.0 ± 0
D20	3.5 ± 1.9
D21	10.3 ± 1.9
D22	3.3 ± 1.9
D23	26.7 ± 1.9
D24	3.5 ± 1.9
D25	6.9 ± 3.3
D26	6.9 ± 3.3
D27	6.9 ± 3.3
D28	43.3 ± 1.9
J1	3.5 ± 1.9
J2	13.8 ± 1.9

a Average of three replicates.

b Piperine was used as control.

**TABLE 2:** Insecticidal activity of piperine and **D28** against *P. xylostella* at 1 mg/ml on larvae (mortality (%) ± SD) (48 h).

Compound	*Insecticidal activity against Plutella xylostella* *^a^*
	1 mg/ml (%)
Piperine^a^	0
D28	90.0 ± 0

a Average of three replicates.

### 3.3 Molecular docking of compound D28 with GABA_A_ receptor

**FIGURE 3:**
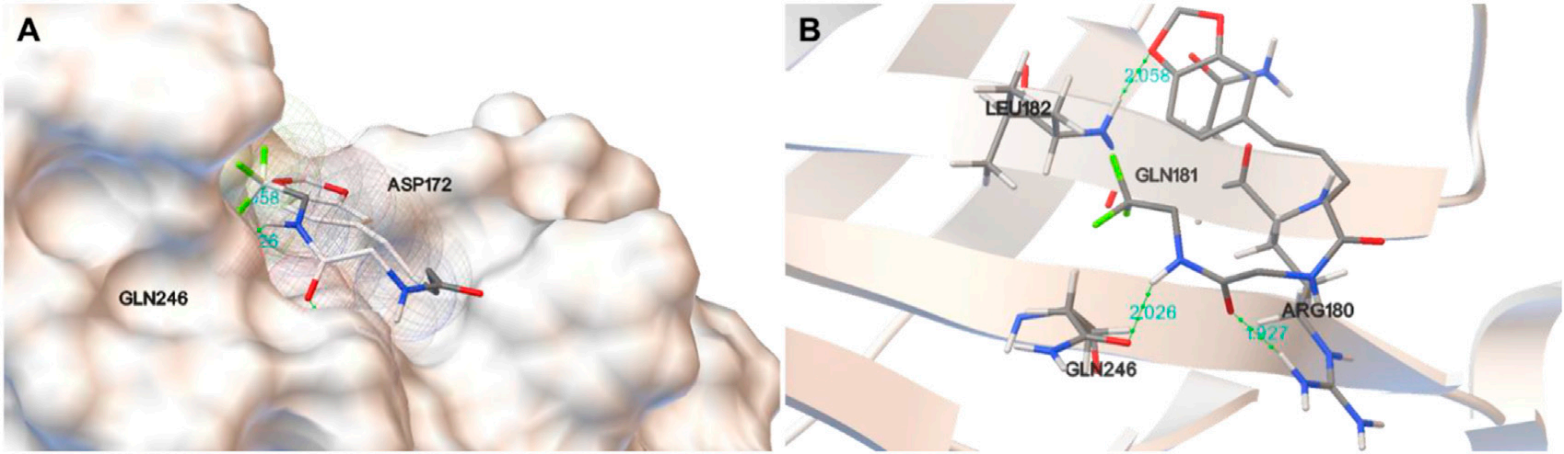
Molecular docking results of D28 with GABA_A_ receptor analyzed using PyMOL software. **(A)** Protein surface view for docking of D28 with GABA_A_ receptor with the interaction of a hydrogen bond; **(B)** picture of the docking of D28 with GABA_A_ receptor, with binding sites at ARG180, GLN246, and LEU182.

To identify the binding affinity of **D28** and GABA_A_ receptor, we determined the molecular docking of **D28** and GABA_A_ receptor using Autodock software (Figure 3). Figure 3A and b indicate that **D28** binds at ARG180, GLN246, and LEU182 within the GABA_A_ receptor by hydrogen bonds, of which distances were 1.927, 2.026, and 2.058 Å, respectively. The molecular docking results indicated that compound D28 could act on the GABA_A_ receptor.

## 4 Conclusion

In summary, 30 novel piperine derivatives containing linear bisamide were designed and synthesized. The structures of these compounds were confirmed *via* ^1^H-NMR, ^13^C-NMR, and HRMS. The insecticidal activities of these compounds were evaluated, and all of them have better insecticidal activities against *P. xylostella* than piperine. In addition, compound **D28** displayed good insecticidal activity. The insecticidal mechanism of compound **D28** was studied using molecular docking, and the results indicated that compound 34 may act on GABA_A_ receptors. These findings indicated that these piperine derivatives have the potential to be a promising lead compound for further study.

## Data Availability

The original contributions presented in the study are included in the article/Supplementary Material; further inquiries can be directed to the corresponding author.
