# Supplementary material for: Design, synthesis, and insecticidal activity evaluation of piperine derivatives
Source: Front Chem. 2022 Jul 26;10:973630. doi: 10.3389/fchem.2022.973630 (PMC9360595; doi:10.3389/fchem.2022.973630)
Supplement: Supplementary file 1 [file DataSheet1.pdf]

Supporting Information

for

Design, Synthesis, and Insecticidal Activity Evaluation of Piperine Derivatives

Chiying Zhang,[a‡] Qingqiang Tian[a‡] and Yahui Li[1,2]*

[1]Key Laboratory of Agri-Food Safety of Anhui Province, School of Resources and Environment, Anhui Agricultural University, Hefei 230036, China; E-mail: Yahui.Li @ahau. edu.cn.

[2]State Key Laboratory Breeding Base of Green Pesticide and Agricultural Bioengineering, Key Laboratory of Green Pesticide and Agricultural Bio-engineering, Ministry of Education, Guizhou University, Guiyang 550025, China

Contents

1. NMR Spectra .... **错误!未定义书签。**
2. HRMS Spectra 40

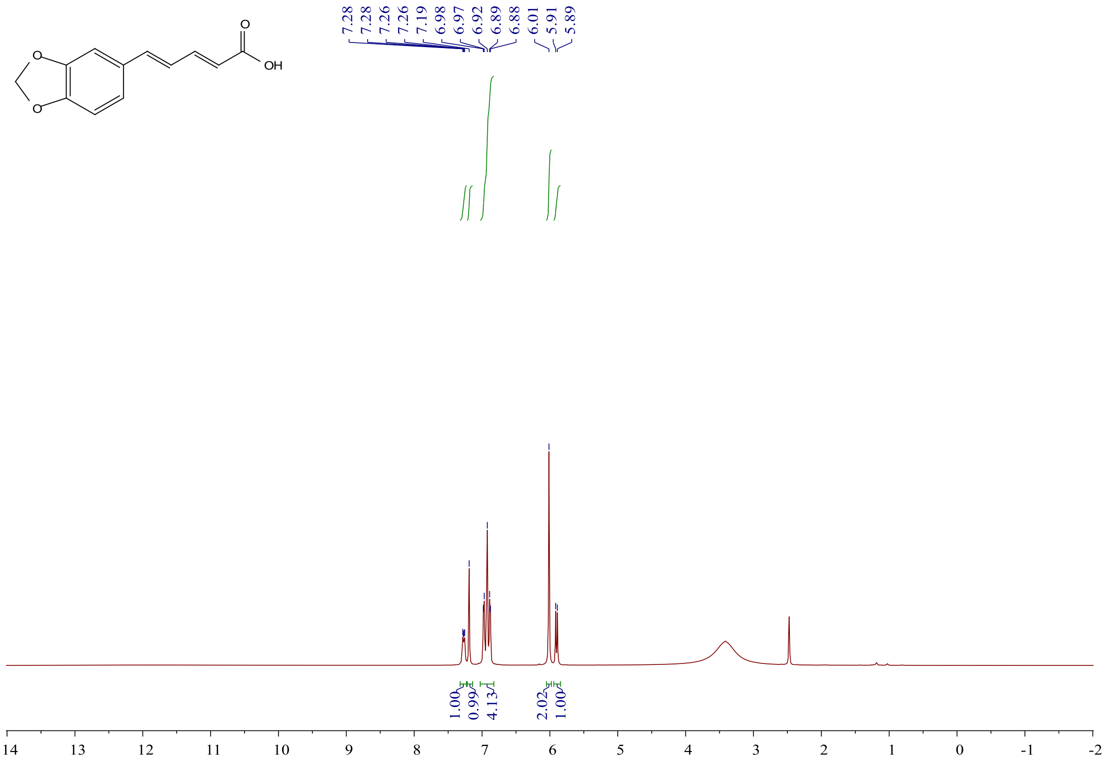
7.28
7.28
7.26
7.26
7.19
6.98
6.97
6.92
6.89
6.88
6.01
5.91
5.89
OH
O
1.00
0.99
4.13
2.02
1.00
14
13
12
11
10
9
8
7
6
5
4
3
2
1
0
-1
-2

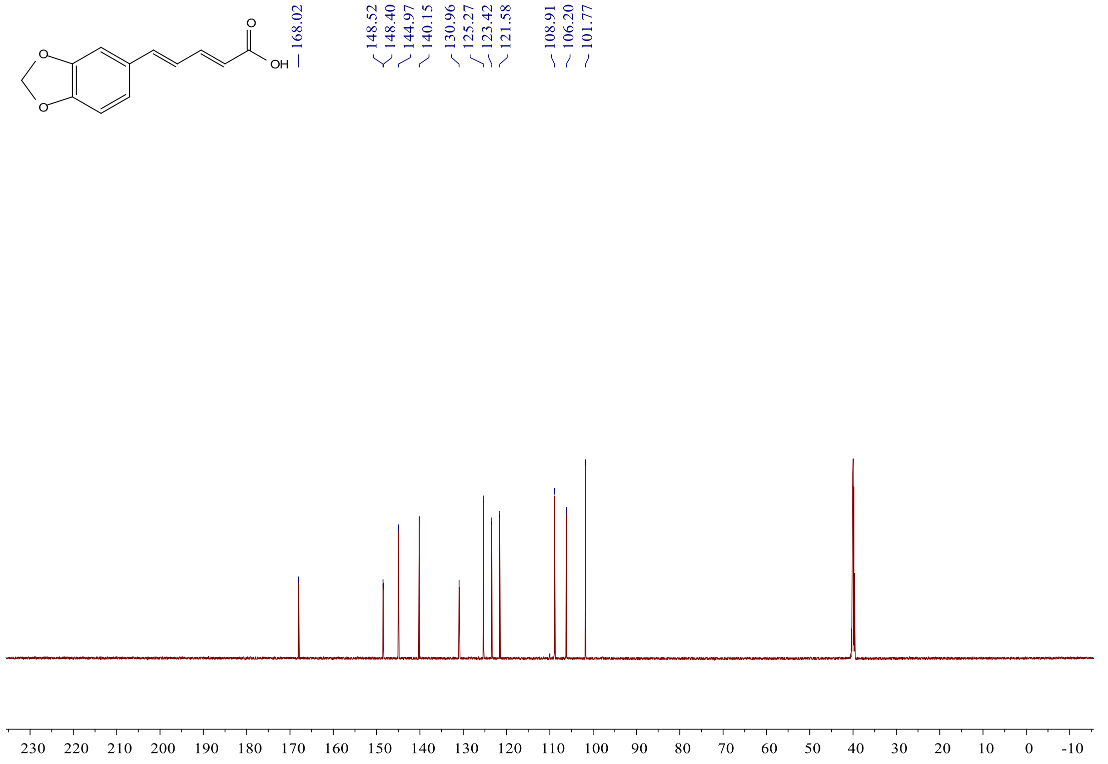
168.02
148.52
148.40
144.97
140.15
130.96
125.27
123.42
121.58
108.91
106.20
101.77
OH
O
230
220
210
200
190
180
170
160
150
140
130
120
110
100
90
80
70
60
50
40
30
20
10
0
-10

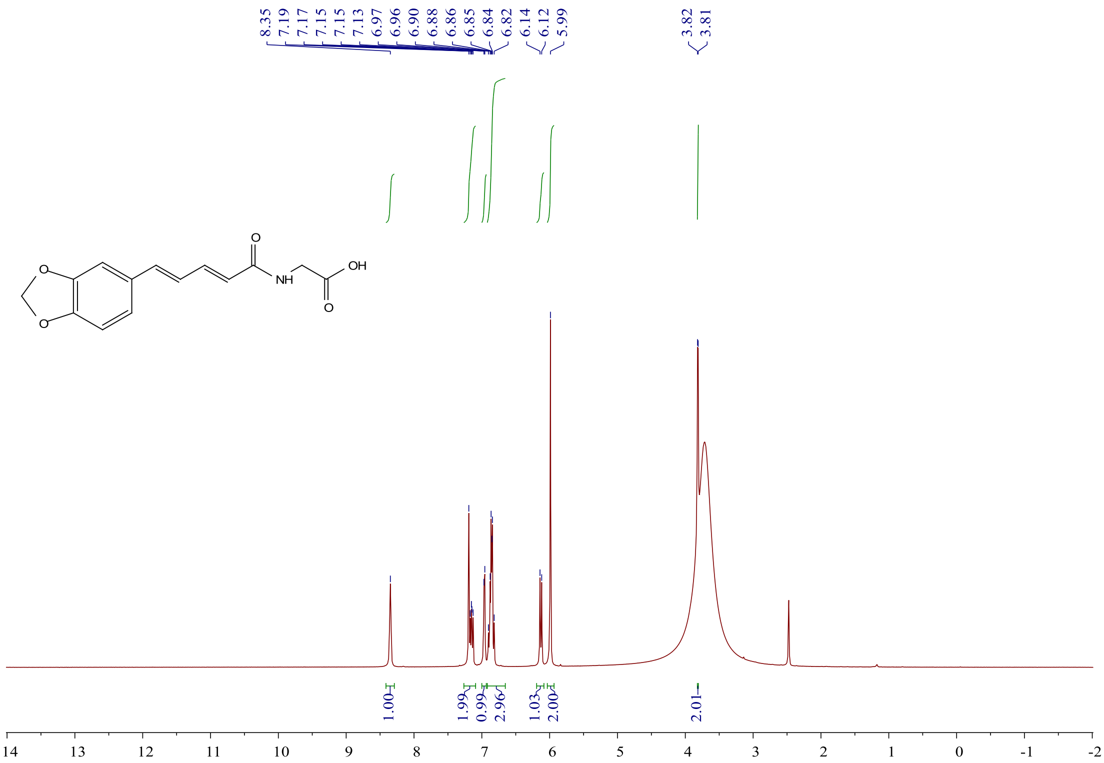
8.35
7.19
7.17
7.15
7.15
7.13
6.97
6.96
6.90
6.88
6.86
6.85
6.84
6.82
6.14
6.12
5.99
3.82
3.81
O
OH
NH
O
O
O
1.00
1.99
0.99
2.96
1.03
2.00
2.01
14 13 12 11 10 9 8 7 6 5 4 3 2 1 0 -1 -2

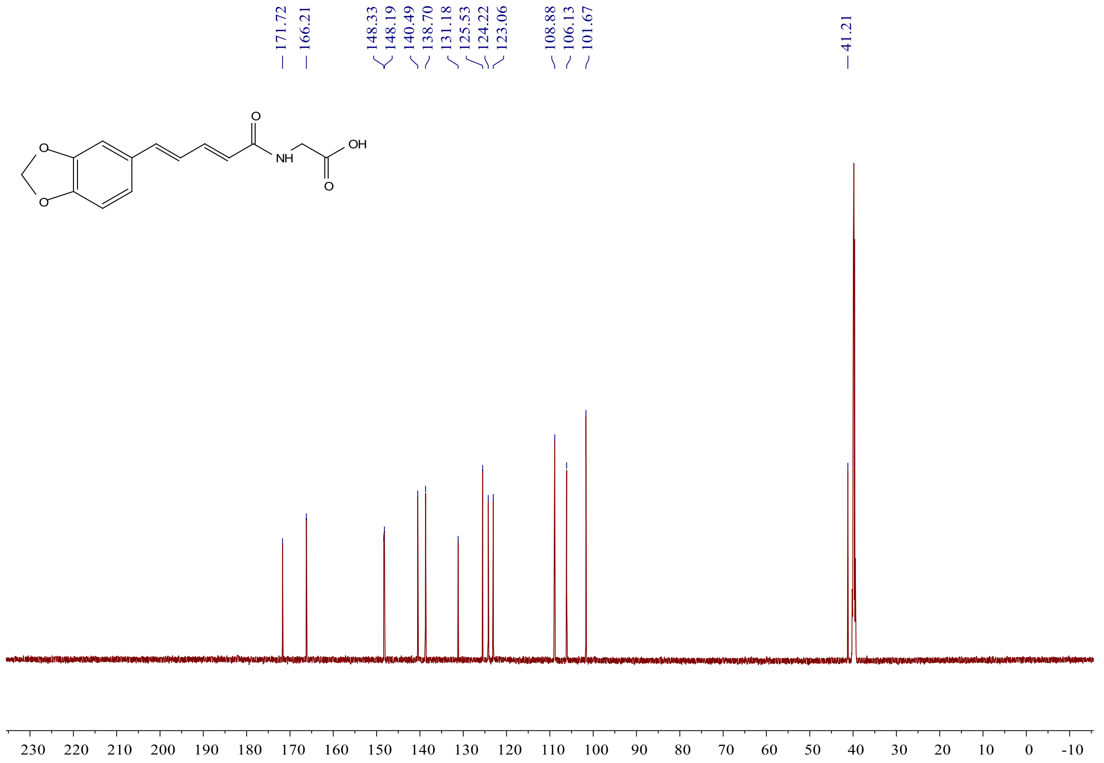
171.72
166.21
148.33
148.19
140.49
138.70
131.18
125.53
124.22
123.06
108.88
106.13
101.67
41.21
O
OH
NH
O
O
O
230 220 210 200 190 180 170 160 150 140 130 120 110 100 90 80 70 60 50 40 30 20 10 0 -10

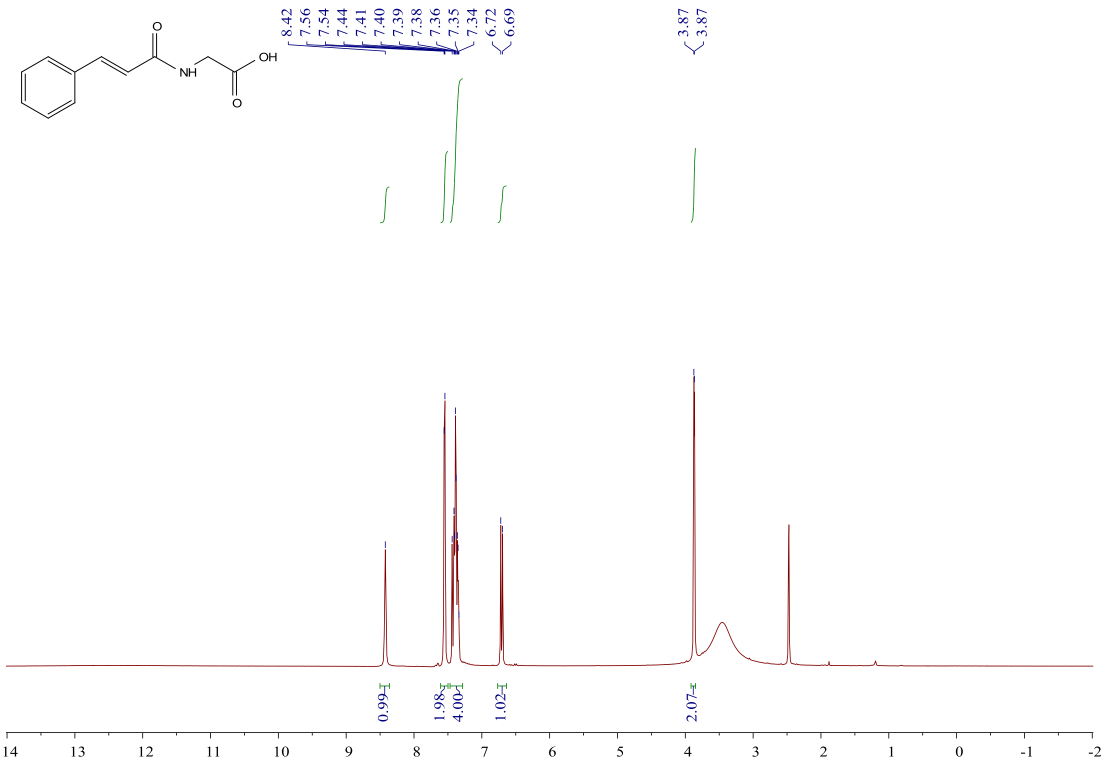

NH
OH
O
O
8.42
7.56
7.54
7.44
7.41
7.40
7.39
7.38
7.36
7.35
7.34
6.72
6.69
3.87
3.87
0.99
1.98
4.00
1.02
2.07
14 13 12 11 10 9 8 7 6 5 4 3 2 1 0 -1 -2

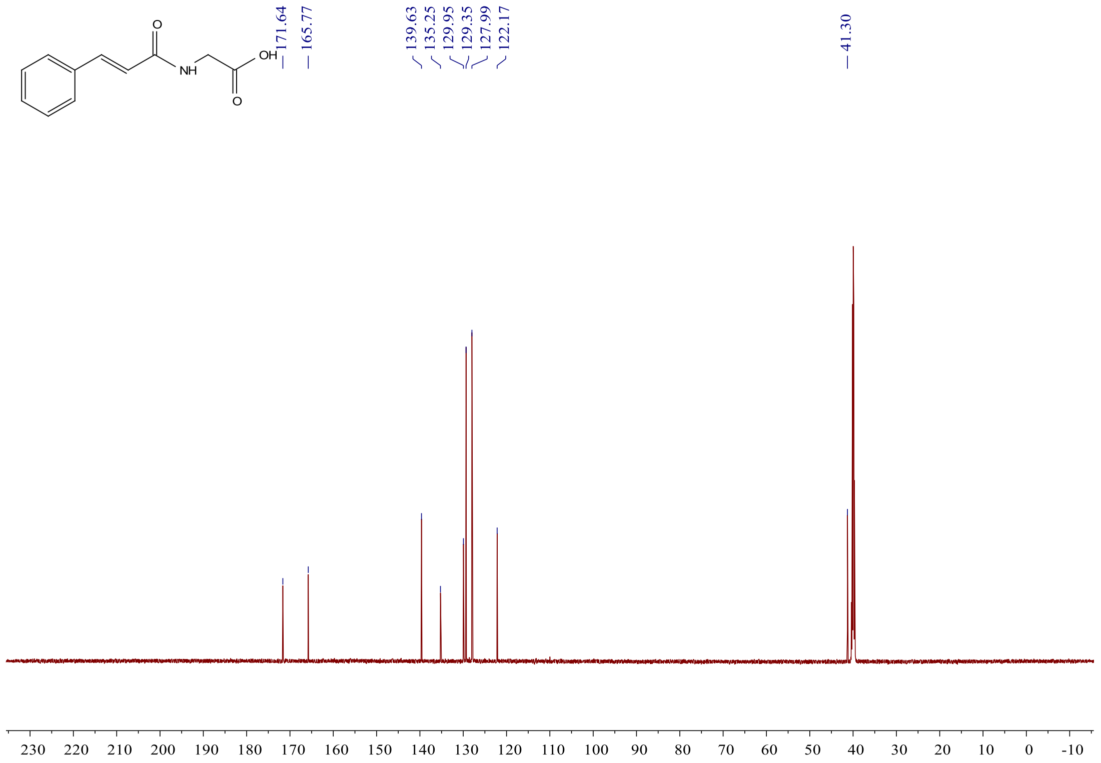

NH
OH
O
O
171.64
165.77
139.63
135.25
129.95
129.35
127.99
122.17
41.30
230 220 210 200 190 180 170 160 150 140 130 120 110 100 90 80 70 60 50 40 30 20 10 0 -10

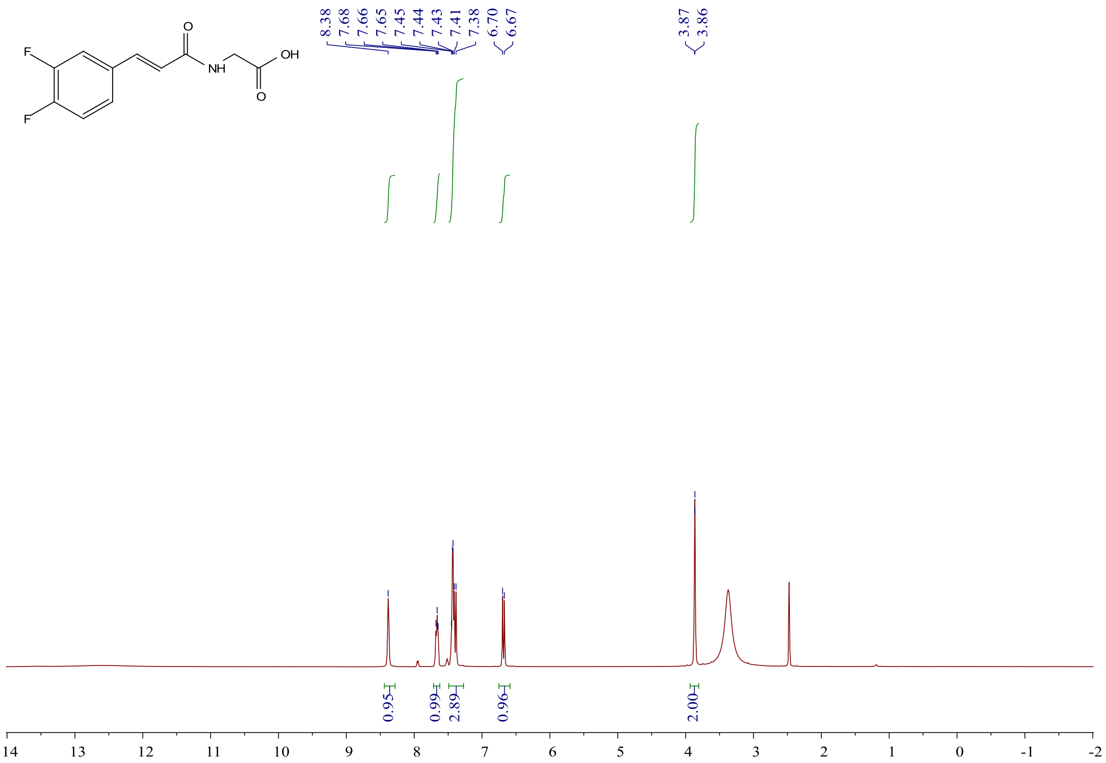
F
F
O
NH
OH
O
8.38
7.68
7.66
7.65
7.45
7.44
7.43
7.41
7.38
6.70
6.67
3.87
3.86
0.95
0.99
2.89
0.96
2.00
14 13 12 11 10 9 8 7 6 5 4 3 2 1 0 -1 -2

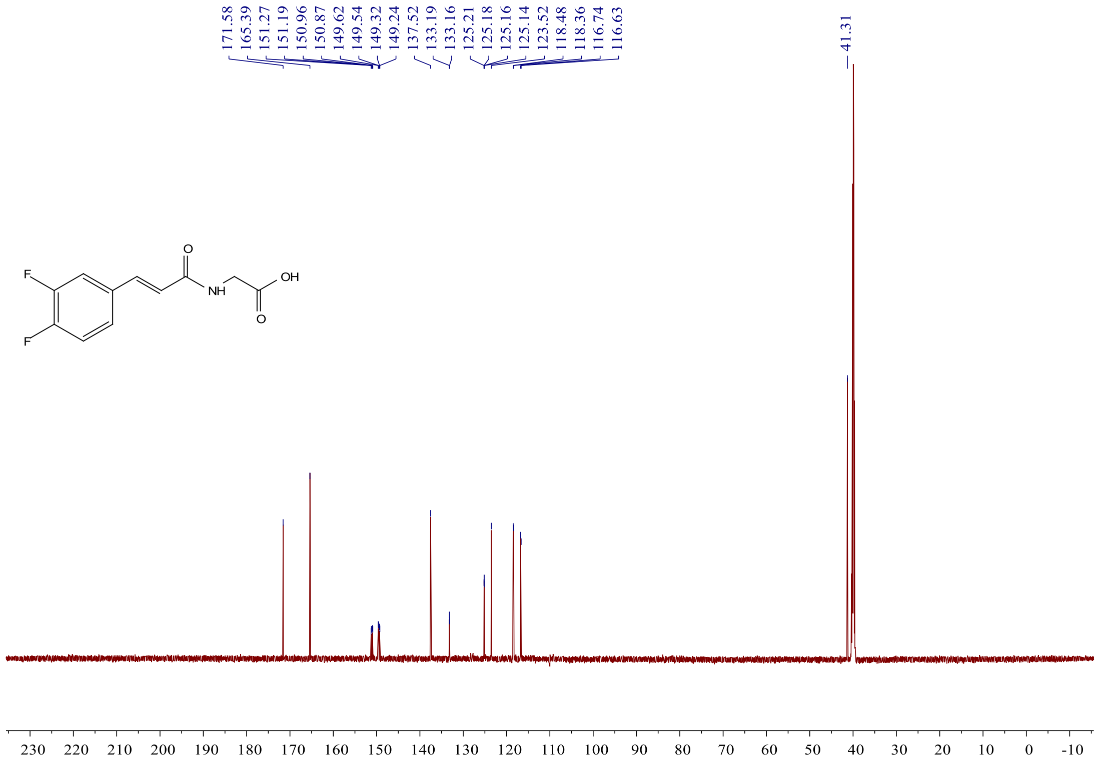
171.58
165.39
151.27
151.19
150.96
150.87
149.62
149.54
149.32
149.24
137.52
133.19
133.16
125.21
125.18
125.16
125.14
123.52
118.48
118.36
116.74
116.63
41.31
F
F
O
NH
OH
O
230 220 210 200 190 180 170 160 150 140 130 120 110 100 90 80 70 60 50 40 30 20 10 0 -10

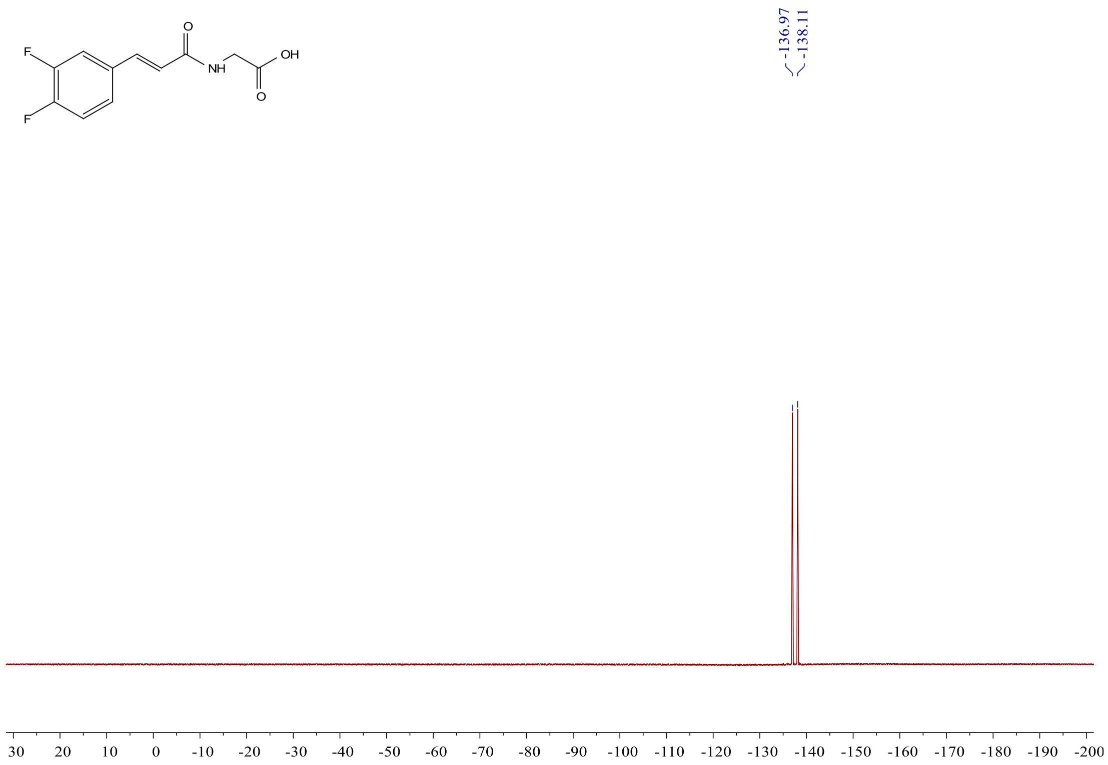
F
F
O
NH
OH
O
-136.97
-138.11
30 20 10 0 -10 -20 -30 -40 -50 -60 -70 -80 -90 -100 -110 -120 -130 -140 -150 -160 -170 -180 -190 -200

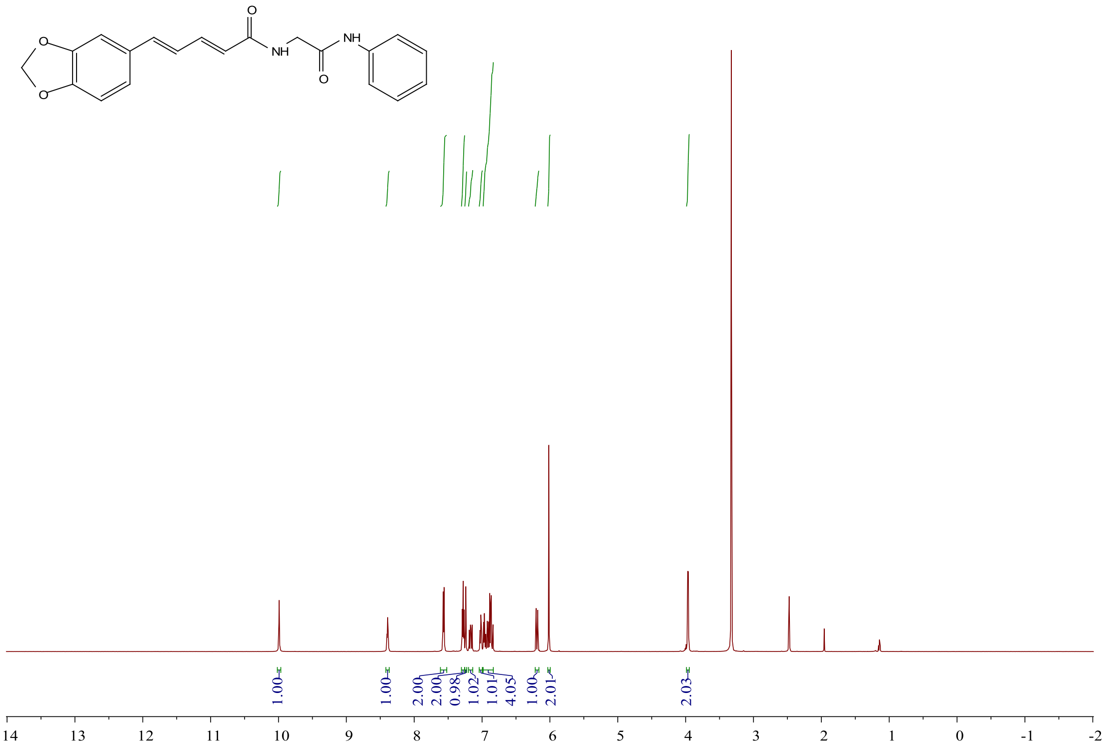
O
O
O
NH
NH
O
1.00
1.00
2.00
2.00
0.98
1.02
1.01
4.05
1.00
2.01
2.03
14 13 12 11 10 9 8 7 6 5 4 3 2 1 0 -1 -2

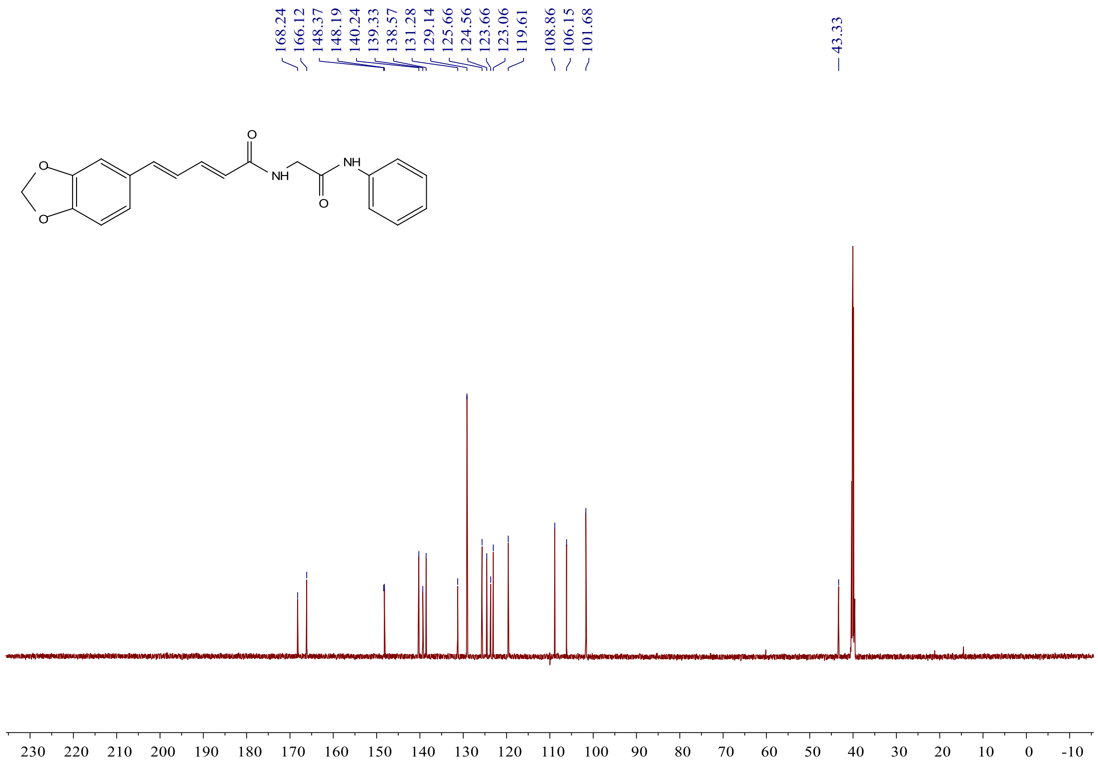
168.24
166.12
148.37
148.19
140.24
139.33
138.57
131.28
129.14
125.66
124.56
123.66
123.06
119.61
108.86
106.15
101.68
43.33
O
O
O
NH
NH
O
230 220 210 200 190 180 170 160 150 140 130 120 110 100 90 80 70 60 50 40 30 20 10 0 -10

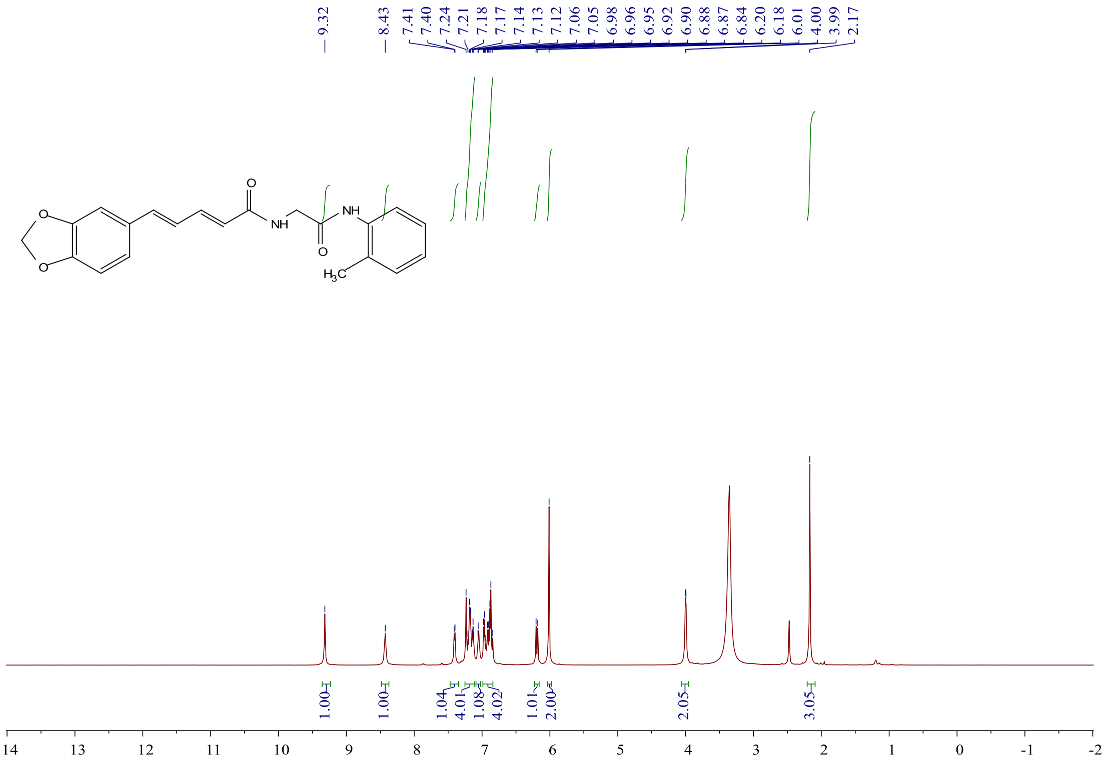
9.32
8.43
7.41
7.40
7.24
7.21
7.18
7.17
7.14
7.13
7.12
7.06
7.05
6.98
6.96
6.95
6.92
6.90
6.88
6.87
6.84
6.20
6.18
6.01
4.00
3.99
2.17
O
O
O
NH
NH
O
H3C
1.00
1.00
1.04
4.01
1.08
4.02
1.01
2.00
2.05
3.05
14 13 12 11 10 9 8 7 6 5 4 3 2 1 0 -1 -2

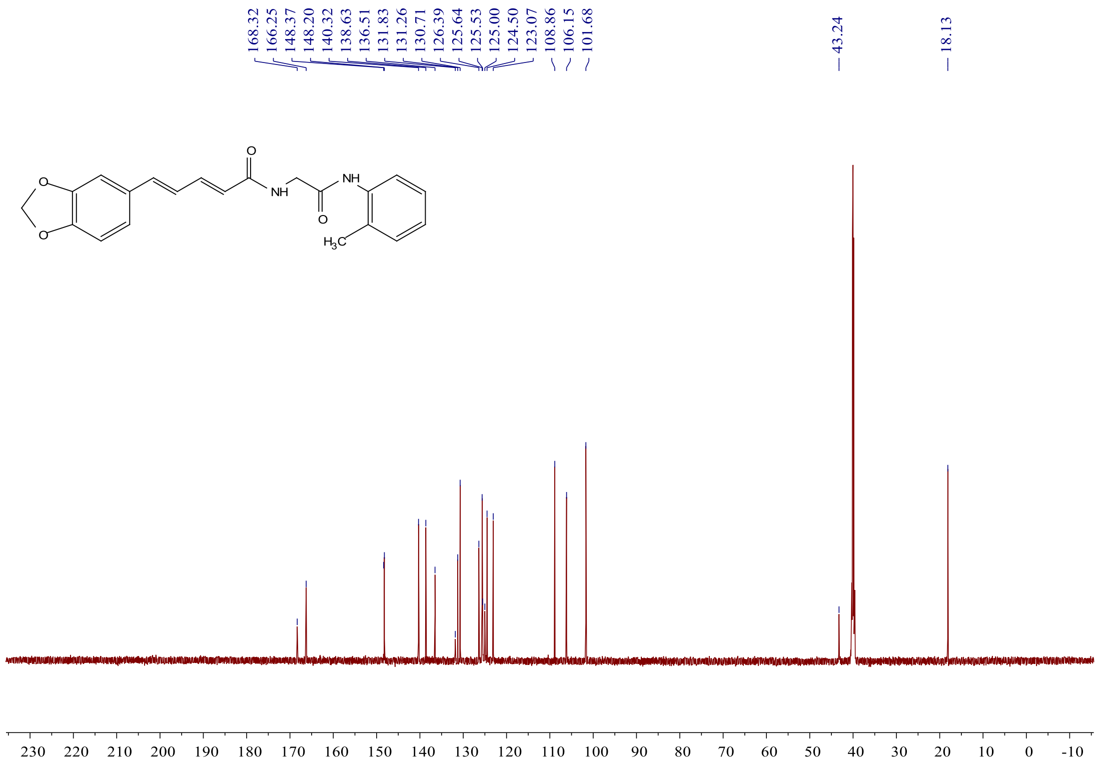

168.32
166.25
148.37
148.20
140.32
138.63
136.51
131.83
131.26
130.71
126.39
125.64
125.53
125.00
124.50
123.07
108.86
106.15
101.68
43.24
18.13
O
O
O
NH
NH
O
H3C
230 220 210 200 190 180 170 160 150 140 130 120 110 100 90 80 70 60 50 40 30 20 10 0 -10

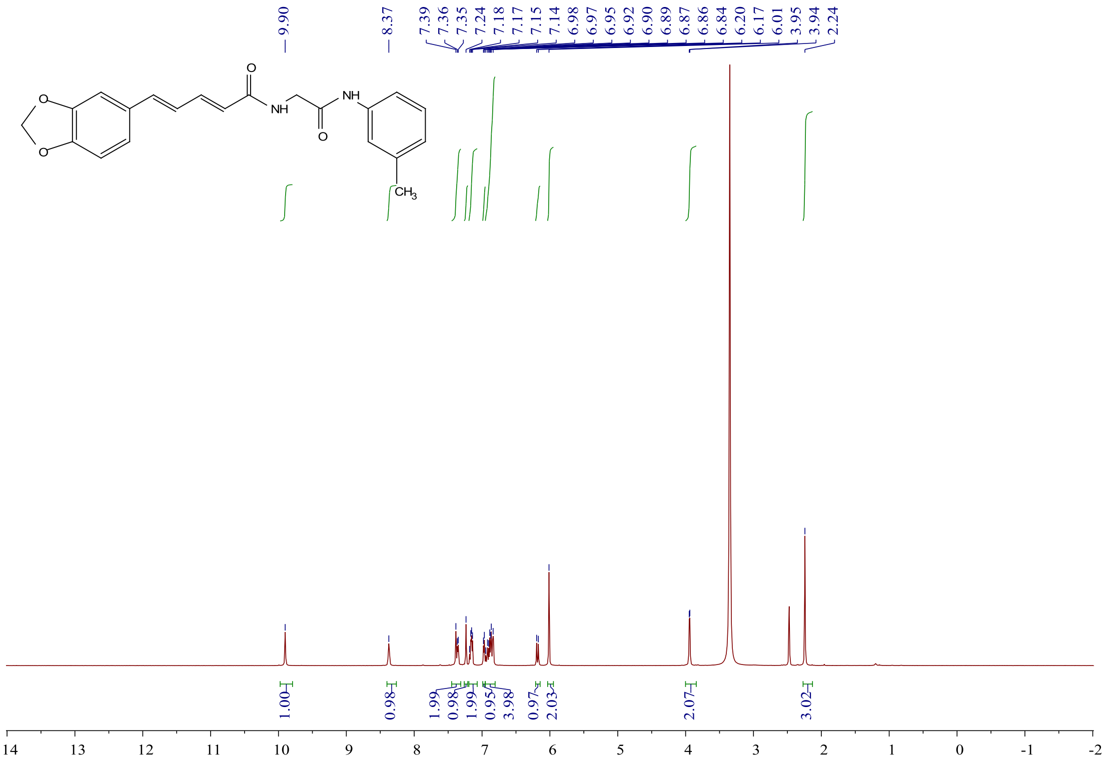

9.90
8.37
7.39
7.36
7.35
7.24
7.18
7.17
7.15
7.14
6.98
6.97
6.95
6.92
6.90
6.89
6.87
6.86
6.84
6.20
6.17
6.01
3.95
3.94
2.24
O
O
O
NH
NH
O
CH3
1.00
0.98
1.99
0.98
1.99
0.95
3.98
0.97
2.03
2.07
3.02
14 13 12 11 10 9 8 7 6 5 4 3 2 1 0 -1 -2

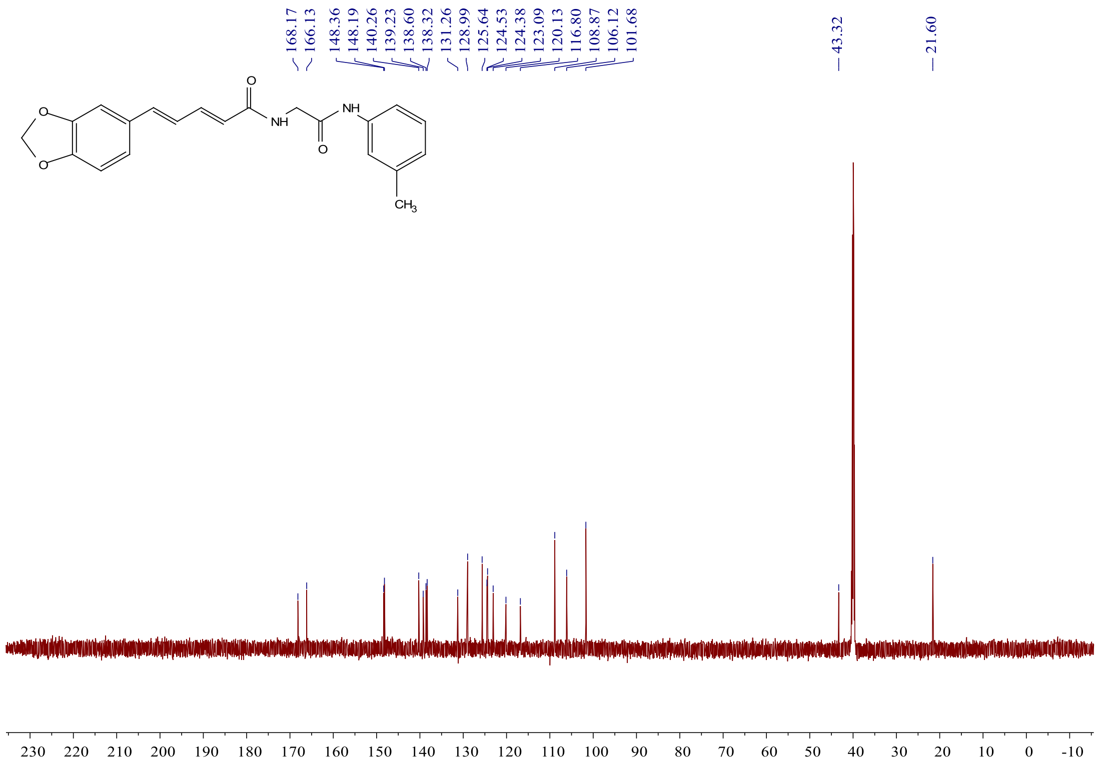
168.17
166.13
148.36
148.19
140.26
139.23
138.60
138.32
131.26
128.99
125.64
124.53
124.38
123.09
120.13
116.80
108.87
106.12
101.68
43.32
21.60
O
NH
NH
O
O
O
CH3

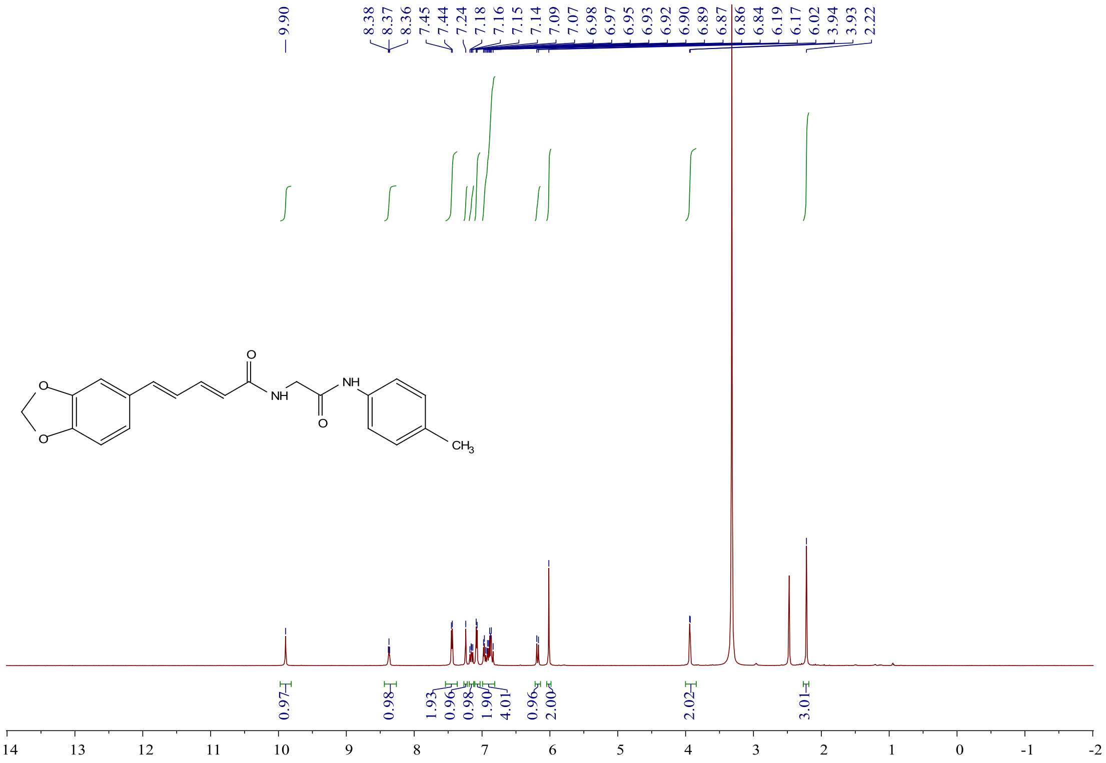
9.90
8.38
8.37
8.36
7.45
7.44
7.24
7.18
7.16
7.15
7.14
7.09
7.07
6.98
6.97
6.95
6.93
6.92
6.90
6.89
6.87
6.86
6.84
6.19
6.17
6.02
3.94
3.93
2.22
O
NH
NH
O
O
O
CH3
0.97
0.98
1.93
0.96
0.98
1.90
4.01
0.96
2.00
2.02
3.01

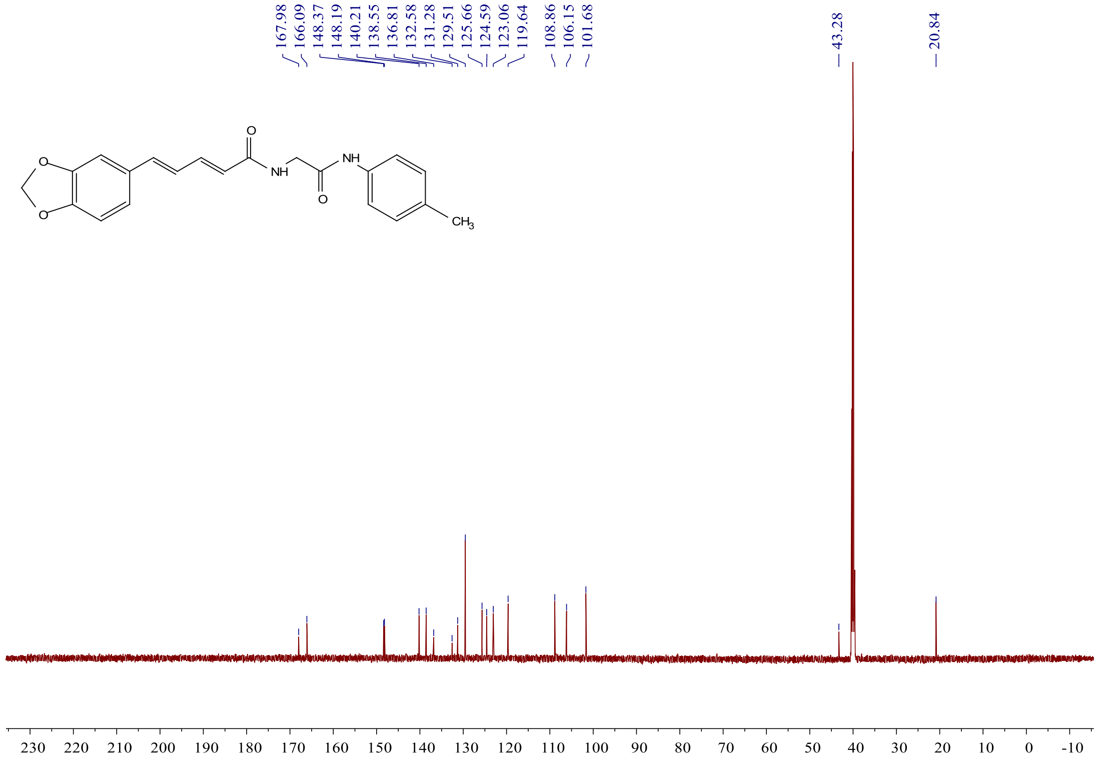

167.98
166.09
148.37
148.19
140.21
138.55
136.81
132.58
131.28
129.51
125.66
124.59
123.06
119.64
108.86
106.15
101.68
43.28
20.84
O
NH
CH3
230 220 210 200 190 180 170 160 150 140 130 120 110 100 90 80 70 60 50 40 30 20 10 0 -10

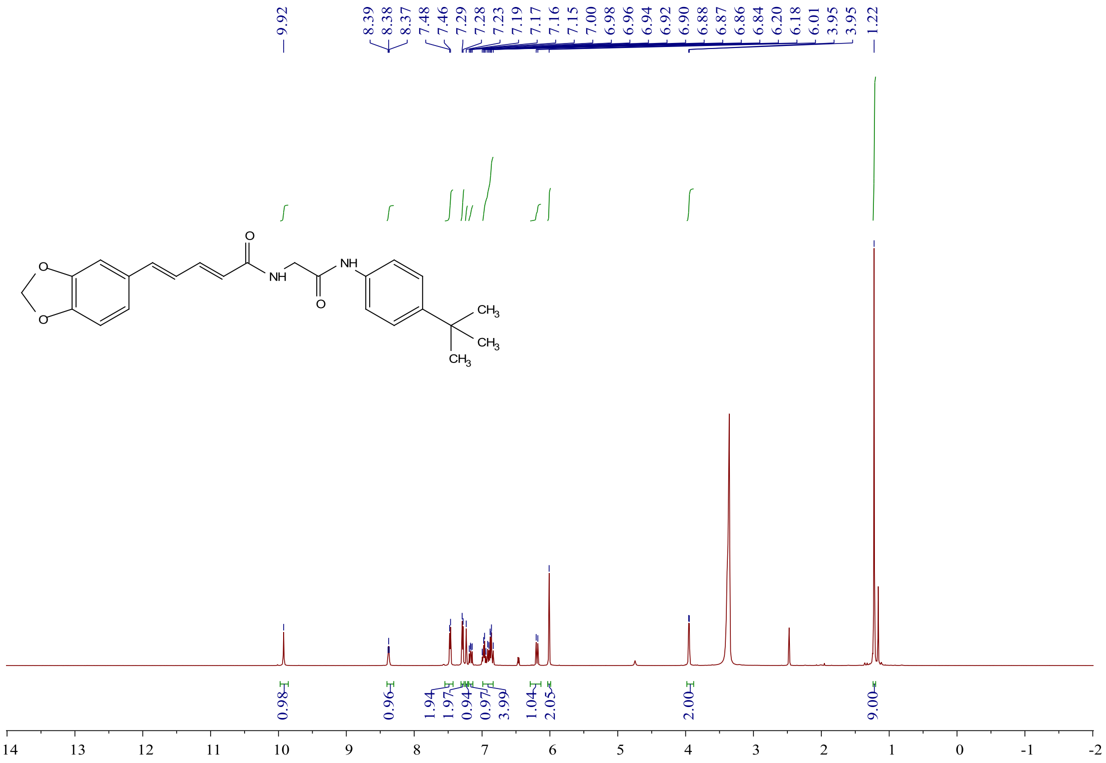

9.92
8.39
8.38
8.37
7.48
7.46
7.29
7.28
7.23
7.19
7.17
7.16
7.15
7.00
6.98
6.96
6.94
6.92
6.90
6.88
6.87
6.86
6.84
6.20
6.18
6.01
3.95
3.95
1.22
O
NH
CH3
0.98
0.96
1.94
1.97
0.94
0.97
3.99
1.04
2.05
2.00
9.00
14 13 12 11 10 9 8 7 6 5 4 3 2 1 0 -1 -2

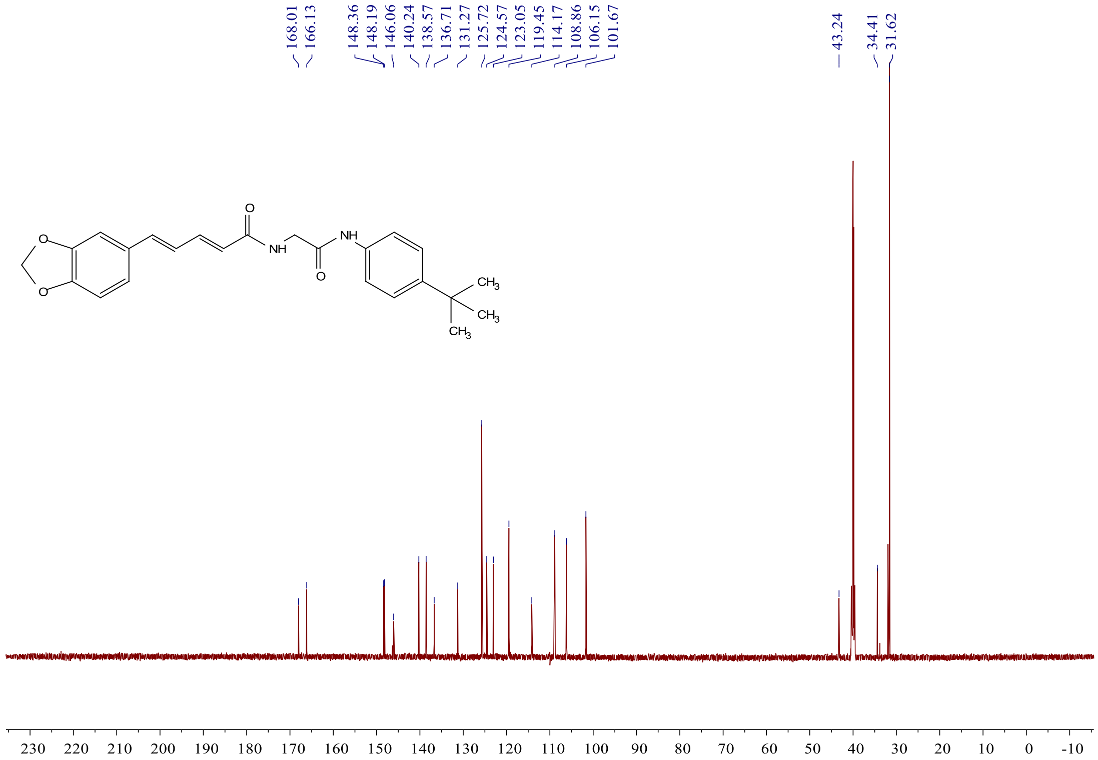
168.01
166.13
148.36
148.19
146.06
140.24
138.57
136.71
131.27
125.72
124.57
123.05
119.45
114.17
108.86
106.15
101.67
43.24
34.41
31.62
O
NH
NH
O
CH3
CH3
CH3
230 220 210 200 190 180 170 160 150 140 130 120 110 100 90 80 70 60 50 40 30 20 10 0 -10

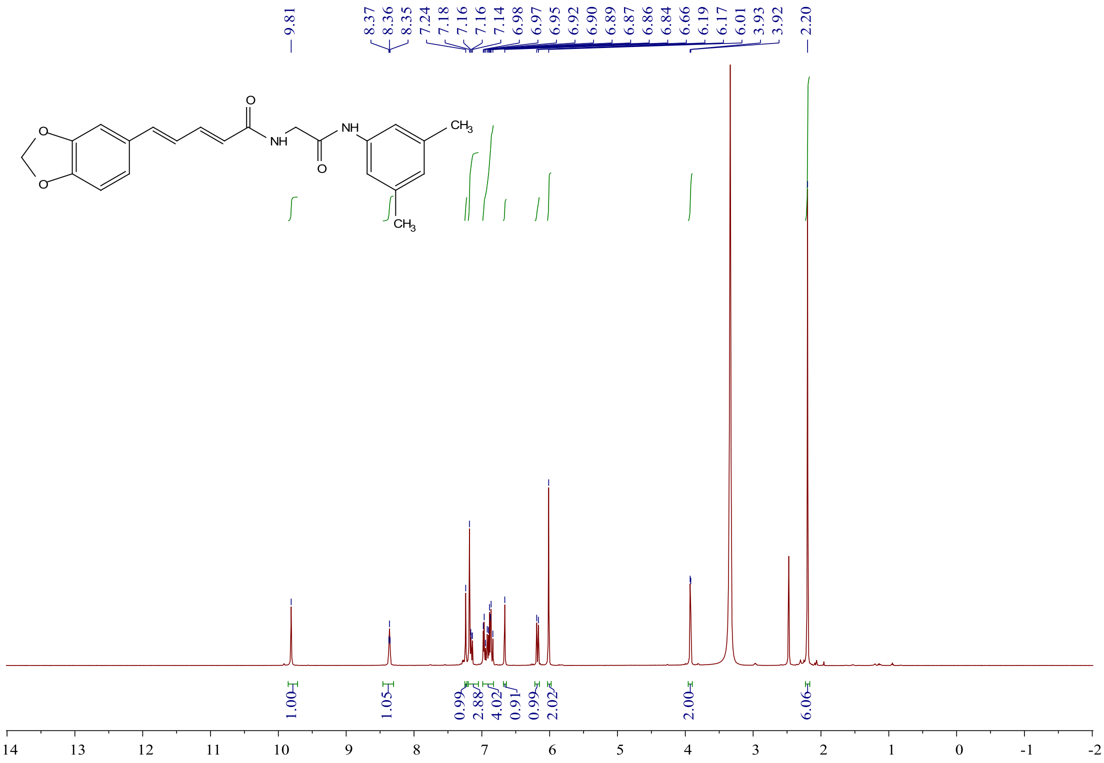
9.81
8.37
8.36
8.35
7.24
7.18
7.16
7.16
7.14
6.98
6.97
6.95
6.92
6.90
6.89
6.87
6.86
6.84
6.66
6.19
6.17
6.01
3.93
3.92
2.20
O
NH
NH
O
CH3
CH3
1.00
1.05
0.99
2.88
4.02
0.91
0.99
2.02
2.00
6.06
14 13 12 11 10 9 8 7 6 5 4 3 2 1 0 -1 -2

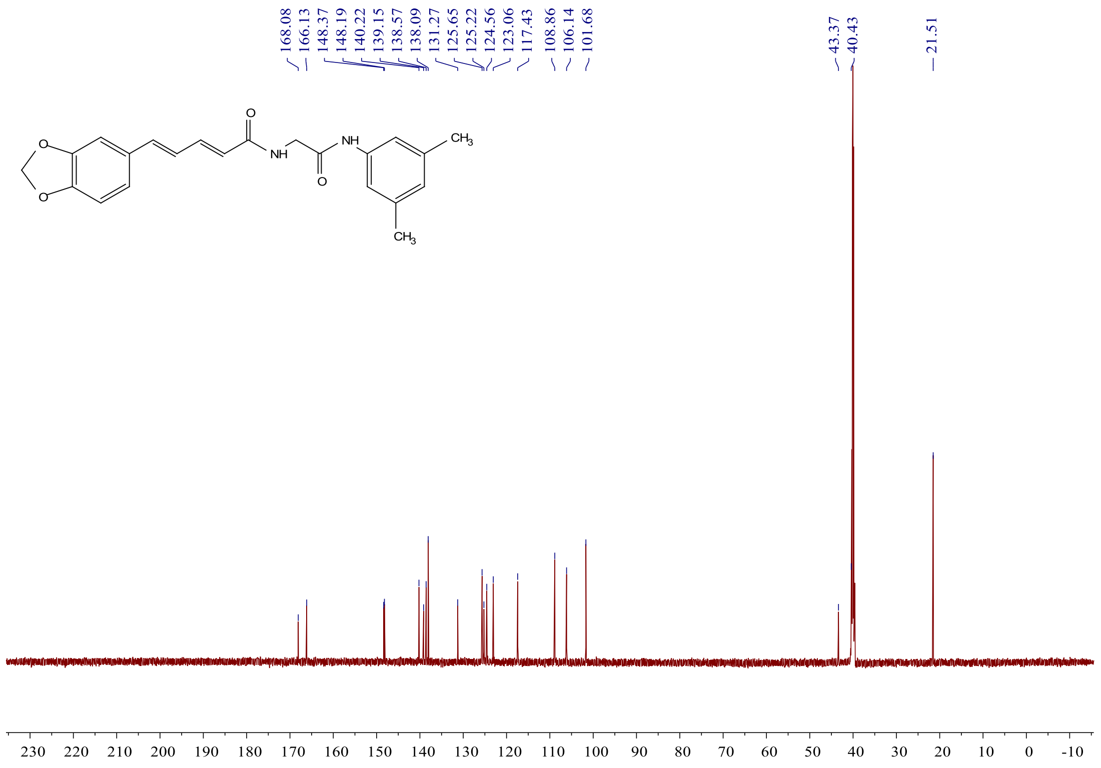

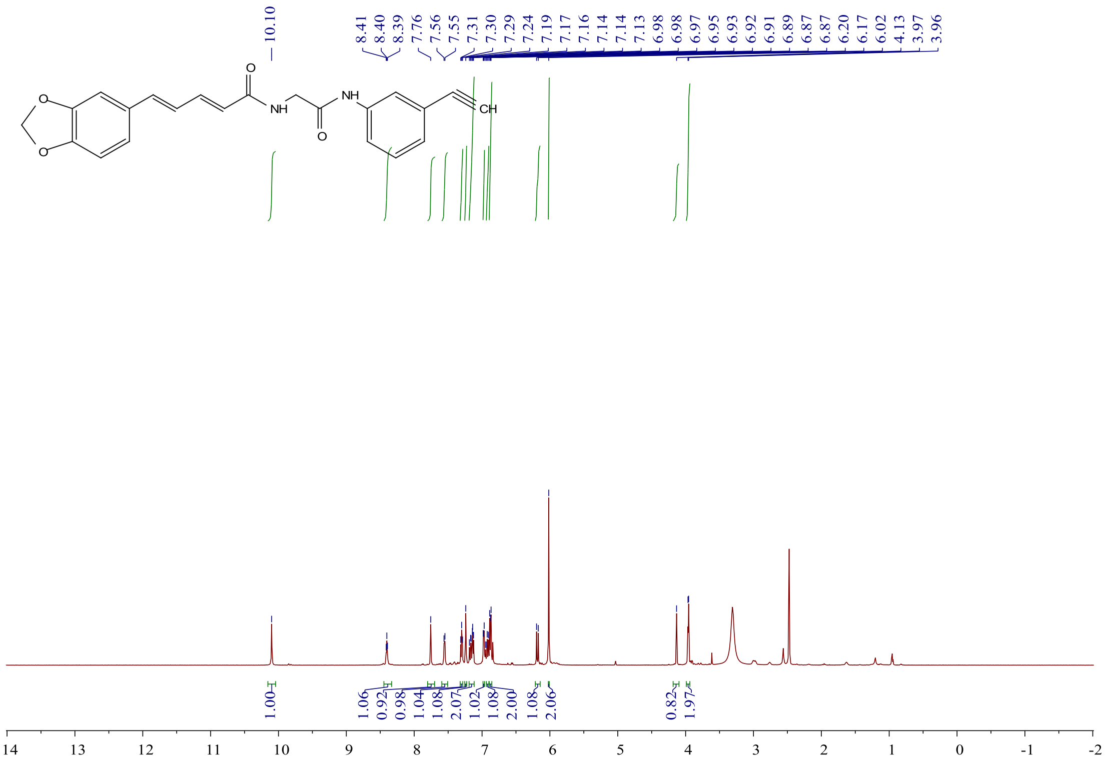

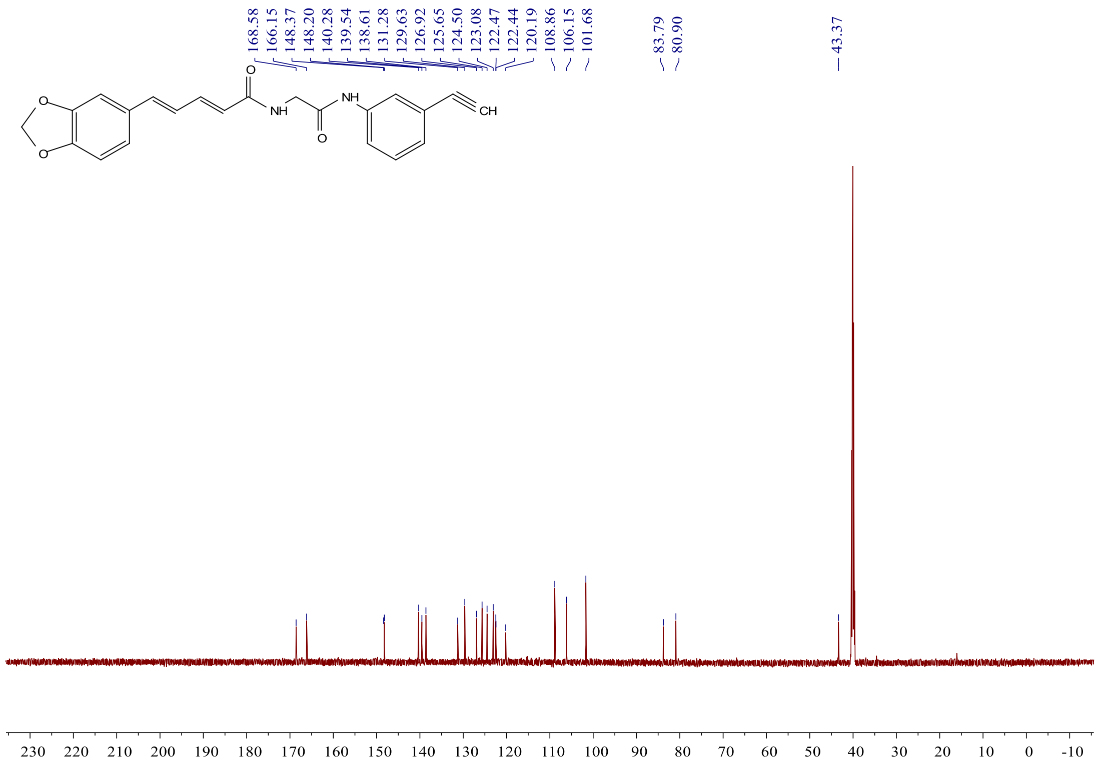
168.58
166.15
148.37
148.20
140.28
139.54
138.61
131.28
129.63
126.92
125.65
124.50
123.08
122.47
122.44
120.19
108.86
106.15
101.68
83.79
80.90
43.37
O
O
O
NH
NH
O
CH
230 220 210 200 190 180 170 160 150 140 130 120 110 100 90 80 70 60 50 40 30 20 10 0 -10

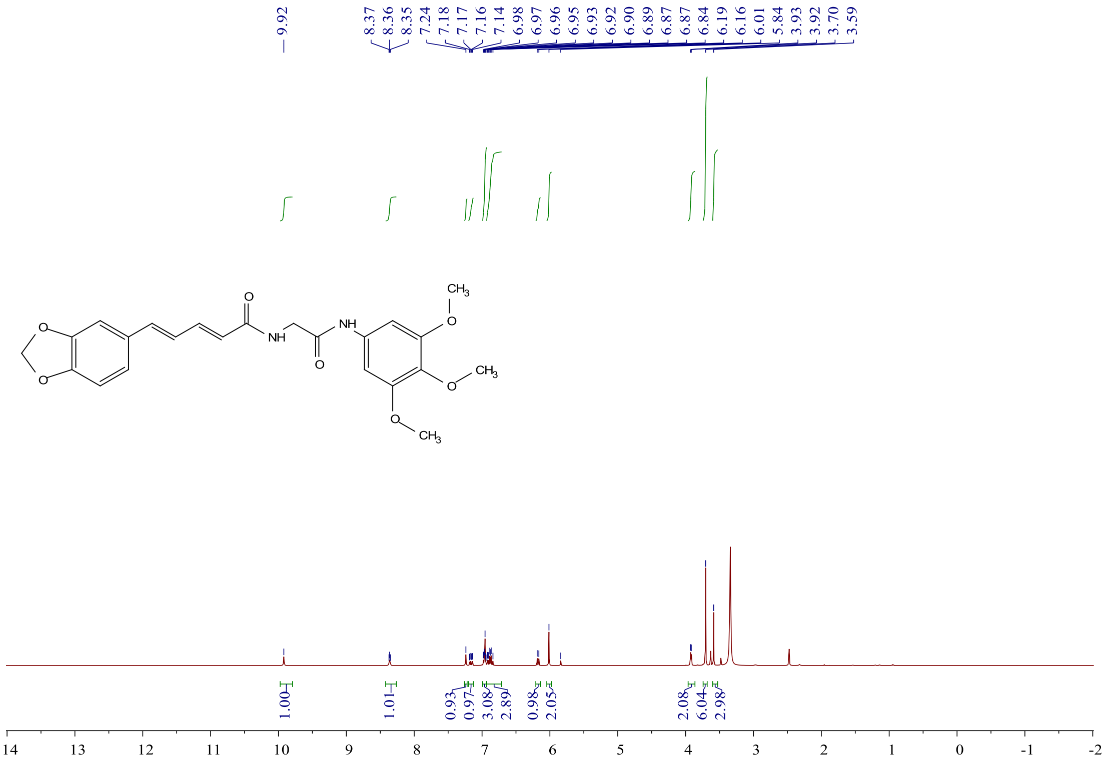
9.92
8.37
8.36
8.35
7.24
7.18
7.17
7.16
7.14
6.98
6.97
6.96
6.95
6.93
6.92
6.90
6.89
6.87
6.87
6.84
6.19
6.16
6.01
5.84
3.93
3.92
3.70
3.59
O
O
O
NH
NH
O
CH_3
O
O
CH_3
O
CH_3
1.00
1.01
0.93
0.97
3.08
2.89
0.98
2.05
2.08
6.04
2.98
14 13 12 11 10 9 8 7 6 5 4 3 2 1 0 -1 -2

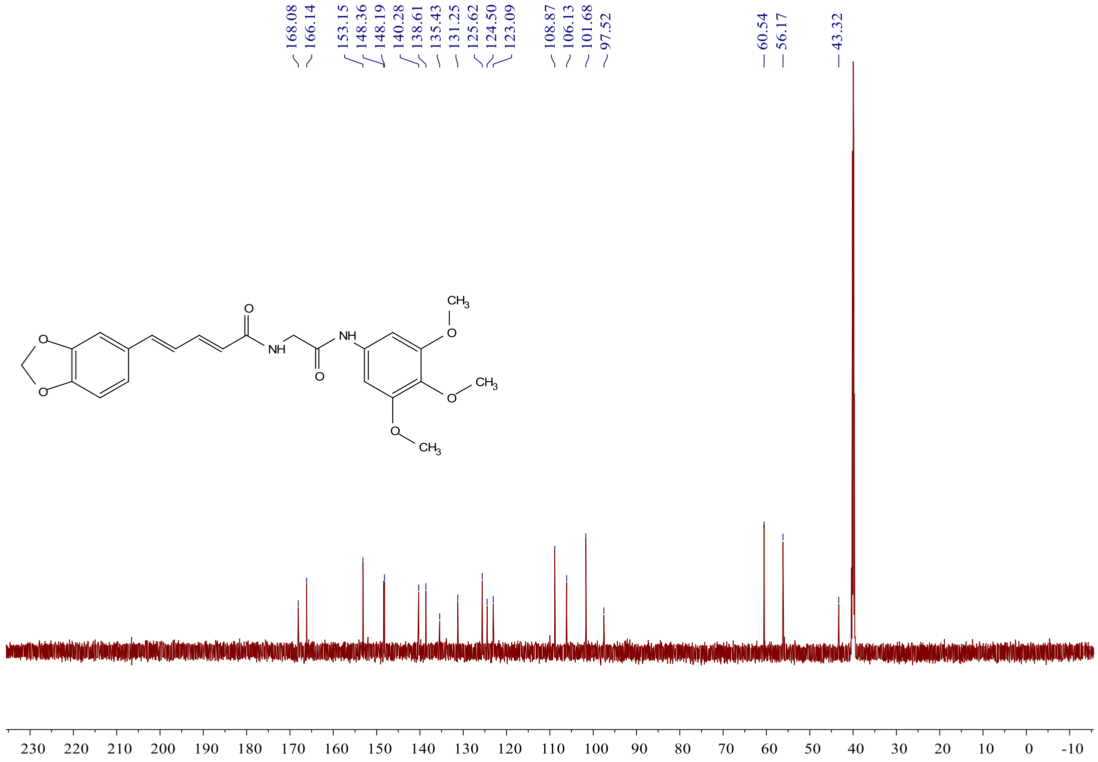
168.08
166.14
153.15
148.36
148.19
140.28
138.61
135.43
131.25
125.62
124.50
123.09
108.87
106.13
101.68
97.52
60.54
56.17
43.32
CH3
O
NH
230 220 210 200 190 180 170 160 150 140 130 120 110 100 90 80 70 60 50 40 30 20 10 0 -10

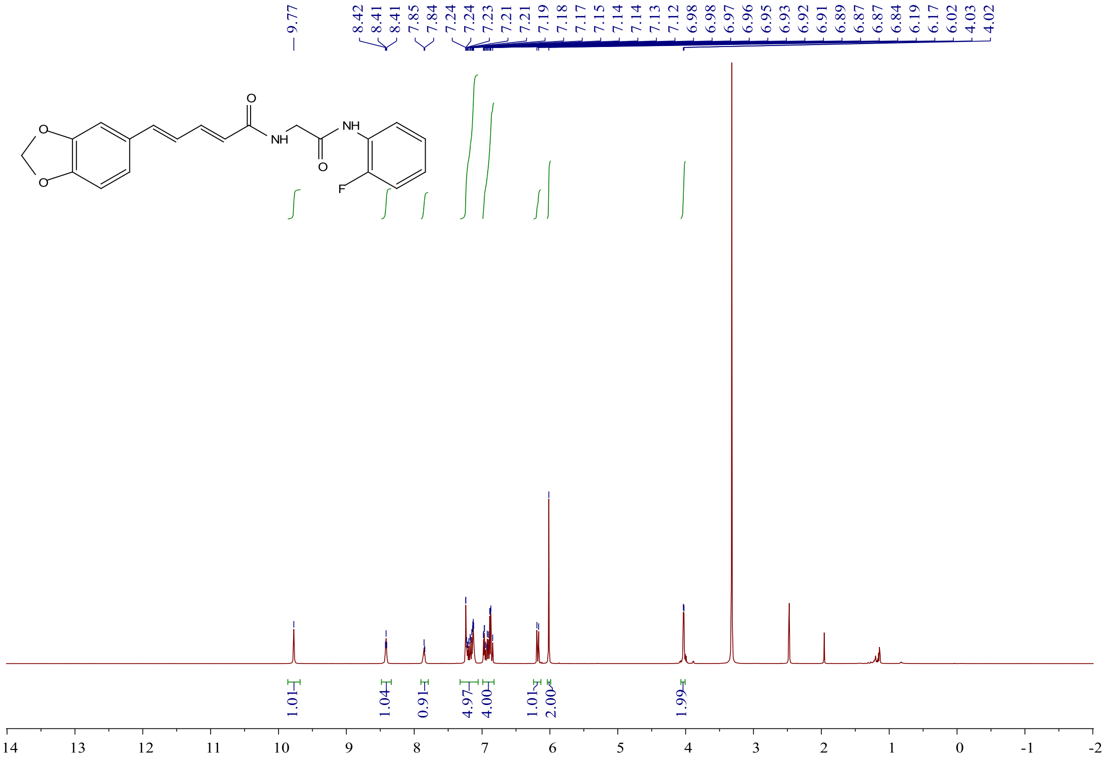
9.77
8.42
8.41
8.41
7.85
7.84
7.24
7.24
7.23
7.21
7.21
7.19
7.18
7.17
7.15
7.14
7.14
7.13
7.12
6.98
6.98
6.97
6.96
6.95
6.93
6.92
6.91
6.89
6.87
6.87
6.84
6.19
6.17
6.02
4.03
4.02
O
NH
F
1.01
1.04
0.91
4.97
4.00
1.01
2.00
1.99
14 13 12 11 10 9 8 7 6 5 4 3 2 1 0 -1 -2

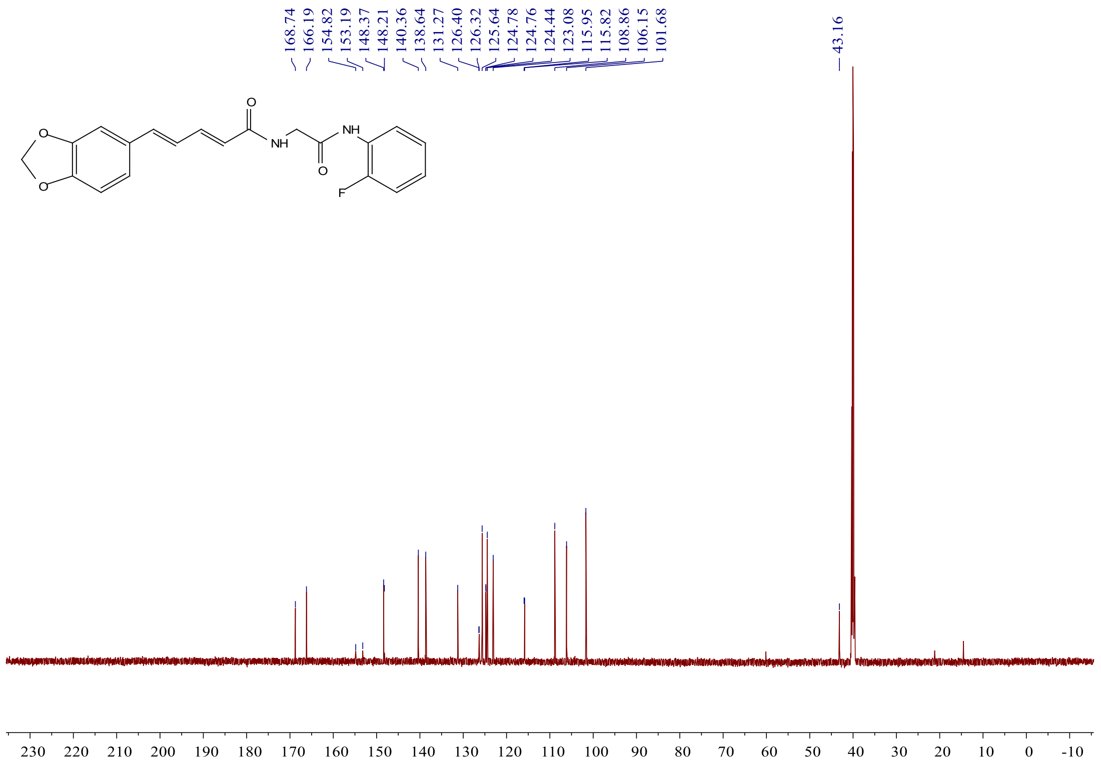
168.74
166.19
154.82
153.19
148.37
148.21
140.36
138.64
131.27
126.40
126.32
125.64
124.78
124.76
124.44
123.08
115.95
115.82
108.86
106.15
101.68
43.16
O
NH
NH
O
O
O
F
230 220 210 200 190 180 170 160 150 140 130 120 110 100 90 80 70 60 50 40 30 20 10 0 -10

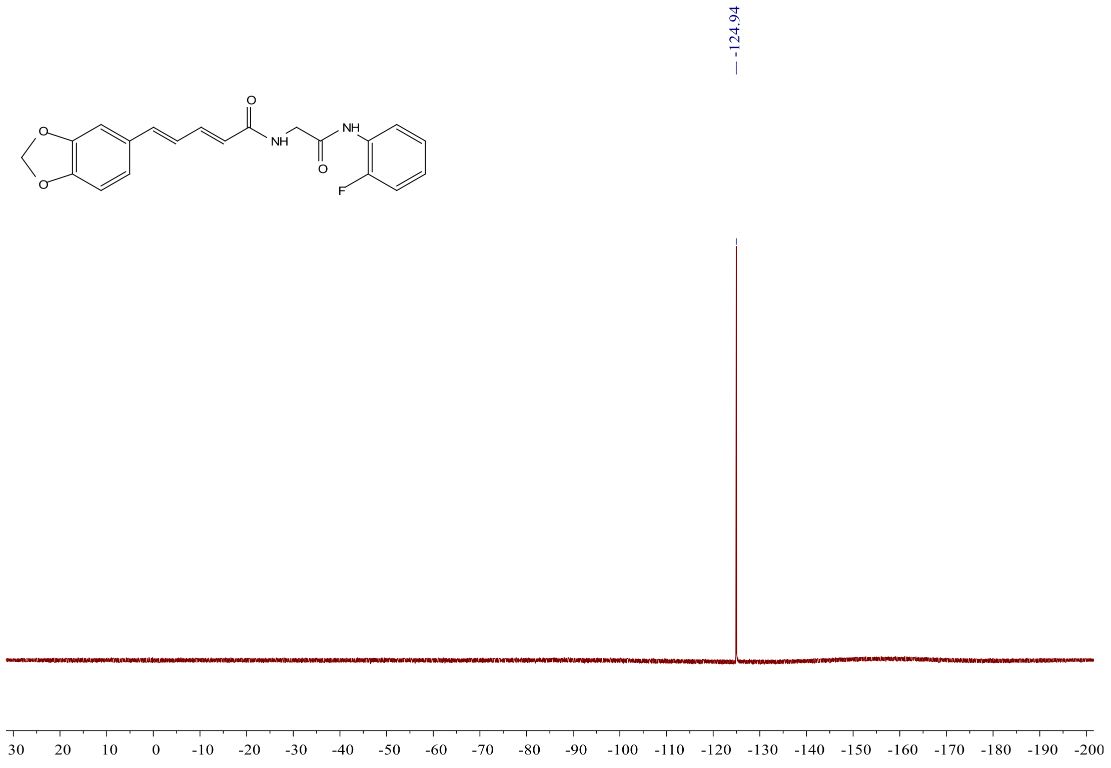
-124.94
O
NH
NH
O
O
O
F
30 20 10 0 -10 -20 -30 -40 -50 -60 -70 -80 -90 -100 -110 -120 -130 -140 -150 -160 -170 -180 -190 -200

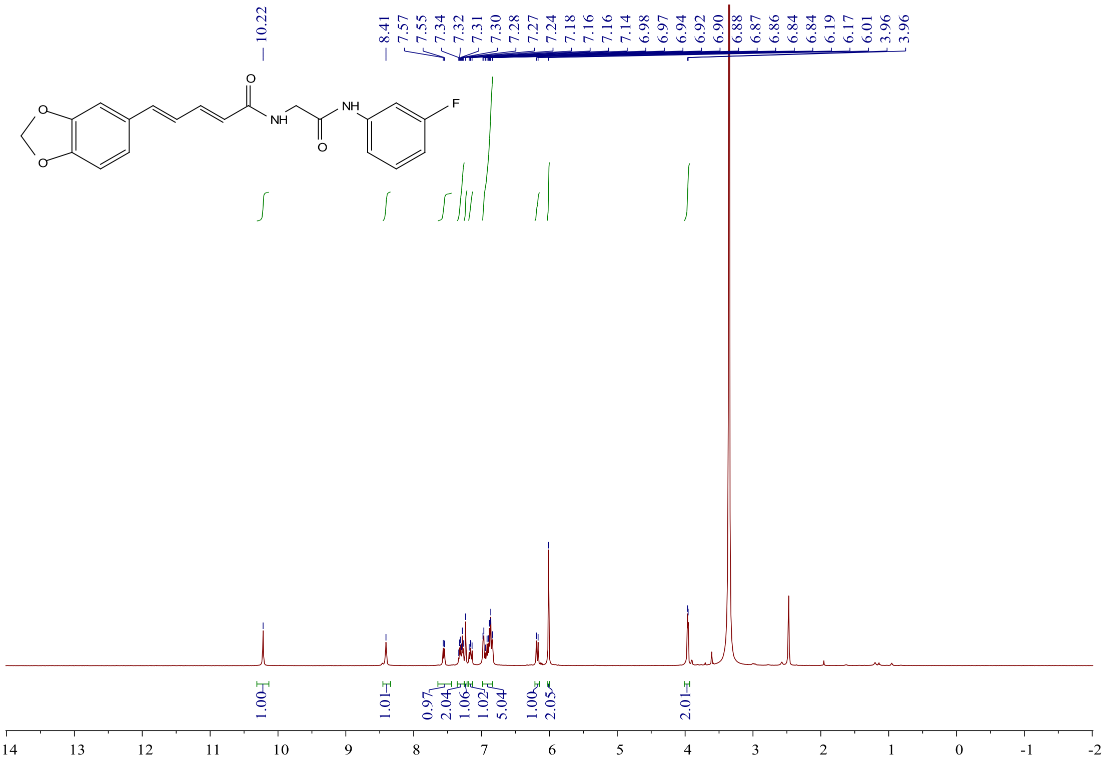
10.22
8.41
7.57
7.55
7.34
7.32
7.31
7.30
7.28
7.27
7.24
7.18
7.16
7.16
7.14
6.98
6.97
6.94
6.92
6.90
6.88
6.87
6.86
6.84
6.84
6.19
6.17
6.01
3.96
3.96
O
NH
NH
O
O
O
F
1.00
1.01
0.97
2.04
1.06
1.02
5.04
1.00
2.05
2.01
14 13 12 11 10 9 8 7 6 5 4 3 2 1 0 -1 -2

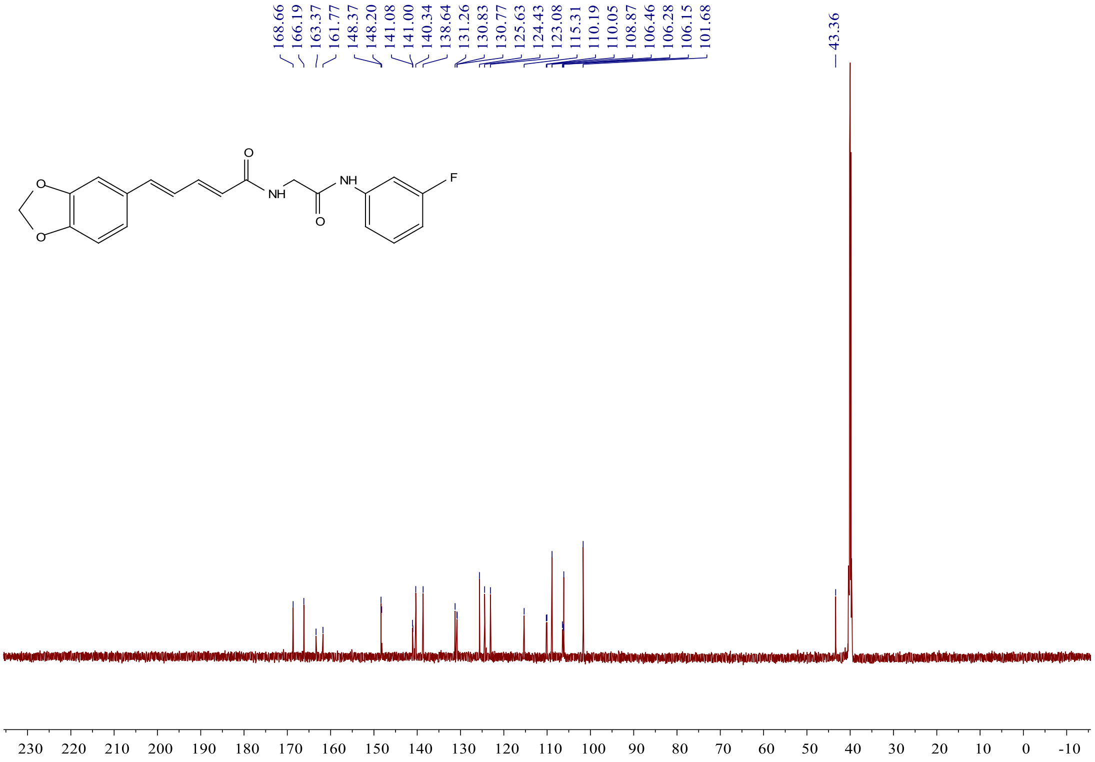
168.66
166.19
163.37
161.77
148.37
148.20
141.08
141.00
140.34
138.64
131.26
130.83
130.77
125.63
124.43
123.08
115.31
110.19
110.05
108.87
106.46
106.28
106.15
101.68
43.36
O
NH
NH
O
O
O
F
230 220 210 200 190 180 170 160 150 140 130 120 110 100 90 80 70 60 50 40 30 20 10 0 -10

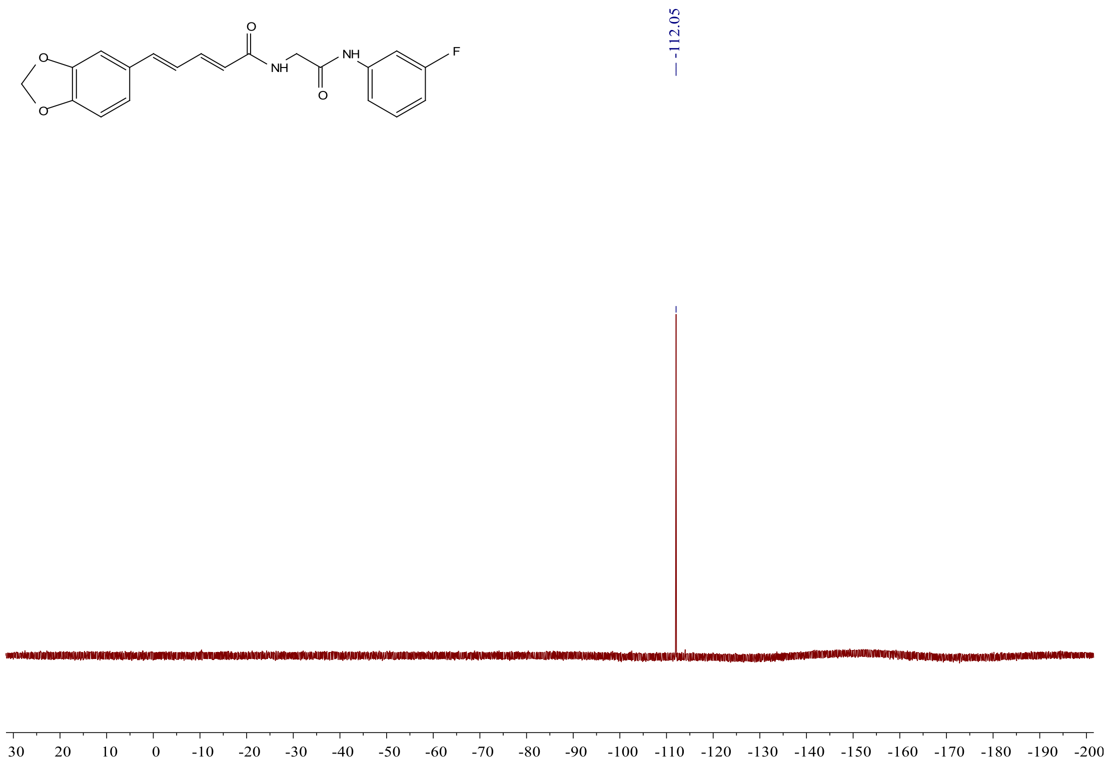
O
NH
NH
O
F
O
O
-112.05
30 20 10 0 -10 -20 -30 -40 -50 -60 -70 -80 -90 -100 -110 -120 -130 -140 -150 -160 -170 -180 -190 -200

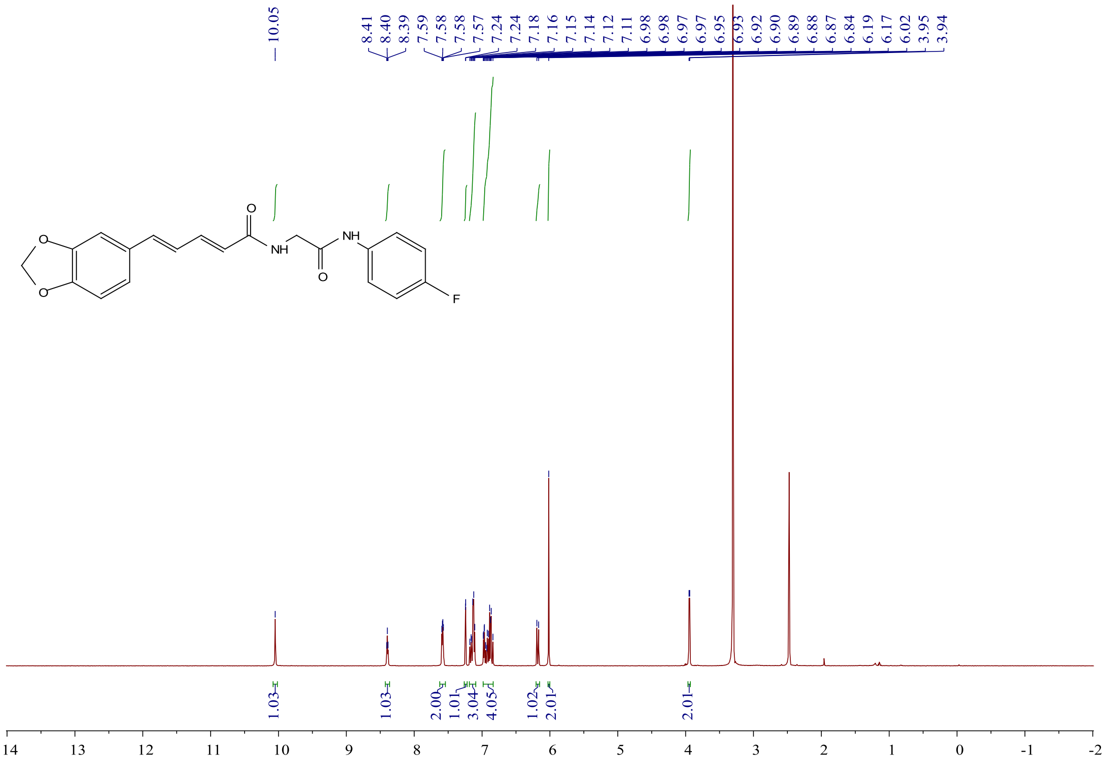
10.05
8.41 8.40 8.39 7.59 7.58 7.58 7.57 7.24 7.24 7.18 7.16 7.15 7.14 7.12 7.11 6.98 6.98 6.97 6.95 6.93 6.92 6.90 6.89 6.88 6.87 6.84 6.19 6.17 6.02 3.95 3.94
O
NH
NH
O
F
O
O
1.03
1.03
2.00
1.01
3.04
4.05
1.02
2.01
2.01
14 13 12 11 10 9 8 7 6 5 4 3 2 1 0 -1 -2

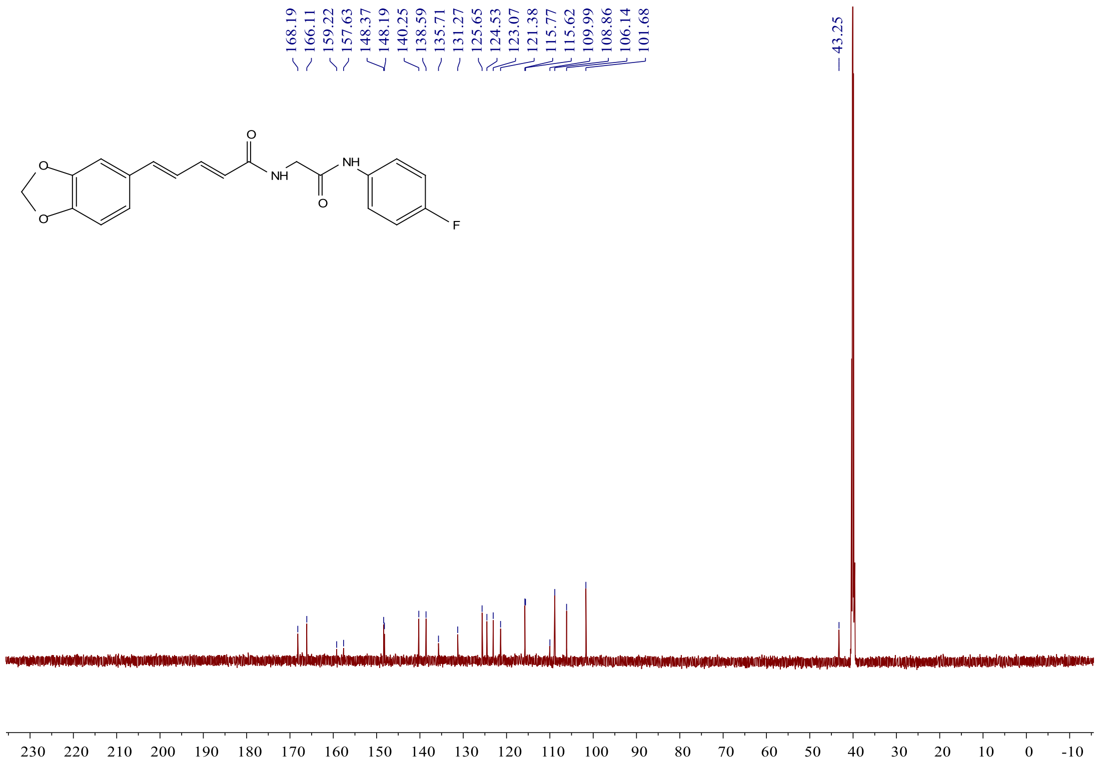
168.19
166.11
159.22
157.63
148.37
148.19
140.25
138.59
135.71
131.27
125.65
124.53
123.07
121.38
115.77
115.62
109.99
108.86
106.14
101.68
43.25
O
O
O
NH
NH
O
F
230 220 210 200 190 180 170 160 150 140 130 120 110 100 90 80 70 60 50 40 30 20 10 0 -10

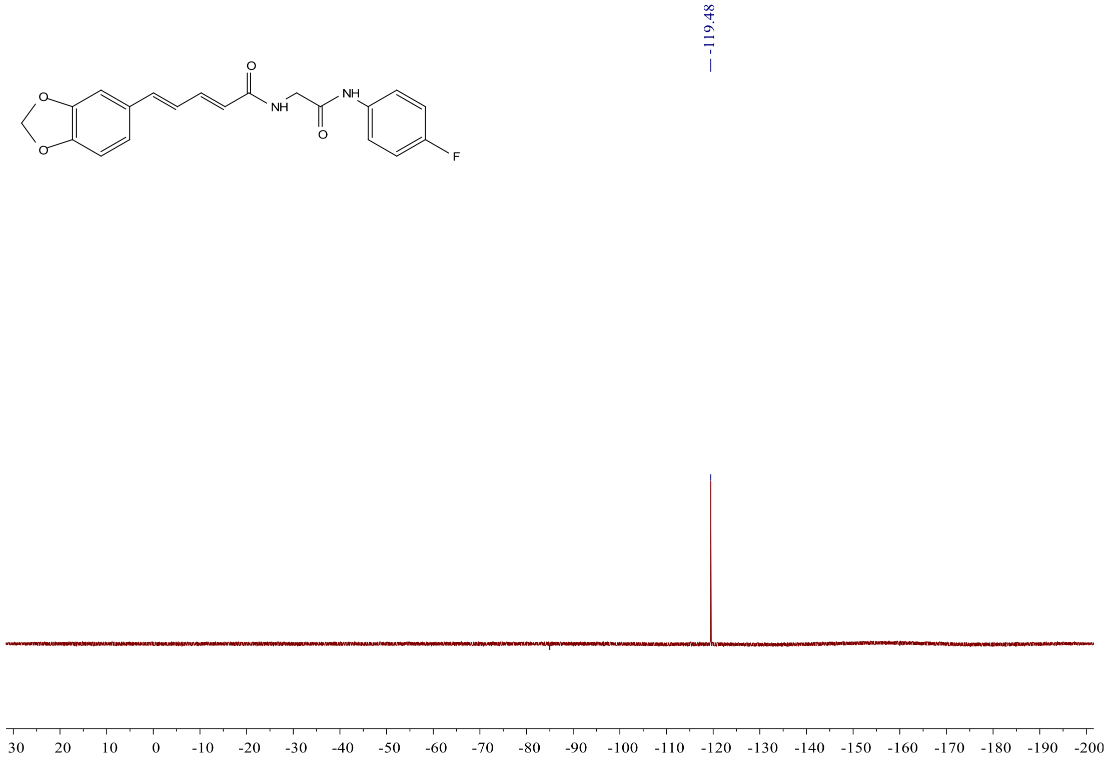
-119.48
O
O
O
NH
NH
O
F
30 20 10 0 -10 -20 -30 -40 -50 -60 -70 -80 -90 -100 -110 -120 -130 -140 -150 -160 -170 -180 -190 -200

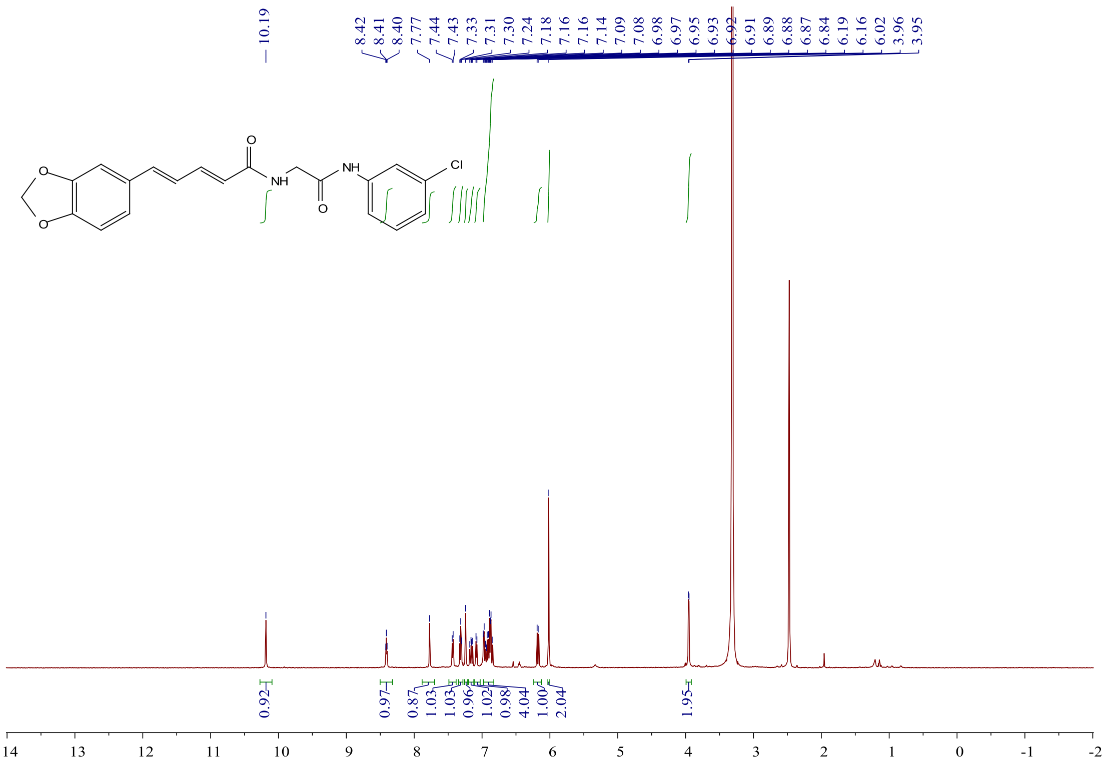
10.19
8.42
8.41
8.40
7.77
7.44
7.43
7.33
7.31
7.30
7.24
7.18
7.16
7.16
7.14
7.09
7.08
6.98
6.97
6.95
6.93
6.92
6.91
6.89
6.88
6.87
6.84
6.19
6.16
6.02
3.96
3.95
O
O
O
NH
O
NH
Cl
0.92
0.97
0.87
1.03
1.03
0.96
1.02
0.98
4.04
1.00
2.04
1.95
14 13 12 11 10 9 8 7 6 5 4 3 2 1 0 -1 -2

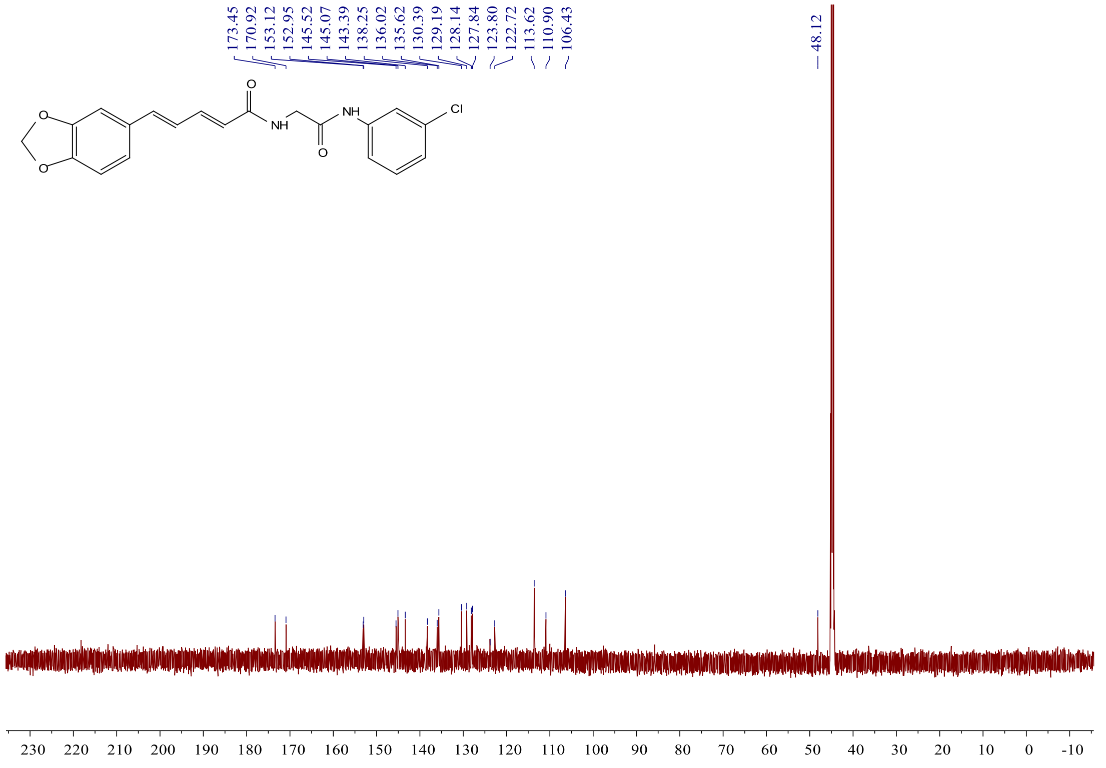
173.45
170.92
153.12
152.95
145.52
145.07
143.39
138.25
136.02
135.62
130.39
129.19
128.14
127.84
123.80
122.72
113.62
110.90
106.43
48.12
O
O
O
NH
O
NH
Cl
230 220 210 200 190 180 170 160 150 140 130 120 110 100 90 80 70 60 50 40 30 20 10 0 -10

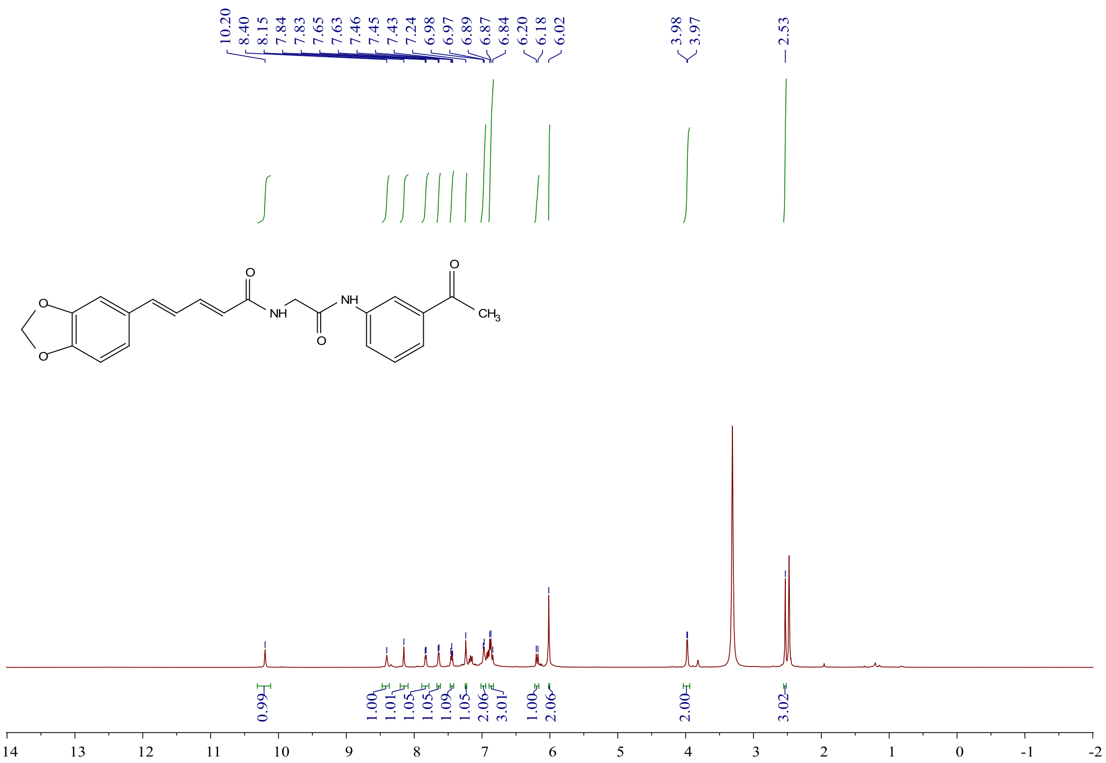

10.20
8.40
8.15
7.84
7.83
7.65
7.63
7.46
7.45
7.43
7.24
6.98
6.97
6.89
6.87
6.84
6.20
6.18
6.02
3.98
3.97
2.53
O
NH
NH
O
O
O
O
CH3
0.99
1.00
1.01
1.05
1.05
1.09
1.05
2.06
3.01
1.00
2.06
2.00
3.02
14
13
12
11
10
9
8
7
6
5
4
3
2
1
0
-1
-2

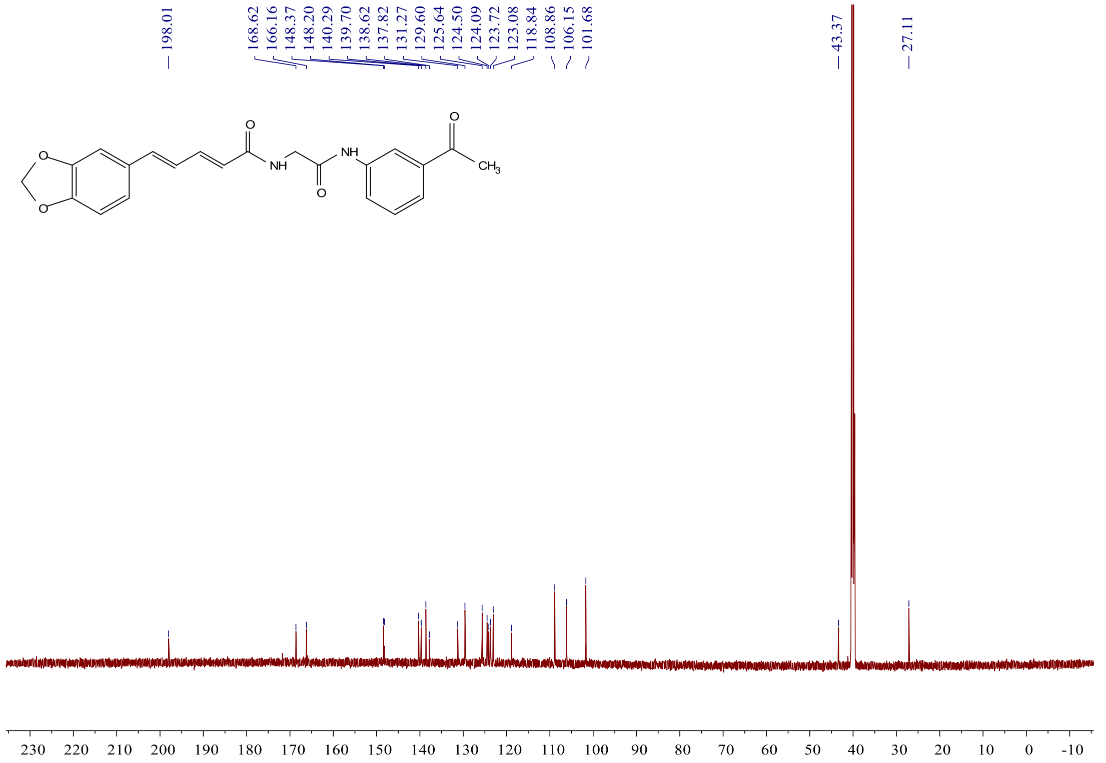

198.01
168.62
166.16
148.37
148.20
140.29
139.70
138.62
137.82
131.27
129.60
125.64
124.50
124.09
123.72
123.08
118.84
108.86
106.15
101.68
43.37
27.11
O
NH
NH
O
O
O
O
CH3
230
220
210
200
190
180
170
160
150
140
130
120
110
100
90
80
70
60
50
40
30
20
10
0
-10

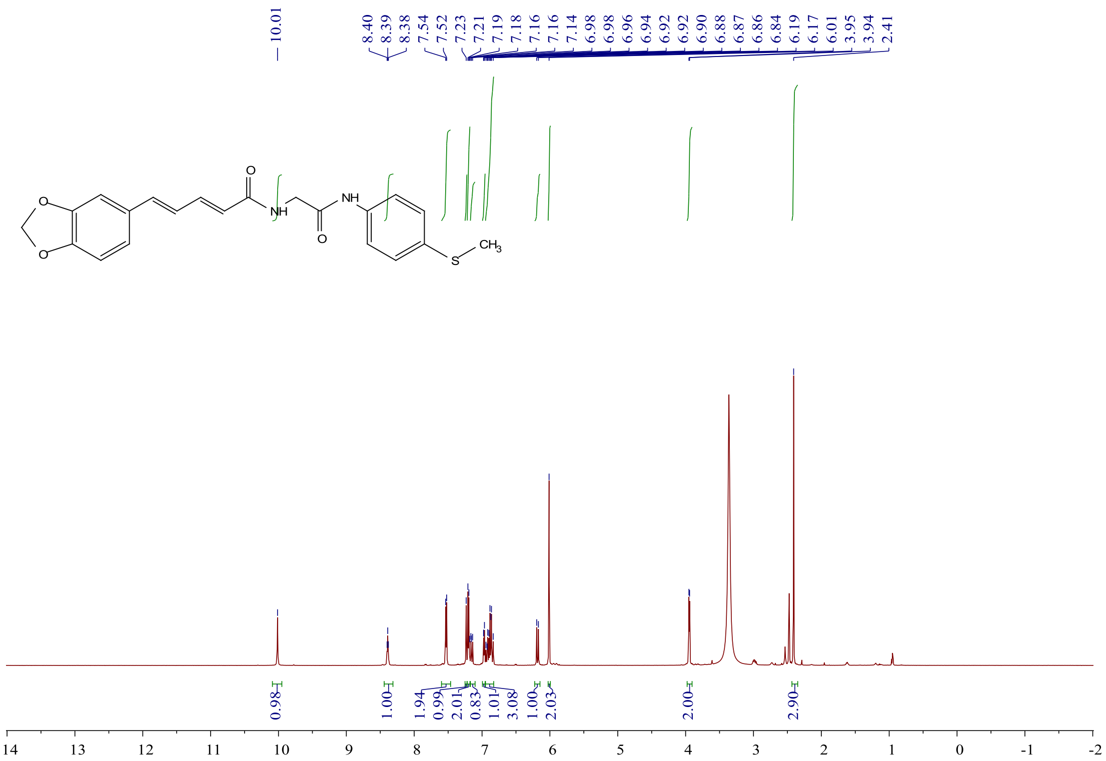
10.01
8.40
8.39
8.38
7.54
7.52
7.23
7.21
7.19
7.18
7.16
7.16
7.14
6.98
6.98
6.96
6.94
6.92
6.92
6.90
6.88
6.87
6.86
6.84
6.19
6.17
6.01
3.95
3.94
2.41
O
NH
NH
O
O
O
S
CH3
0.98
1.00
1.94
0.99
2.01
0.83
1.01
3.08
1.00
2.03
2.00
2.90
14 13 12 11 10 9 8 7 6 5 4 3 2 1 0 -1 -2

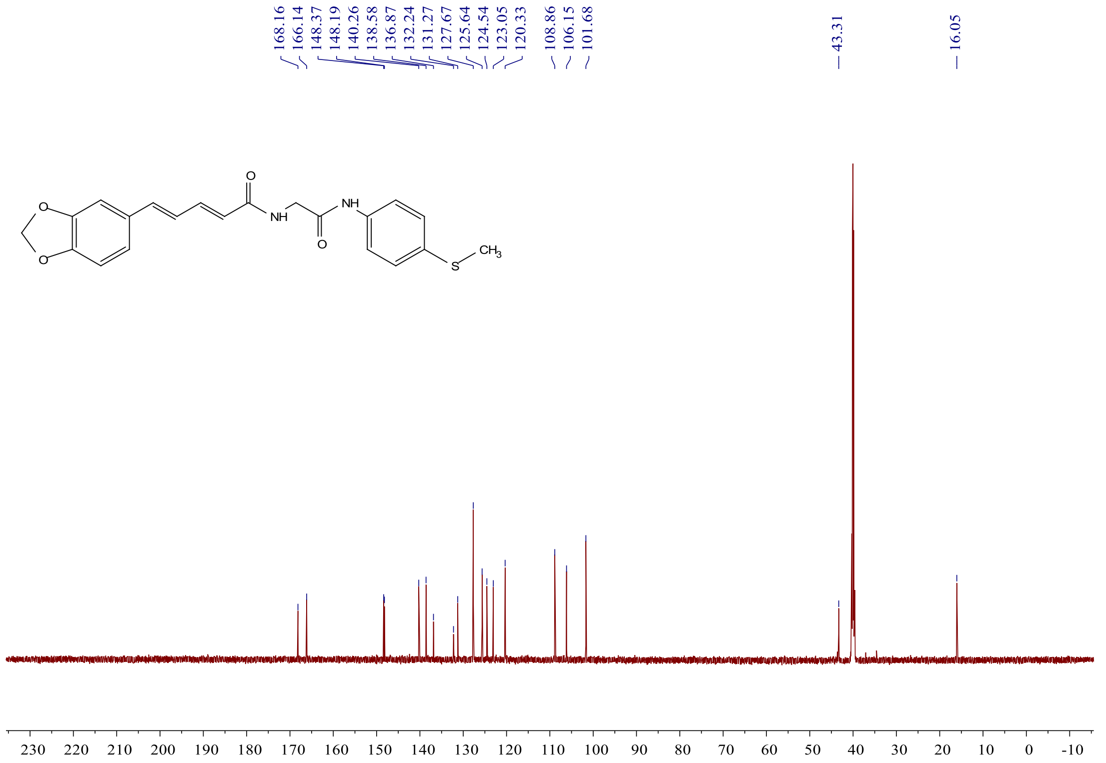
168.16
166.14
148.37
148.19
140.26
138.58
136.87
132.24
131.27
127.67
125.64
124.54
123.05
120.33
108.86
106.15
101.68
43.31
16.05
O
NH
NH
O
O
O
S
CH3
230 220 210 200 190 180 170 160 150 140 130 120 110 100 90 80 70 60 50 40 30 20 10 0 -10

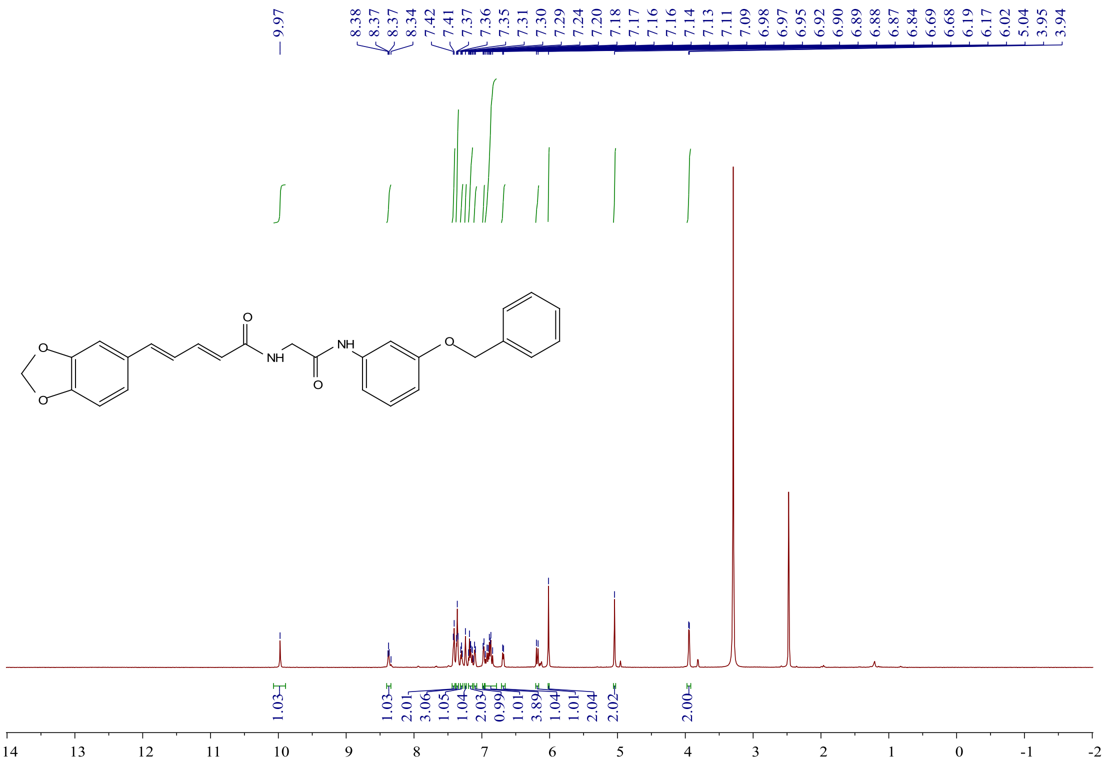
9.97
8.38
8.37
8.37
8.34
7.42
7.41
7.37
7.36
7.35
7.31
7.30
7.29
7.24
7.20
7.18
7.17
7.16
7.16
7.14
7.13
7.11
7.09
6.98
6.97
6.95
6.92
6.90
6.89
6.88
6.87
6.84
6.69
6.68
6.19
6.17
6.02
5.04
3.95
3.94
O
O
O
NH
O
NH
O
1.03
1.03
2.01
3.06
1.05
1.04
2.03
0.99
1.01
3.89
1.04
1.01
2.04
2.02
2.00
14 13 12 11 10 9 8 7 6 5 4 3 2 1 0 -1 -2

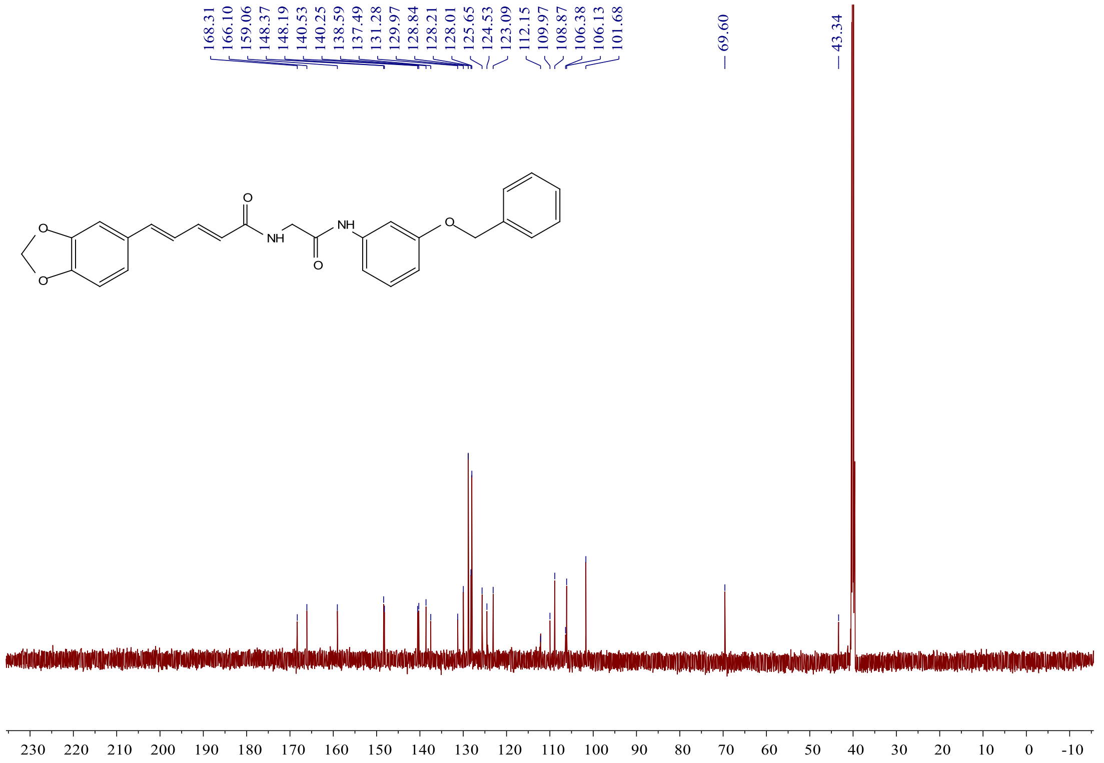
168.31
166.10
159.06
148.37
148.19
140.53
140.25
138.59
137.49
131.28
129.97
128.84
128.21
128.01
125.65
124.53
123.09
112.15
109.97
108.87
106.38
106.13
101.68
69.60
43.34
O
O
O
NH
O
NH
O
230 220 210 200 190 180 170 160 150 140 130 120 110 100 90 80 70 60 50 40 30 20 10 0 -10

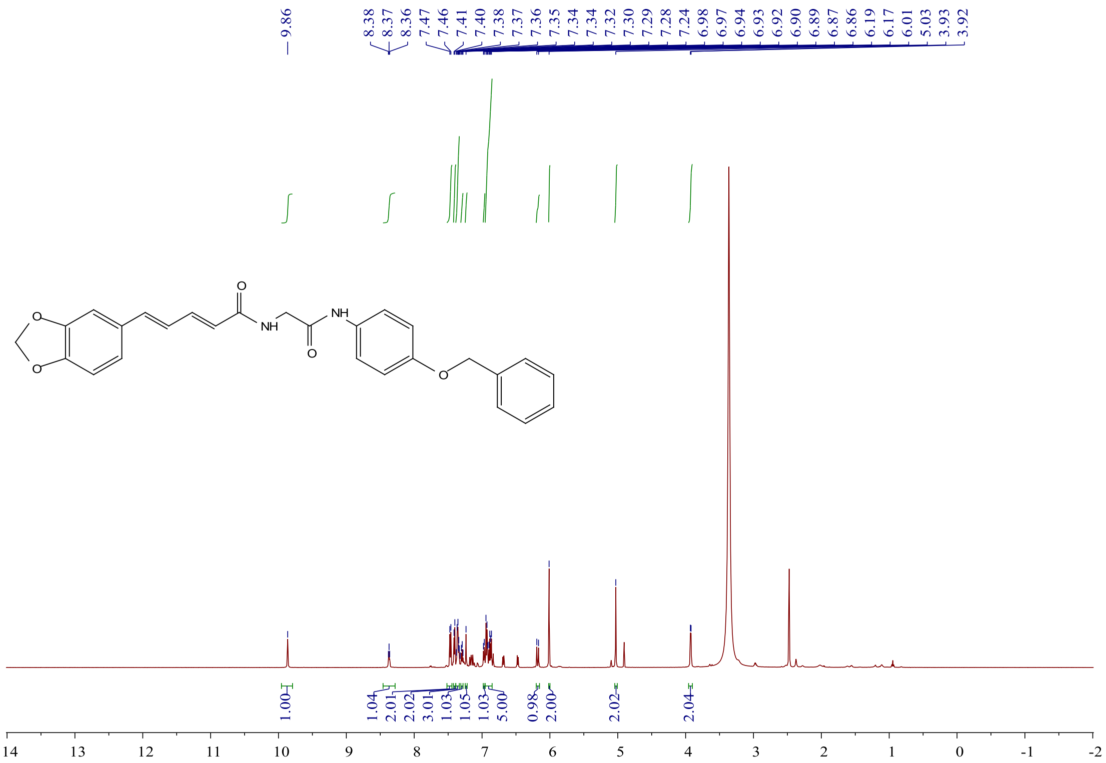
9.86
8.38 8.37 8.36 7.47 7.46 7.41 7.40 7.38 7.37 7.36 7.35 7.34 7.34 7.32 7.30 7.29 7.28 7.24 6.98 6.97 6.94 6.93 6.92 6.90 6.89 6.87 6.86 6.19 6.17 6.01 5.03 3.93 3.92
O
NH
NH
O
O
O
O
1.00
1.04 2.01 2.02 3.01 1.03 1.05 1.03 5.00
0.98 2.00
2.02
2.04
14 13 12 11 10 9 8 7 6 5 4 3 2 1 0 -1 -2

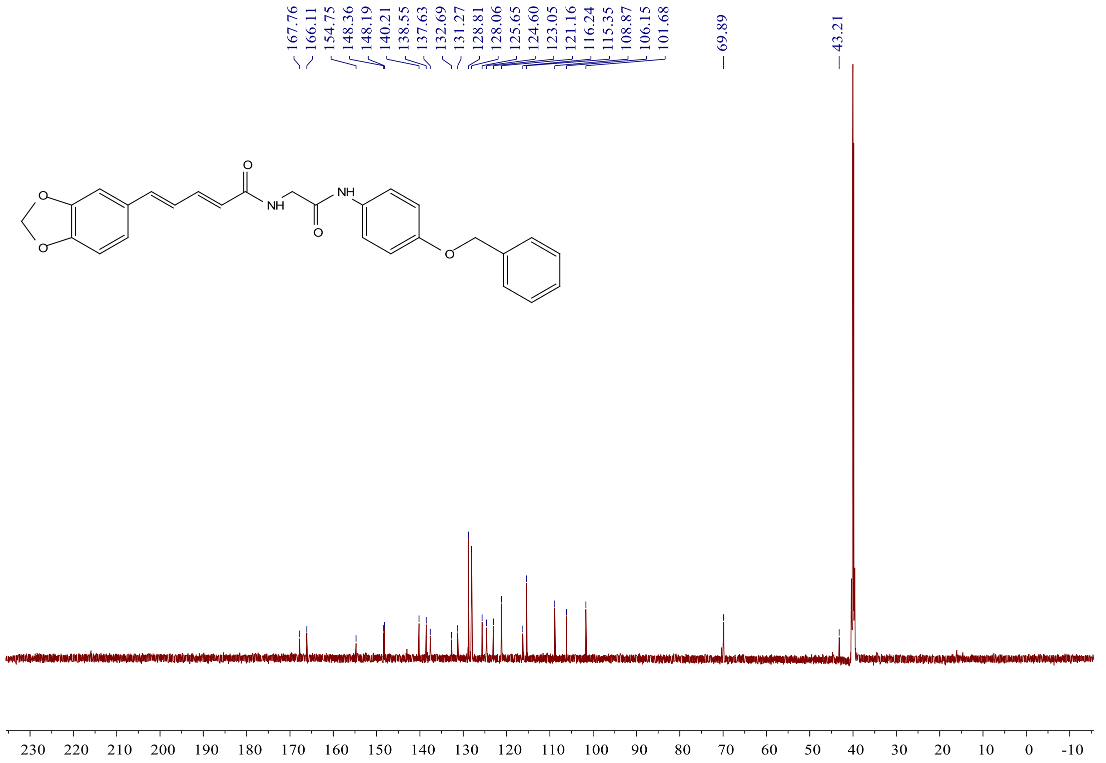
167.76 166.11 154.75 148.36 148.19 140.21 138.55 137.63 132.69 131.27 128.81 128.06 125.65 124.60 123.05 121.16 116.24 115.35 108.87 106.15 101.68
69.89
43.21
O
NH
NH
O
O
O
O
230 220 210 200 190 180 170 160 150 140 130 120 110 100 90 80 70 60 50 40 30 20 10 0 -10

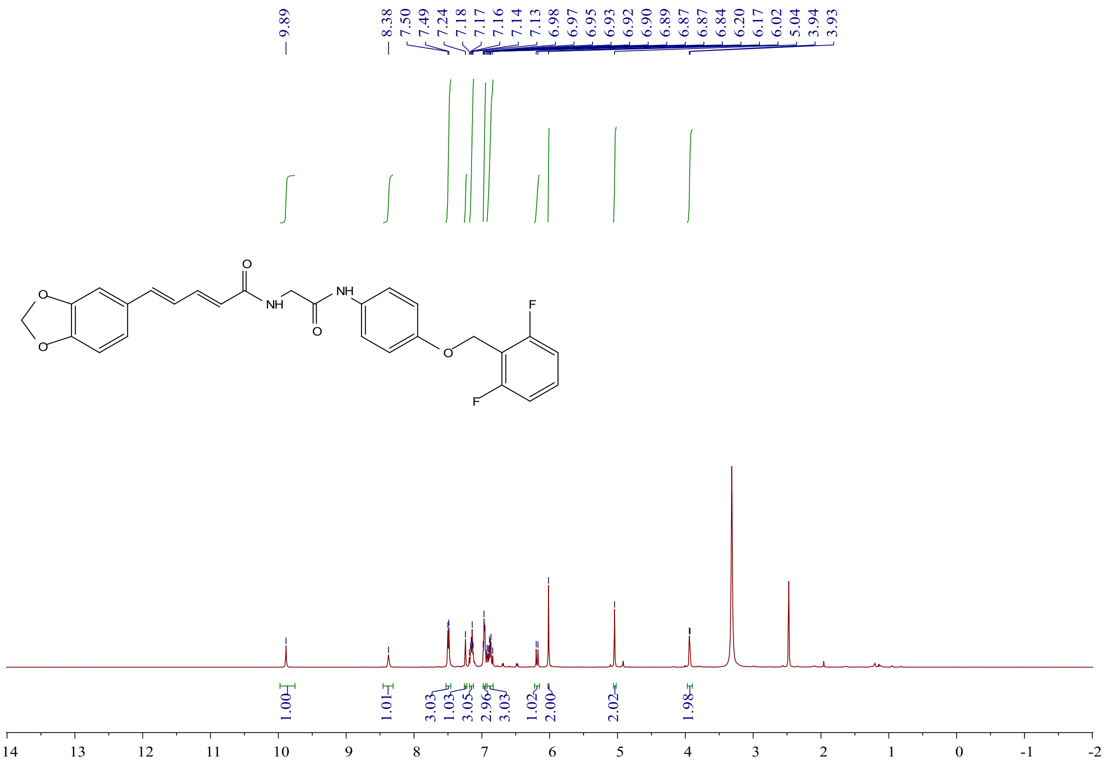
9.89
8.38
7.50
7.49
7.24
7.18
7.17
7.16
7.14
7.13
6.98
6.97
6.95
6.93
6.92
6.90
6.89
6.87
6.84
6.20
6.17
6.02
5.04
3.94
3.93
O
O
O
NH
NH
O
O
F
F
1.00
1.01
3.03
1.03
3.05
2.96
3.03
1.02
2.00
2.02
1.98
14 13 12 11 10 9 8 7 6 5 4 3 2 1 0 -1 -2

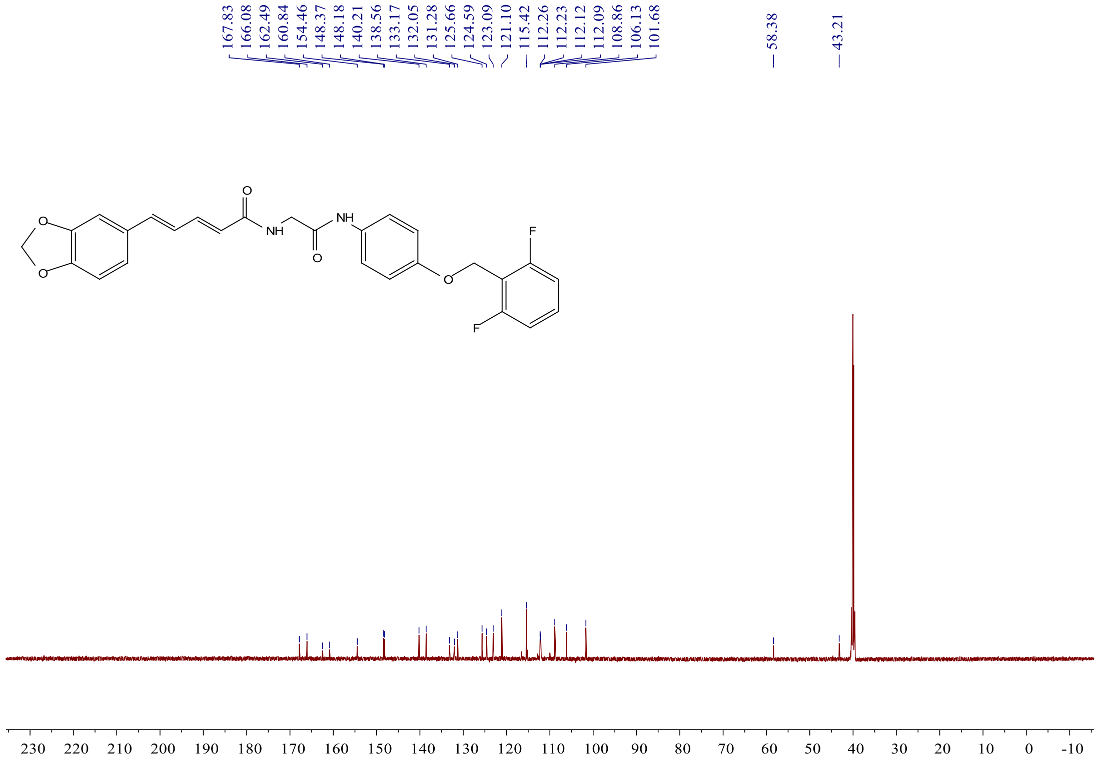
167.83
166.08
162.49
160.84
154.46
148.37
148.18
140.21
138.56
133.17
132.05
131.28
125.66
124.59
123.09
121.10
115.42
112.26
112.23
112.12
112.09
108.86
106.13
101.68
58.38
43.21
O
O
O
NH
NH
O
O
F
F
230 220 210 200 190 180 170 160 150 140 130 120 110 100 90 80 70 60 50 40 30 20 10 0 -10

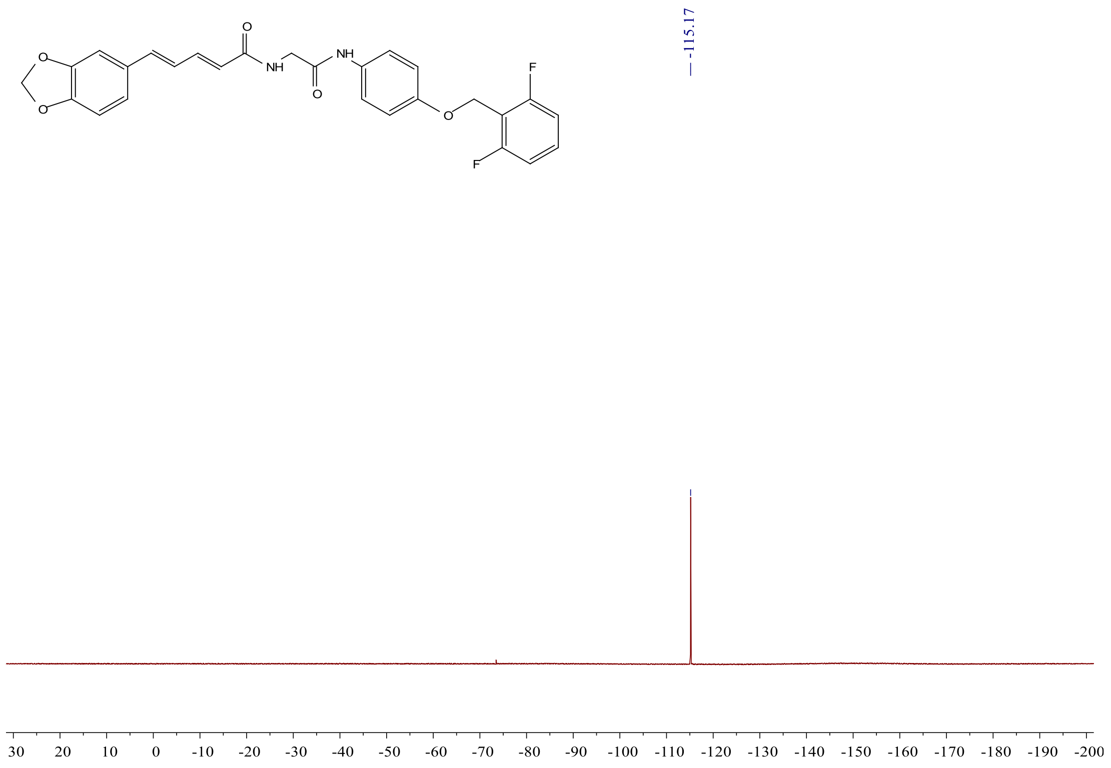

O
NH
F
-115.17
30 20 10 0 -10 -20 -30 -40 -50 -60 -70 -80 -90 -100 -110 -120 -130 -140 -150 -160 -170 -180 -190 -200

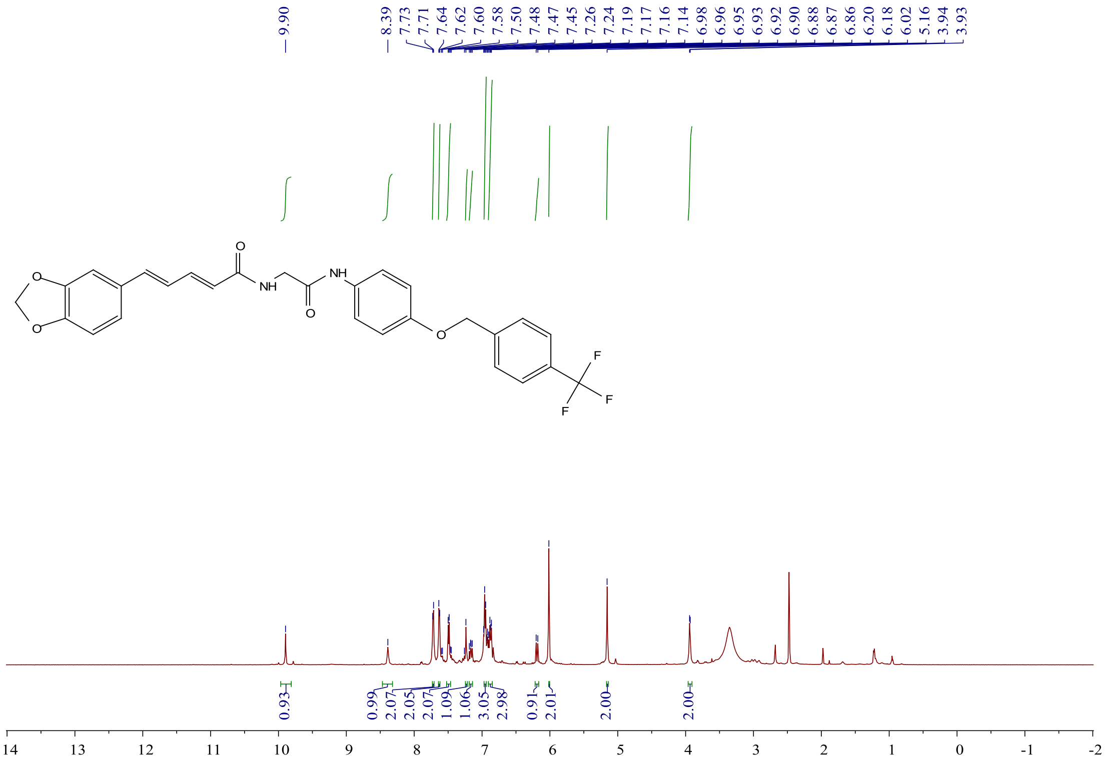

9.90
8.39 7.73 7.71 7.64 7.62 7.60 7.58 7.50 7.48 7.47 7.45 7.26 7.24 7.19 7.17 7.16 7.14 6.98 6.96 6.95 6.93 6.92 6.90 6.88 6.87 6.86 6.20 6.18 6.02 5.16 3.94 3.93
O
NH
F
0.93
0.99 2.07 2.05 2.07 1.09 1.06 3.05 2.98 0.91 2.01 2.00 2.00
14 13 12 11 10 9 8 7 6 5 4 3 2 1 0 -1 -2

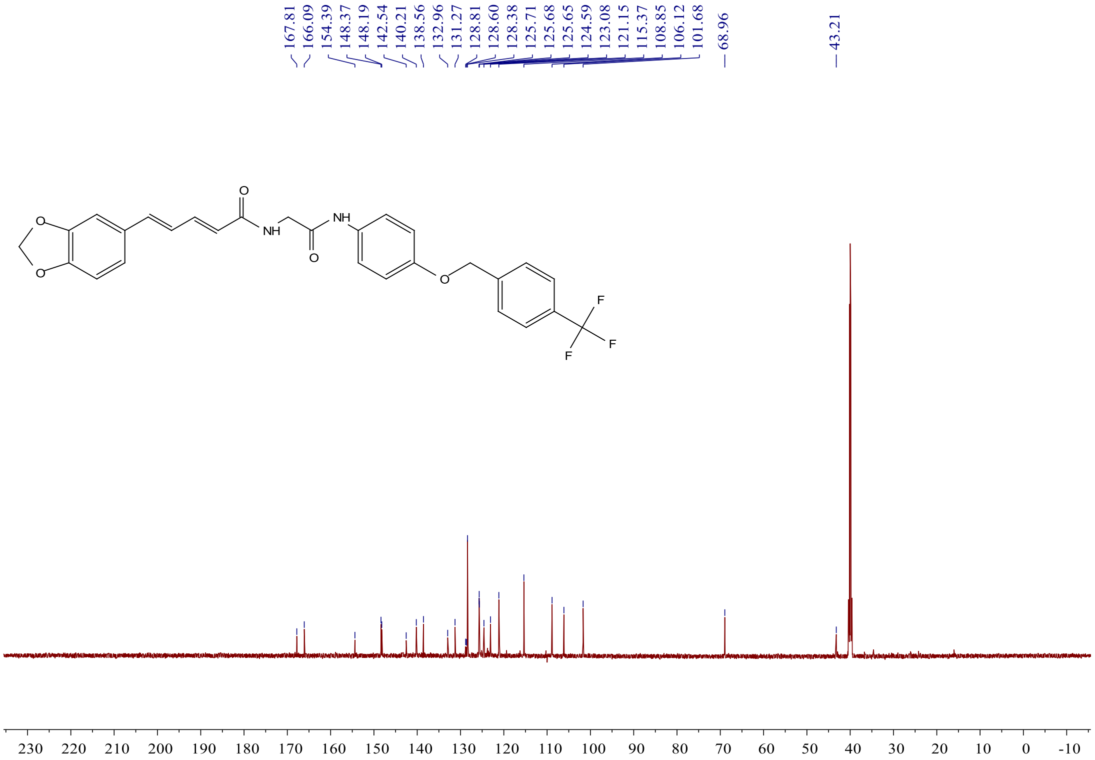
167.81
166.09
154.39
148.37
148.19
142.54
140.21
138.56
132.96
131.27
128.81
128.60
128.38
125.71
125.68
125.65
124.59
123.08
121.15
115.37
108.85
106.12
101.68
68.96
43.21
230 220 210 200 190 180 170 160 150 140 130 120 110 100 90 80 70 60 50 40 30 20 10 0 -10

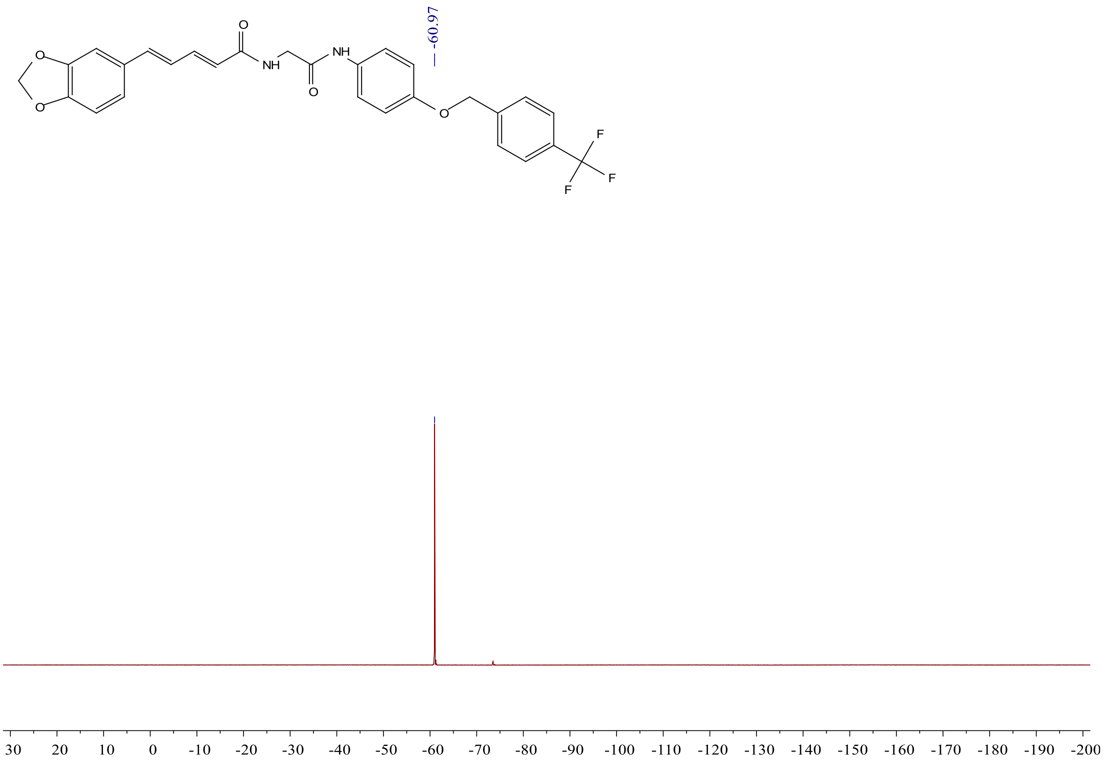
-60.97
30 20 10 0 -10 -20 -30 -40 -50 -60 -70 -80 -90 -100 -110 -120 -130 -140 -150 -160 -170 -180 -190 -200

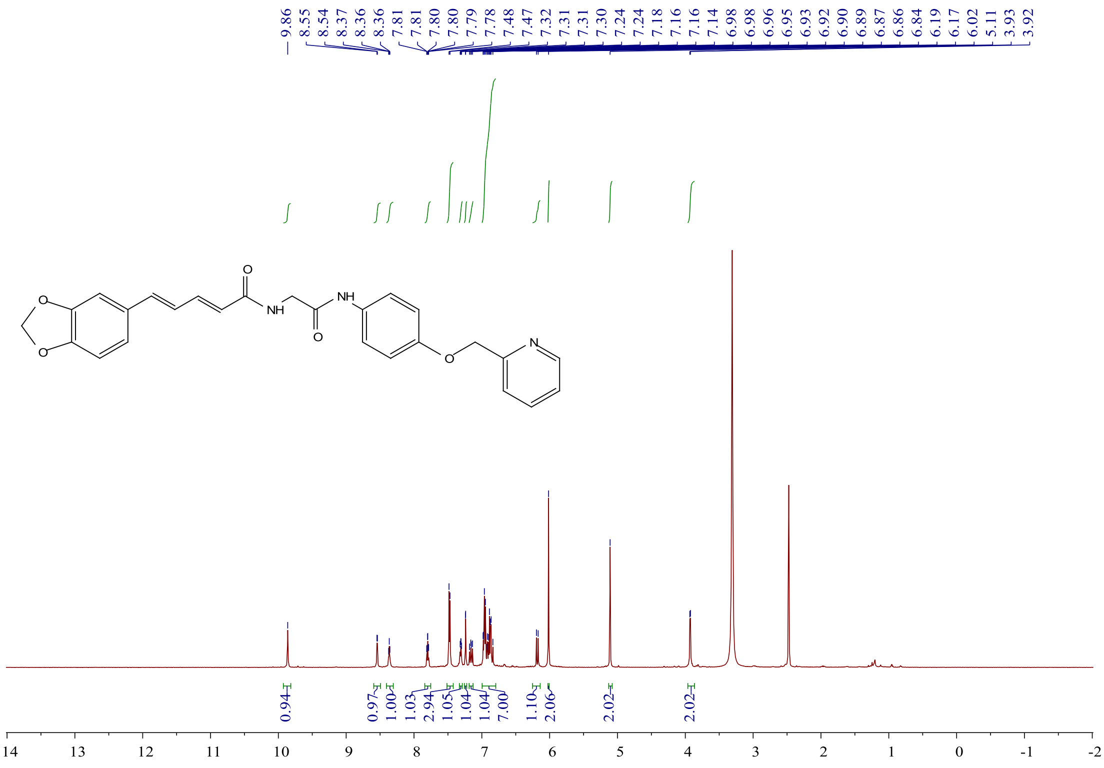

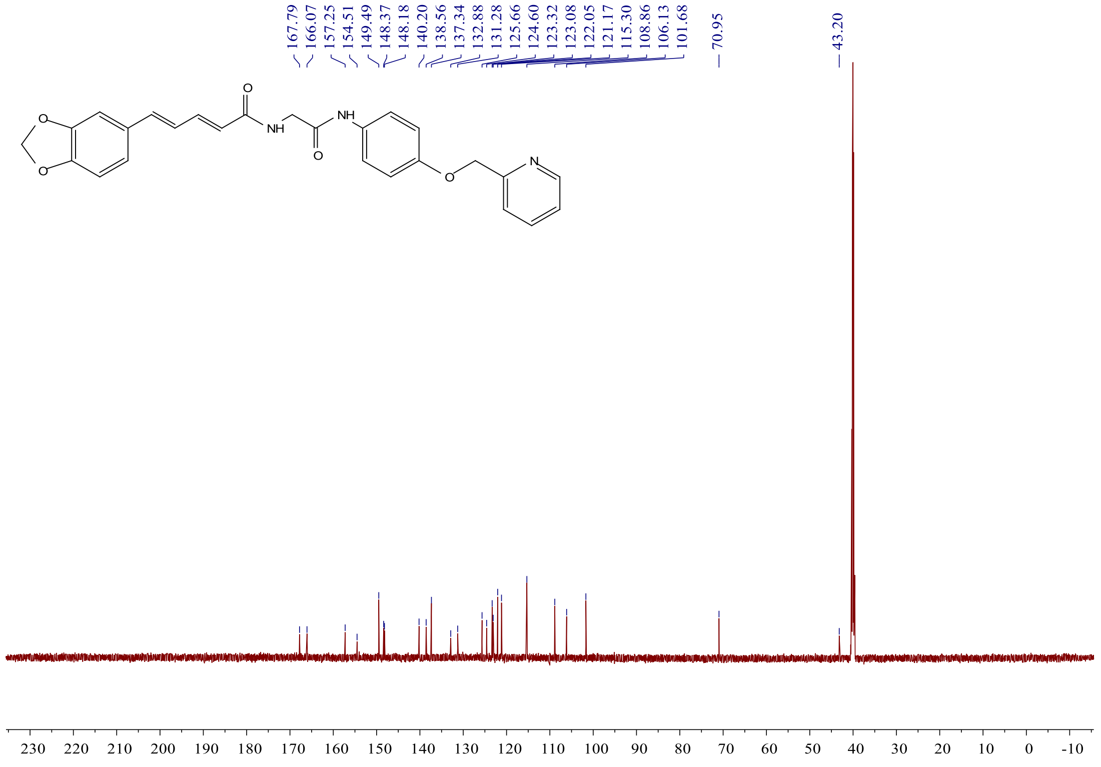

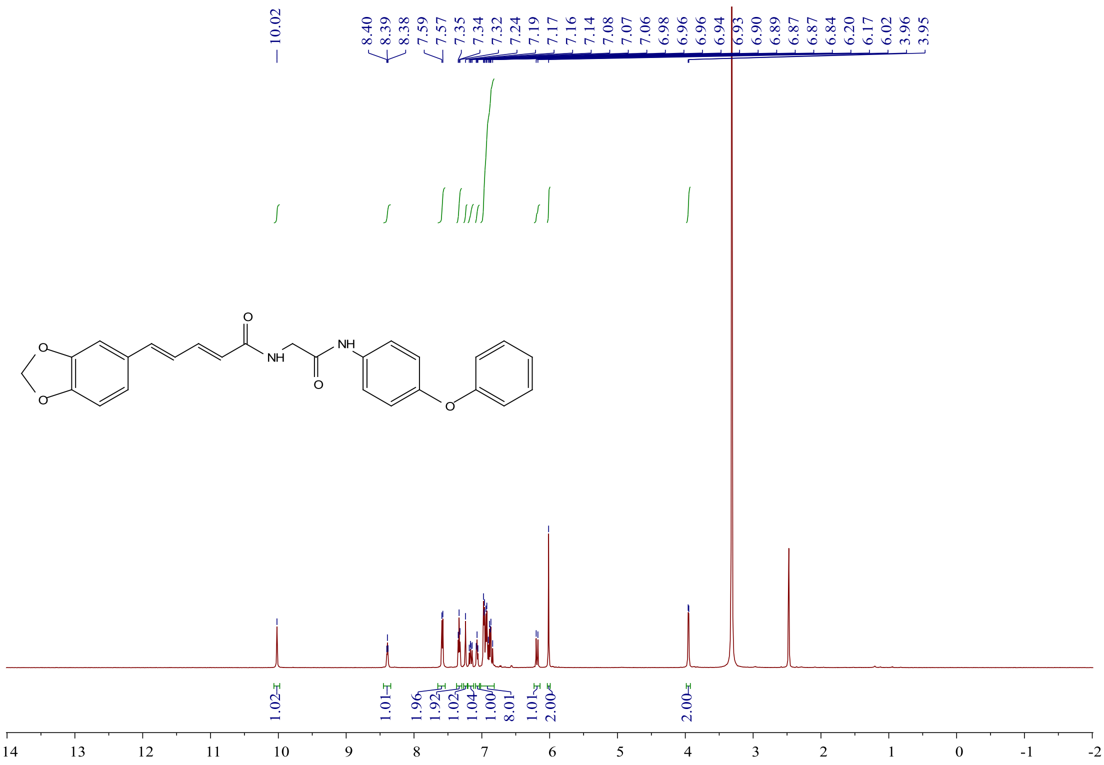
10.02
8.40
8.39
8.38
7.59
7.57
7.35
7.34
7.32
7.24
7.19
7.17
7.16
7.14
7.08
7.07
7.06
6.98
6.96
6.96
6.94
6.93
6.90
6.89
6.87
6.87
6.84
6.20
6.17
6.02
3.96
3.95
O
NH
NH
O
O
O
O
1.02
1.01
1.96
1.92
1.02
1.04
1.00
8.01
1.01
2.00
2.00
14 13 12 11 10 9 8 7 6 5 4 3 2 1 0 -1 -2

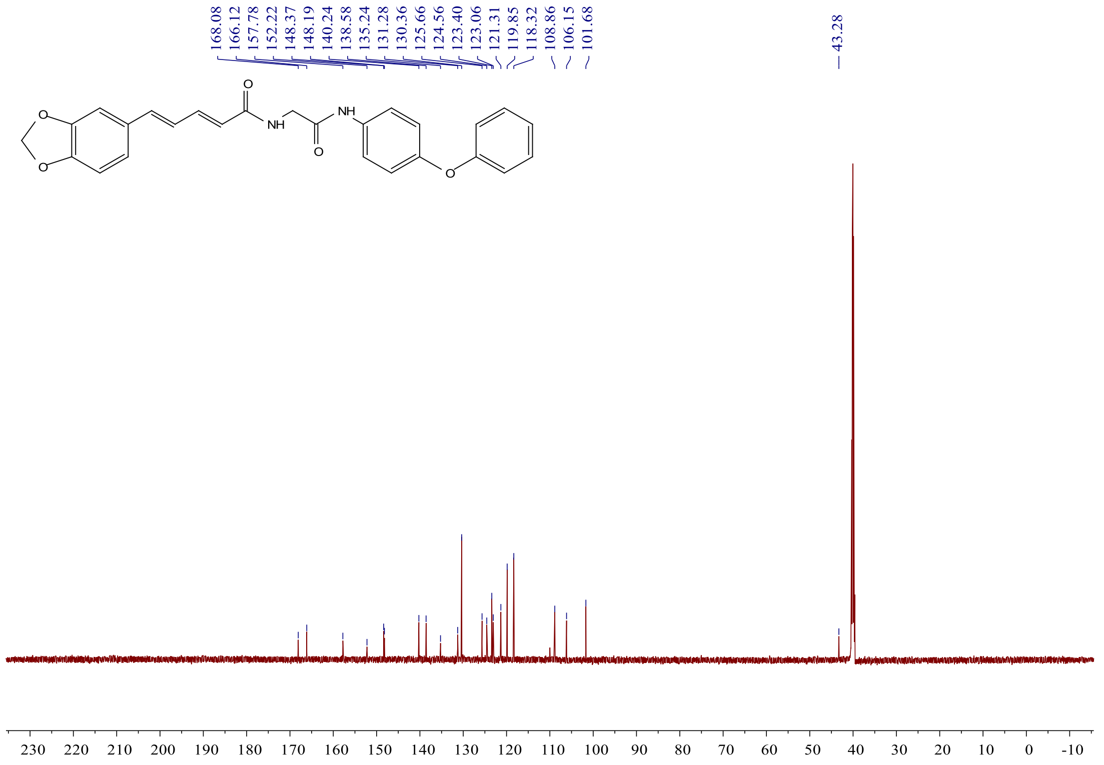
168.08
166.12
157.78
152.22
148.37
148.19
140.24
138.58
135.24
131.28
130.36
125.66
124.56
123.40
123.06
121.31
119.85
118.32
108.86
106.15
101.68
43.28
O
NH
NH
O
O
O
O
230 220 210 200 190 180 170 160 150 140 130 120 110 100 90 80 70 60 50 40 30 20 10 0 -10

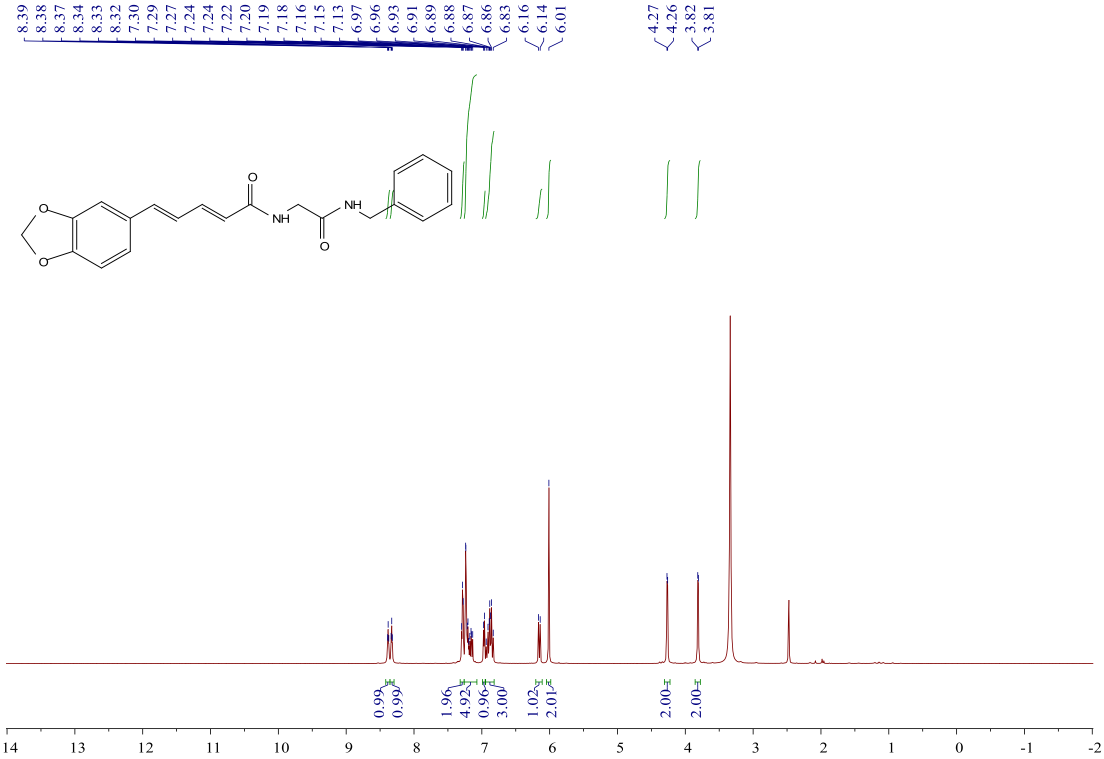
8.39 8.38 8.37 8.34 8.33 8.32 7.30 7.29 7.27 7.24 7.22 7.20 7.19 7.18 7.16 7.15 7.13 6.97 6.96 6.93 6.91 6.89 6.88 6.87 6.86 6.83 6.16 6.14 6.01
4.27 4.26 3.82 3.81
O
NH
NH
O
O
O
0.99 0.99 1.96 4.92 0.96 3.00 1.02 2.01 2.00 2.00
14 13 12 11 10 9 8 7 6 5 4 3 2 1 0 -1 -2

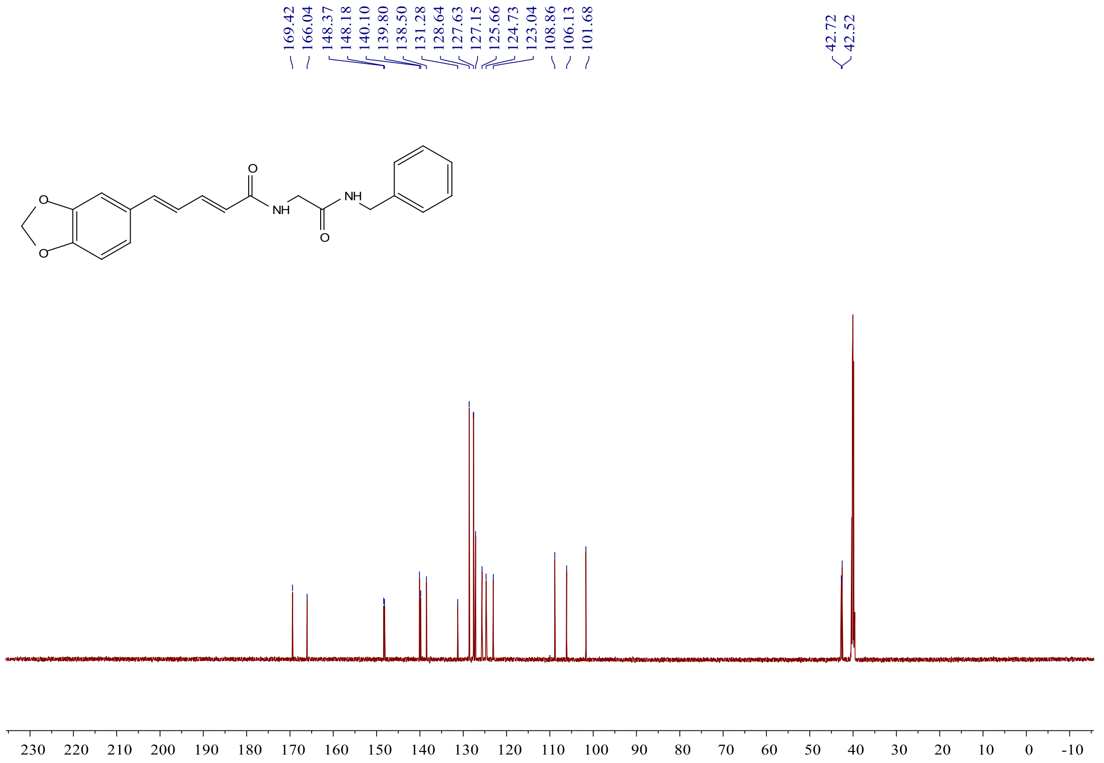
169.42 166.04 148.37 148.18 140.10 139.80 138.50 131.28 128.64 127.63 127.15 125.66 124.73 123.04 108.86 106.13 101.68
42.72 42.52
O
NH
NH
O
O
O
230 220 210 200 190 180 170 160 150 140 130 120 110 100 90 80 70 60 50 40 30 20 10 0 -10

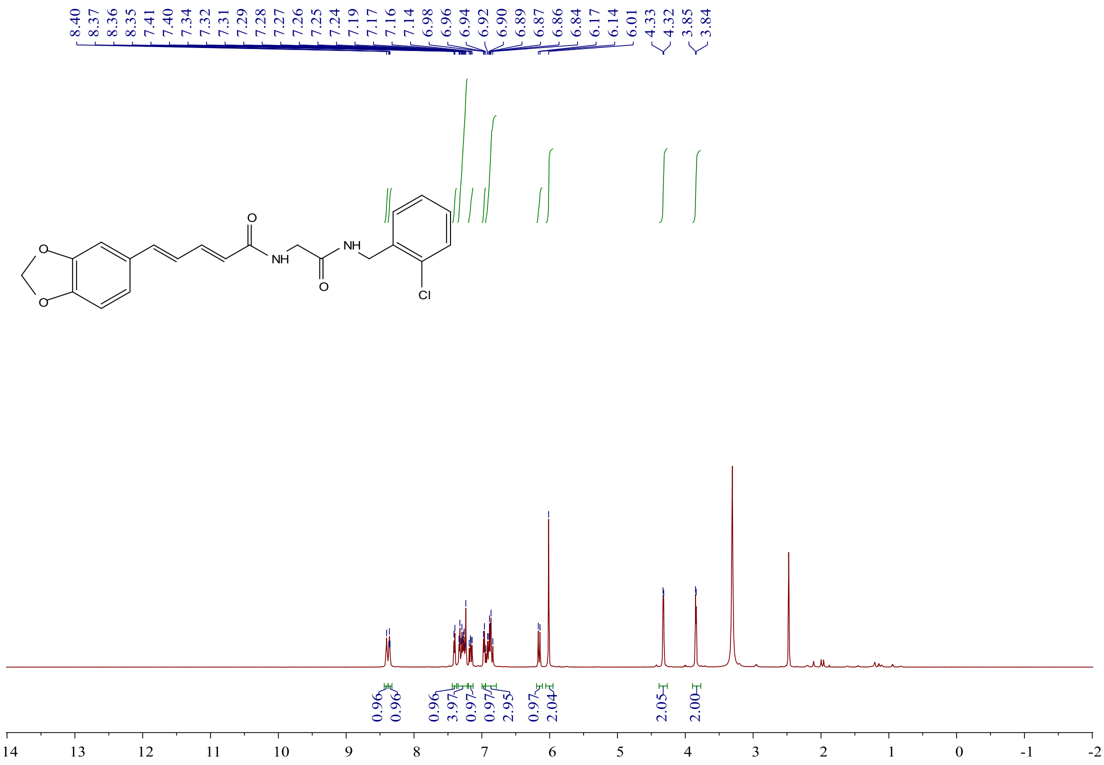
8.40 8.37 8.36 8.35 7.41 7.40 7.34 7.32 7.31 7.29 7.28 7.27 7.26 7.25 7.24 7.19 7.17 7.16 7.14 6.98 6.96 6.94 6.92 6.90 6.89 6.87 6.86 6.84 6.17 6.14 6.01 4.33 4.32 3.85 3.84
O
NH
NH
O
Cl
0.96 0.96 0.96 3.97 0.97 0.97 2.95 0.97 2.04 2.05 2.00
14 13 12 11 10 9 8 7 6 5 4 3 2 1 0 -1 -2

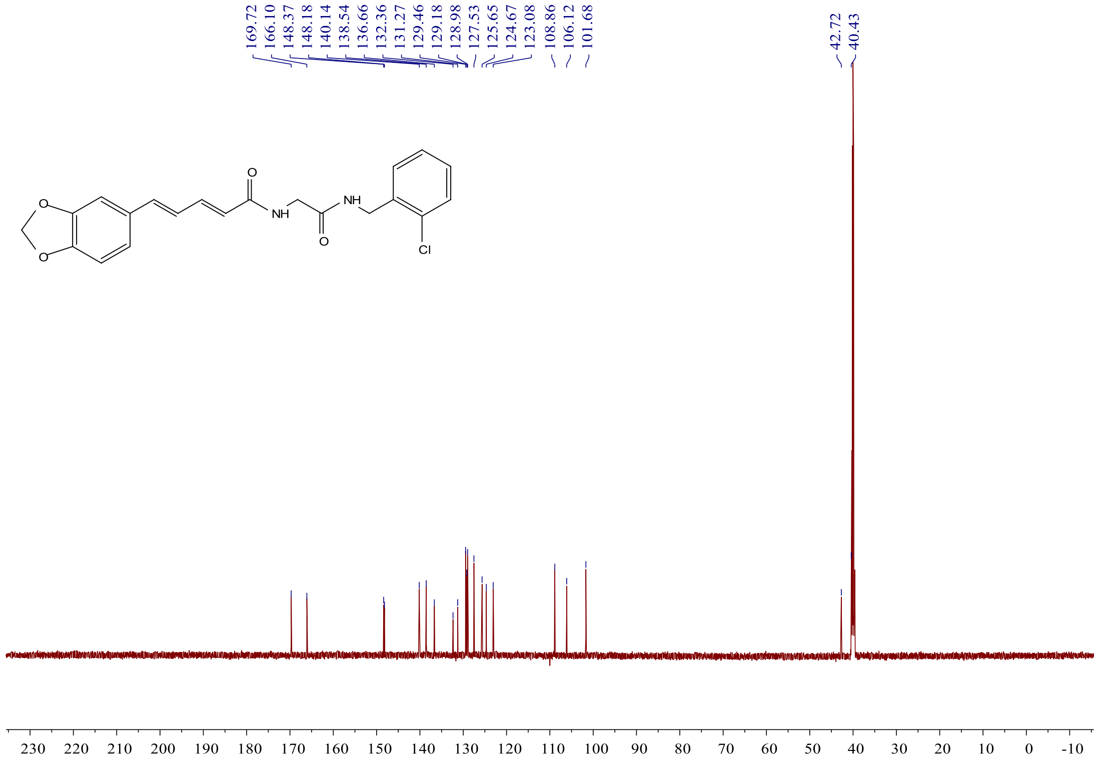
169.72 166.10 148.37 148.18 140.14 138.54 136.66 132.36 131.27 129.46 129.18 128.98 127.53 125.65 124.67 123.08 108.86 106.12 101.68
42.72 40.43
O
NH
NH
O
Cl
230 220 210 200 190 180 170 160 150 140 130 120 110 100 90 80 70 60 50 40 30 20 10 0 -10

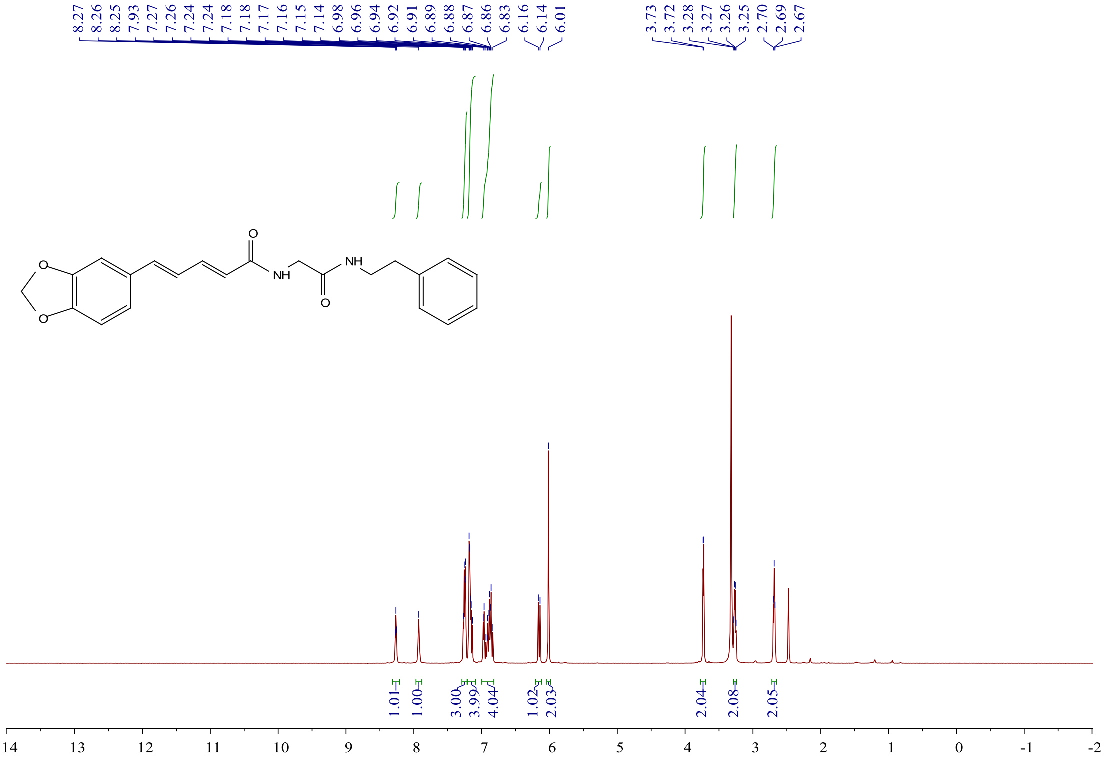
8.27
8.26
8.25
7.93
7.27
7.26
7.24
7.18
7.18
7.17
7.16
7.15
7.14
6.98
6.96
6.94
6.92
6.91
6.89
6.88
6.87
6.86
6.83
6.16
6.14
6.01
3.73
3.72
3.28
3.27
3.26
3.25
2.70
2.69
2.67
O
NH
NH
O
O
O
1.01
1.00
3.00
3.99
4.04
1.02
2.03
2.04
2.08
2.05
14 13 12 11 10 9 8 7 6 5 4 3 2 1 0 -1 -2

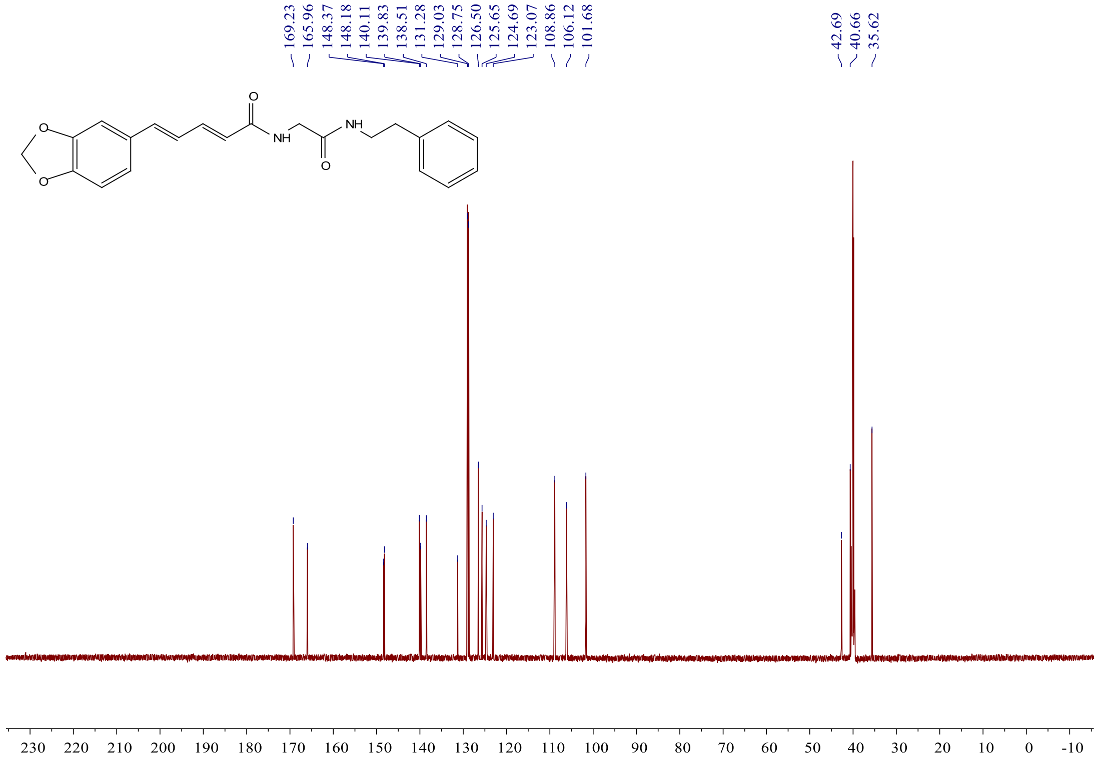
169.23
165.96
148.37
148.18
140.11
139.83
138.51
131.28
129.03
128.75
126.50
125.65
124.69
123.07
108.86
106.12
101.68
42.69
40.66
35.62
O
NH
NH
O
O
O
230 220 210 200 190 180 170 160 150 140 130 120 110 100 90 80 70 60 50 40 30 20 10 0 -10

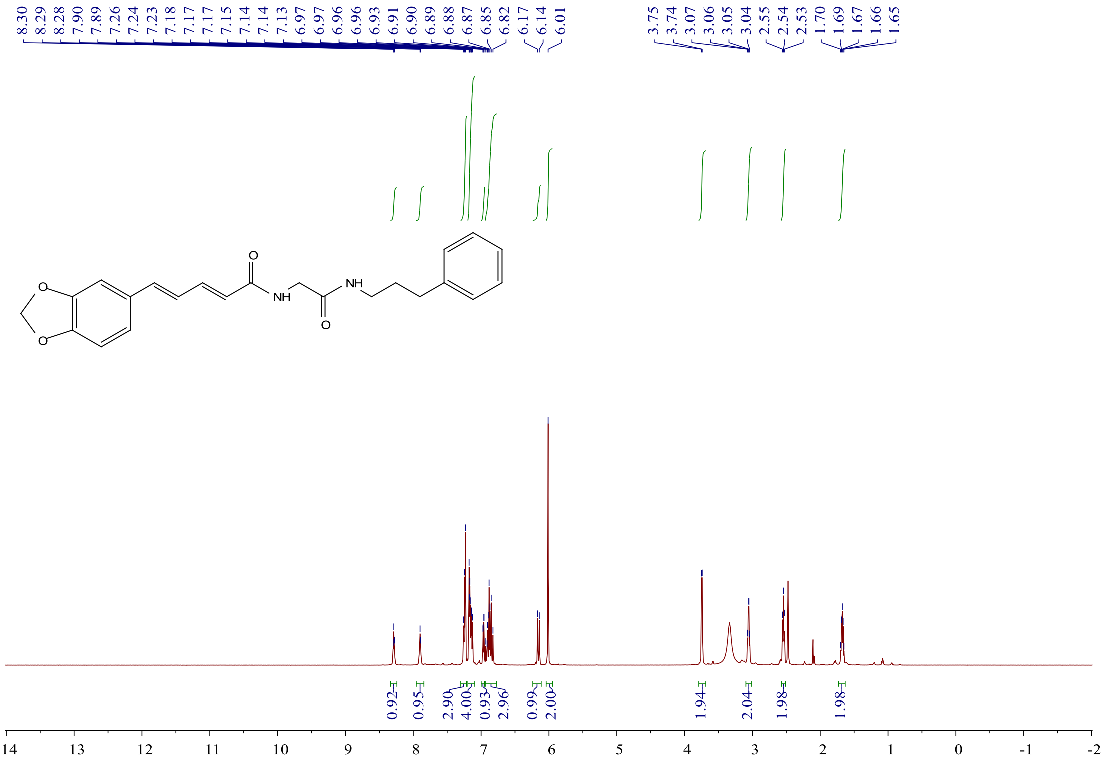
8.30 8.29 8.28 7.90 7.89 7.26 7.24 7.23 7.18 7.17 7.17 7.15 7.14 7.14 7.13 6.97 6.97 6.96 6.96 6.93 6.91 6.90 6.89 6.88 6.87 6.85 6.82 6.17 6.14 6.01
3.75 3.74 3.07 3.06 3.05 3.04 2.55 2.54 2.53 1.70 1.69 1.67 1.66 1.65
O
O
O
NH
NH
O
0.92 0.95 2.90 4.00 0.93 2.96 0.99 2.00 1.94 2.04 1.98 1.98
14 13 12 11 10 9 8 7 6 5 4 3 2 1 0 -1 -2

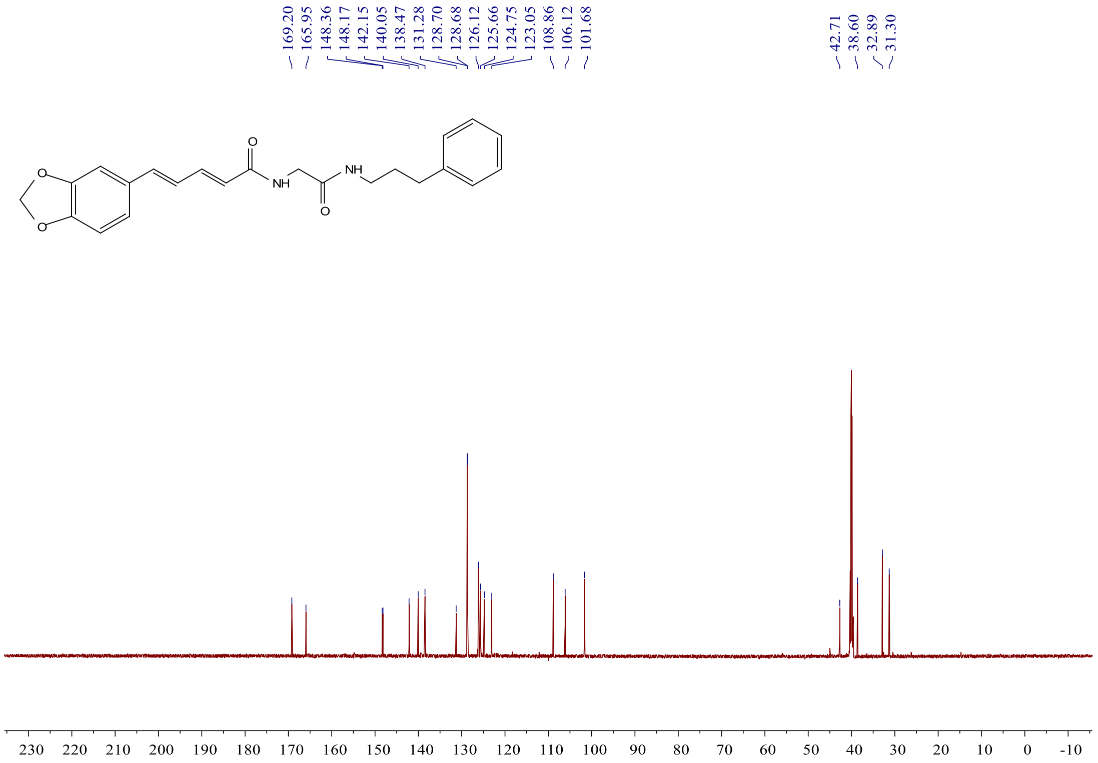
169.20 165.95 148.36 148.17 142.15 140.05 138.47 131.28 128.70 128.68 126.12 125.66 124.75 123.05 108.86 106.12 101.68
42.71 38.60 32.89 31.30
O
O
O
NH
NH
O
230 220 210 200 190 180 170 160 150 140 130 120 110 100 90 80 70 60 50 40 30 20 10 0 -10

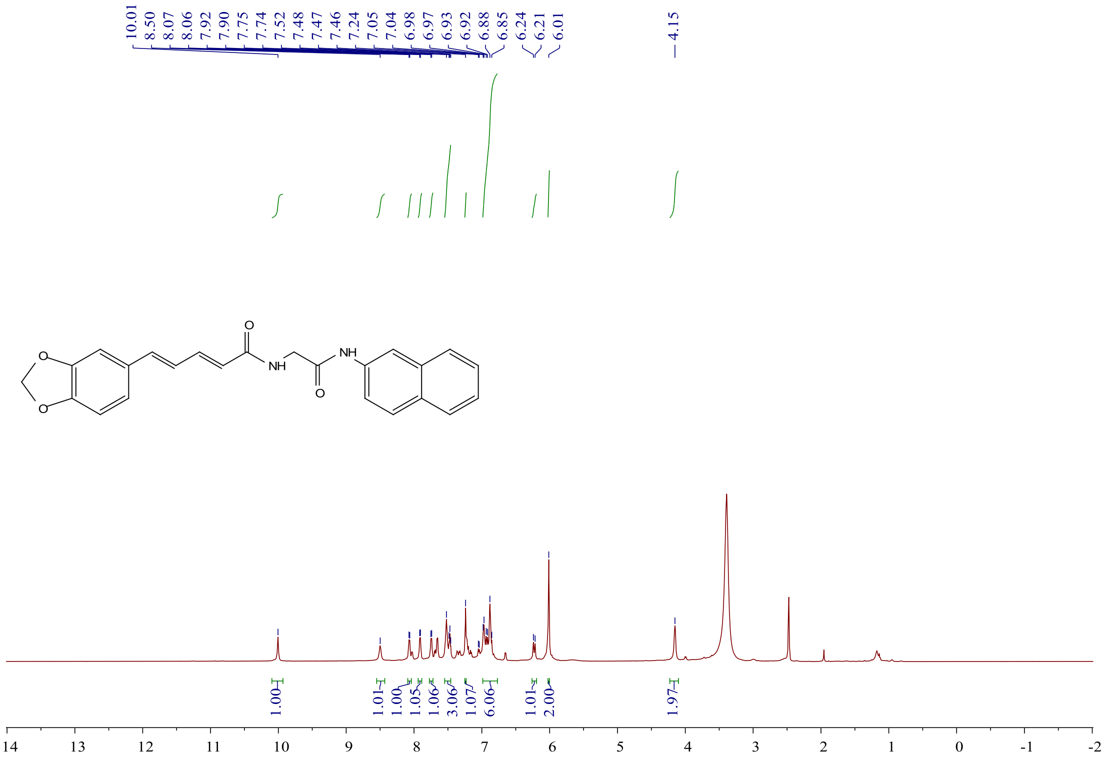
10.01
8.50
8.07
8.06
7.92
7.90
7.75
7.74
7.52
7.48
7.47
7.46
7.24
7.05
7.04
6.98
6.97
6.93
6.92
6.88
6.85
6.24
6.21
6.01
4.15
O
NH
1.00
1.01
1.00
1.05
1.06
3.06
1.07
6.06
1.01
2.00
1.97
14
13
12
11
10
9
8
7
6
5
4
3
2
1
0
-1
-2

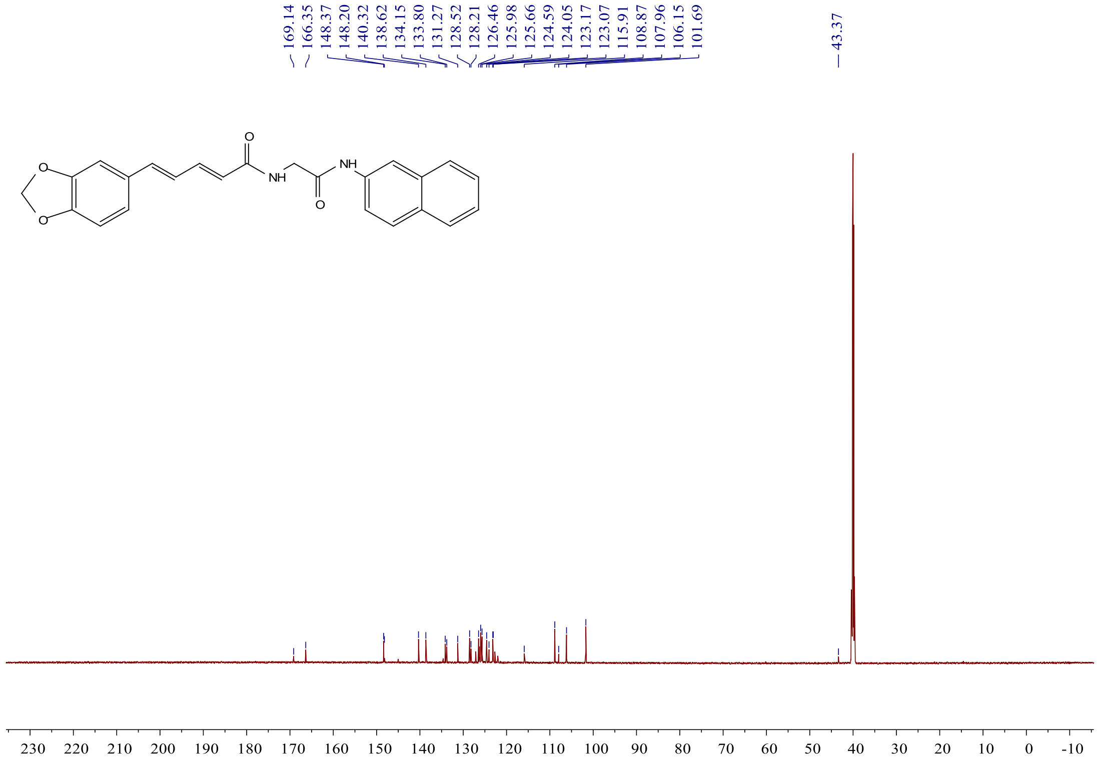
169.14
166.35
148.37
148.20
140.32
138.62
134.15
133.80
131.27
128.52
128.21
126.46
125.98
125.66
124.59
124.05
123.17
123.07
115.91
108.87
107.96
106.15
101.69
43.37
O
NH
230
220
210
200
190
180
170
160
150
140
130
120
110
100
90
80
70
60
50
40
30
20
10
0
-10

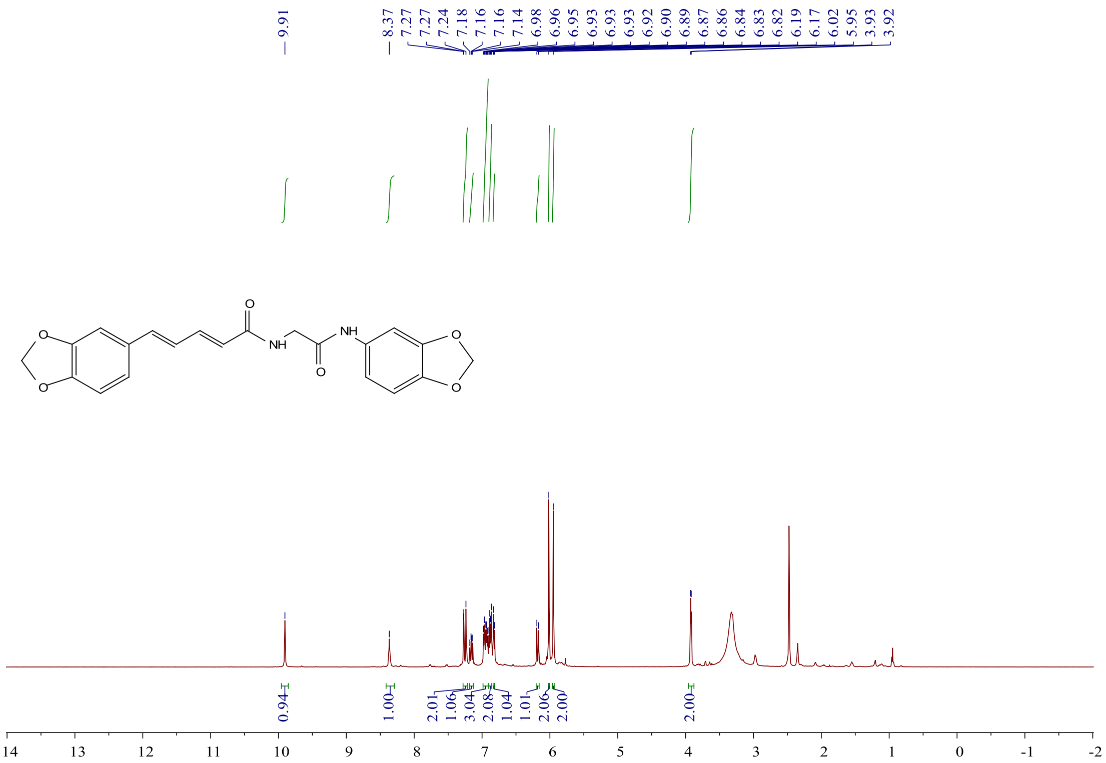

9.91
8.37
7.27
7.27
7.24
7.18
7.16
7.16
7.14
6.98
6.96
6.95
6.93
6.93
6.93
6.92
6.90
6.89
6.87
6.86
6.84
6.83
6.82
6.19
6.17
6.02
5.95
3.93
3.92
O
NH
NH
O
O
O
O
O
0.94
1.00
2.01
1.06
3.04
2.08
1.04
1.01
2.06
2.00
2.00
14 13 12 11 10 9 8 7 6 5 4 3 2 1 0 -1 -2

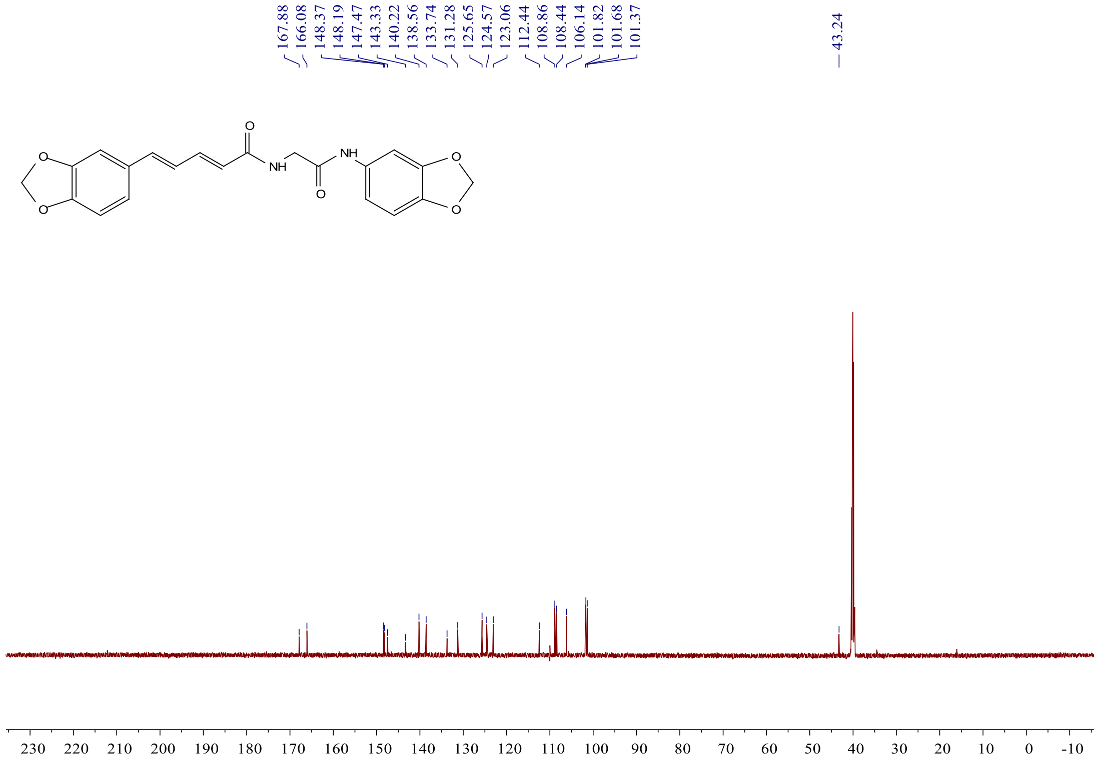

167.88
166.08
148.37
148.19
147.47
143.33
140.22
138.56
133.74
131.28
125.65
124.57
123.06
112.44
108.86
108.44
106.14
101.82
101.68
101.37
43.24
O
NH
NH
O
O
O
O
O
230 220 210 200 190 180 170 160 150 140 130 120 110 100 90 80 70 60 50 40 30 20 10 0 -10

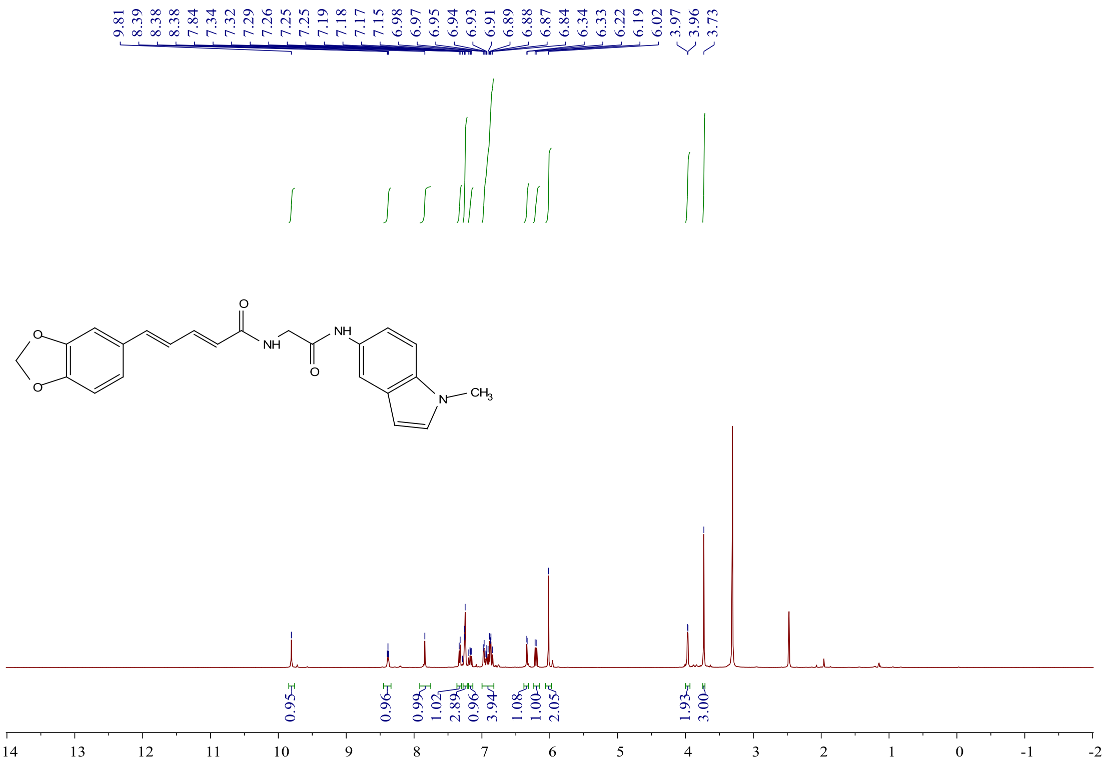
9.81 8.39 8.38 8.38 7.84 7.34 7.32 7.29 7.26 7.25 7.25 7.19 7.18 7.17 7.15 6.98 6.97 6.95 6.94 6.93 6.91 6.89 6.88 6.87 6.84 6.34 6.33 6.22 6.19 6.02 3.97 3.96 3.73
NH
NH
O
O
N
CH_3
0.95 0.96 0.99 1.02 2.89 0.96 3.94 1.08 1.00 2.05 1.93 3.00
14 13 12 11 10 9 8 7 6 5 4 3 2 1 0 -1 -2

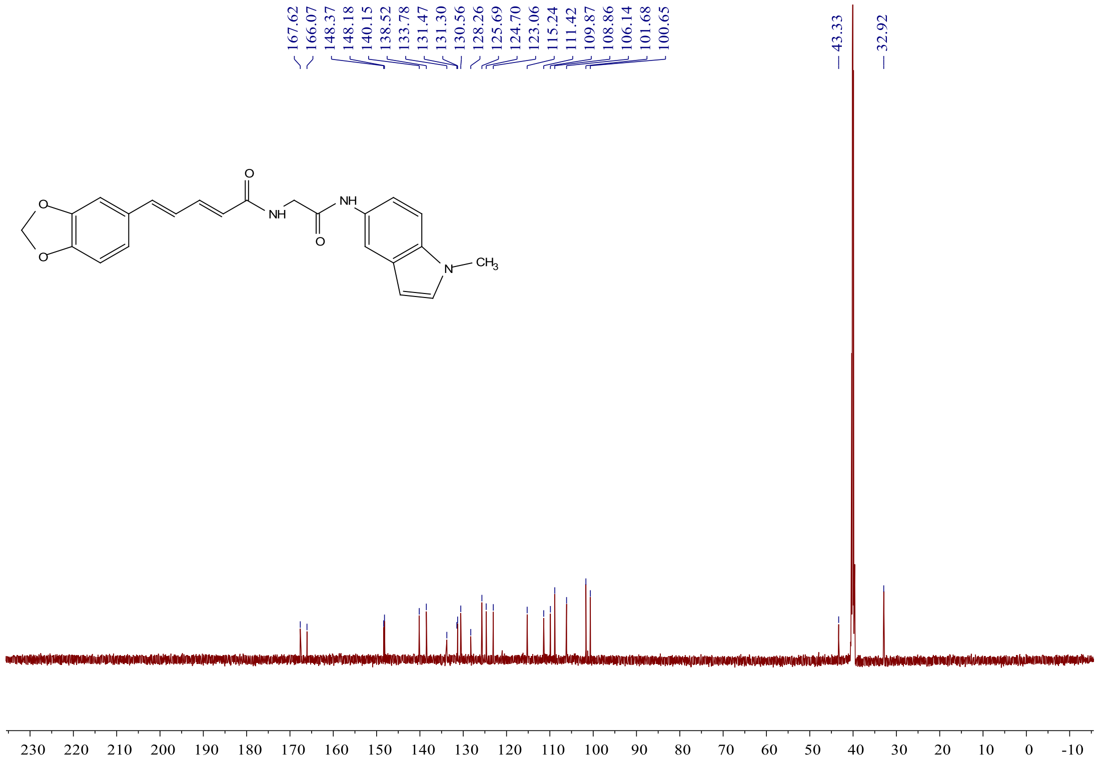
167.62 166.07 148.37 148.18 140.15 138.52 133.78 131.47 131.30 130.56 128.26 125.69 124.70 123.06 115.24 111.42 109.87 108.86 106.14 101.68 100.65
43.33
32.92
NH
NH
O
O
N
CH_3
230 220 210 200 190 180 170 160 150 140 130 120 110 100 90 80 70 60 50 40 30 20 10 0 -10

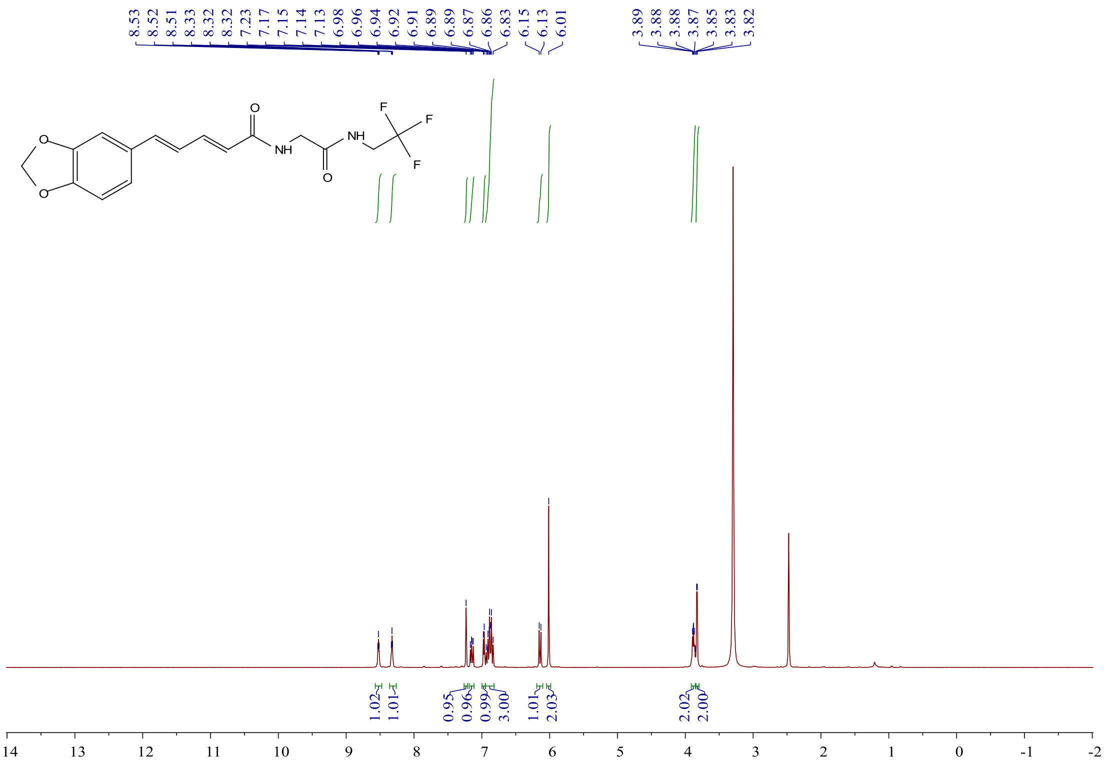
8.53
8.52
8.51
8.33
8.32
8.32
7.23
7.17
7.15
7.14
7.13
6.98
6.96
6.94
6.92
6.91
6.89
6.89
6.87
6.86
6.83
6.15
6.13
6.01
3.89
3.88
3.88
3.87
3.85
3.83
3.82
O
NH
NH
F
F
F
O
O
O
1.02
1.01
0.95
0.96
0.99
3.00
1.01
2.03
2.02
2.00
14 13 12 11 10 9 8 7 6 5 4 3 2 1 0 -1 -2

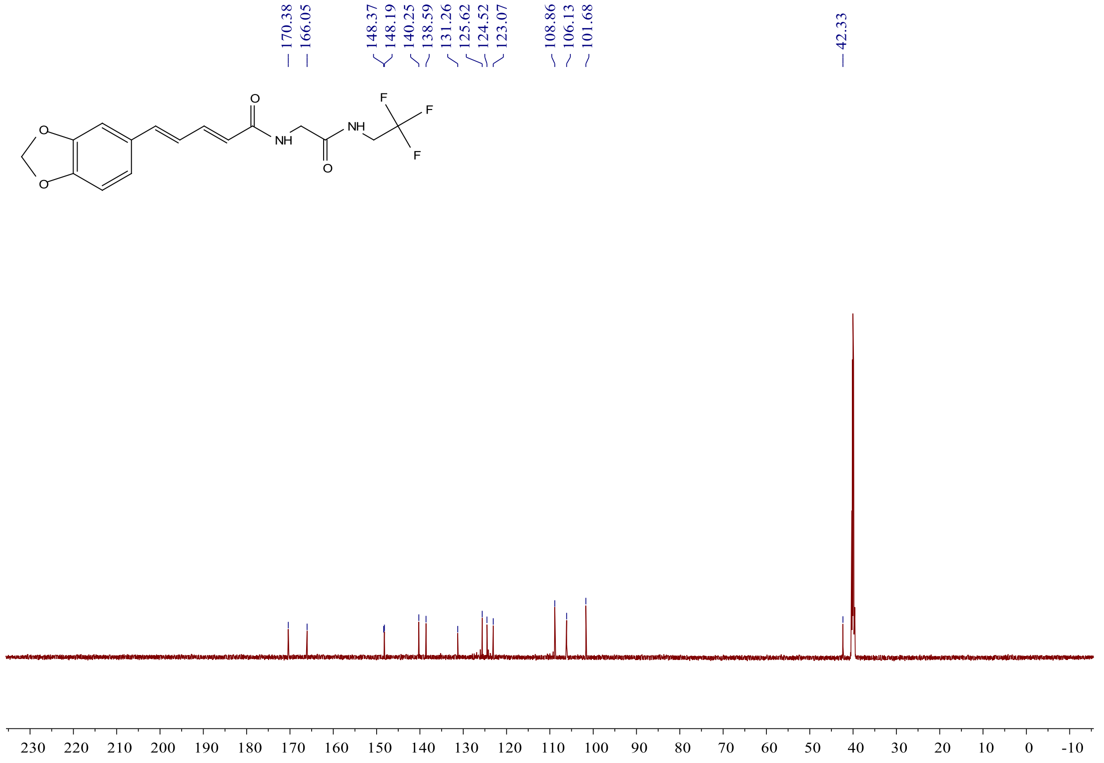
170.38
166.05
148.37
148.19
140.25
138.59
131.26
125.62
124.52
123.07
108.86
106.13
101.68
42.33
O
NH
NH
F
F
F
O
O
O
230 220 210 200 190 180 170 160 150 140 130 120 110 100 90 80 70 60 50 40 30 20 10 0 -10

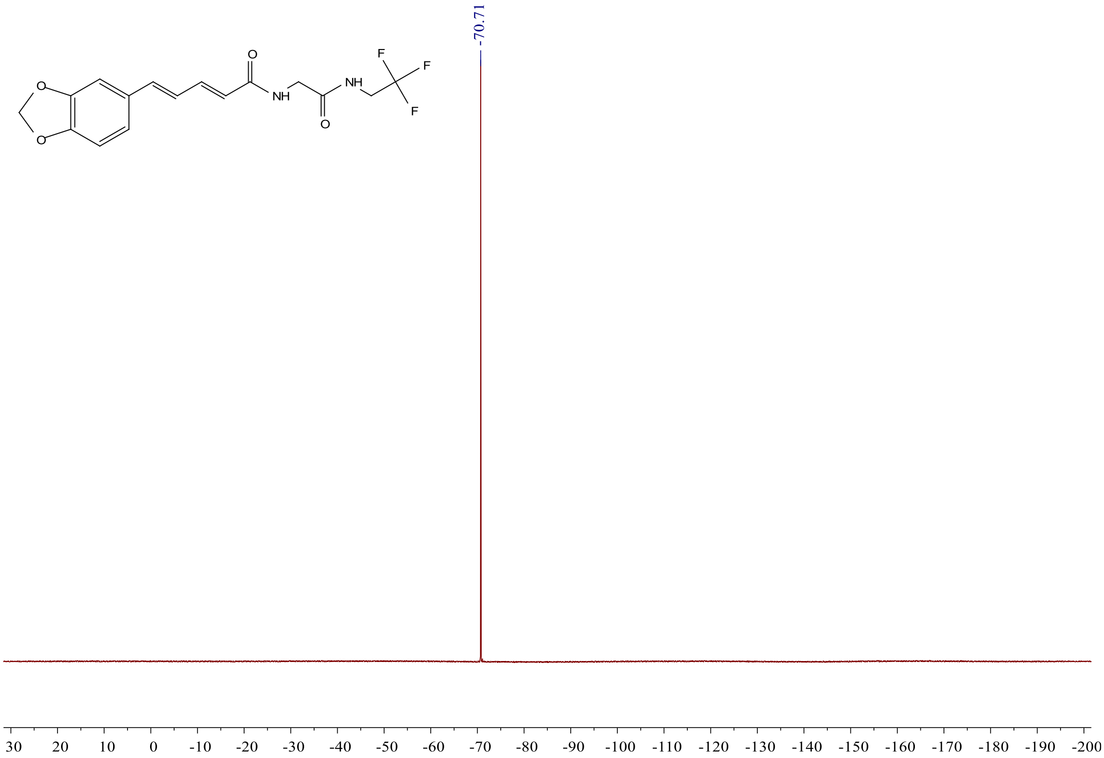
-70.71
O
O
O
NH
NH
O
F
F
F
30 20 10 0 -10 -20 -30 -40 -50 -60 -70 -80 -90 -100 -110 -120 -130 -140 -150 -160 -170 -180 -190 -200

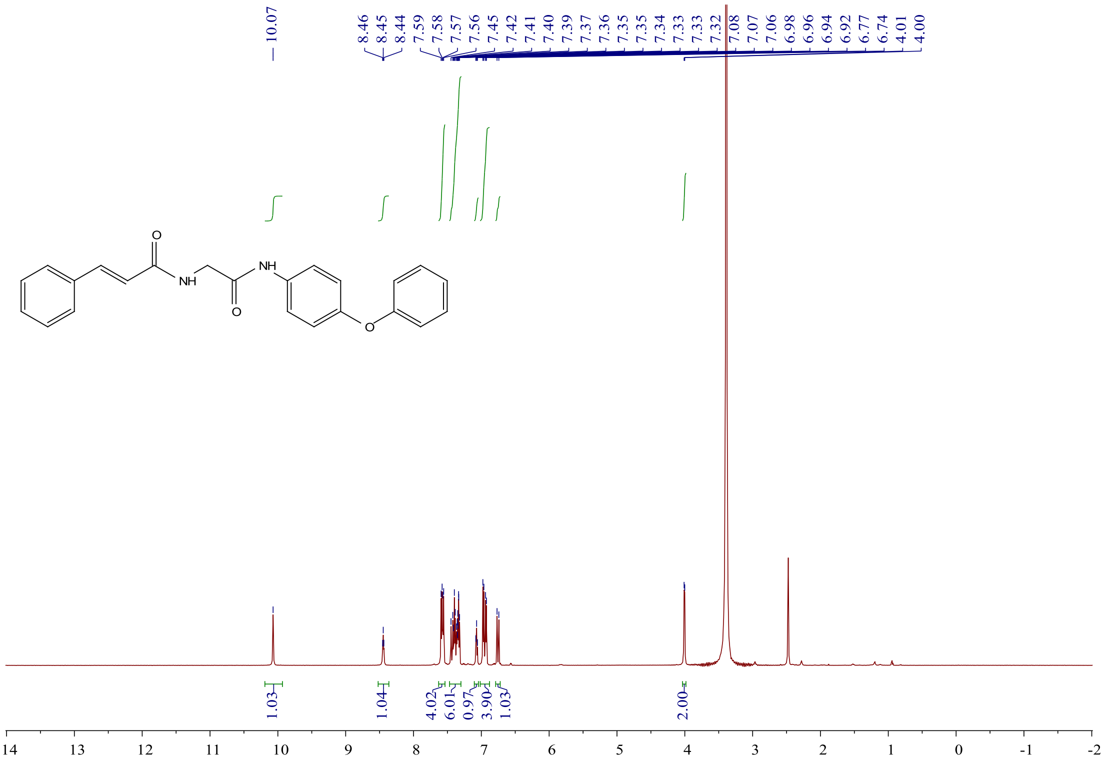
10.07
8.46 8.45 8.44 7.59 7.58 7.57 7.56 7.45 7.42 7.41 7.40 7.39 7.37 7.36 7.35 7.35 7.34 7.33 7.33 7.32 7.08 7.07 7.06 6.98 6.96 6.94 6.92 6.77 6.74 4.01 4.00
O
NH
NH
O
O
1.03
1.04
4.02
6.01
0.97
3.90
1.03
2.00
14 13 12 11 10 9 8 7 6 5 4 3 2 1 0 -1 -2

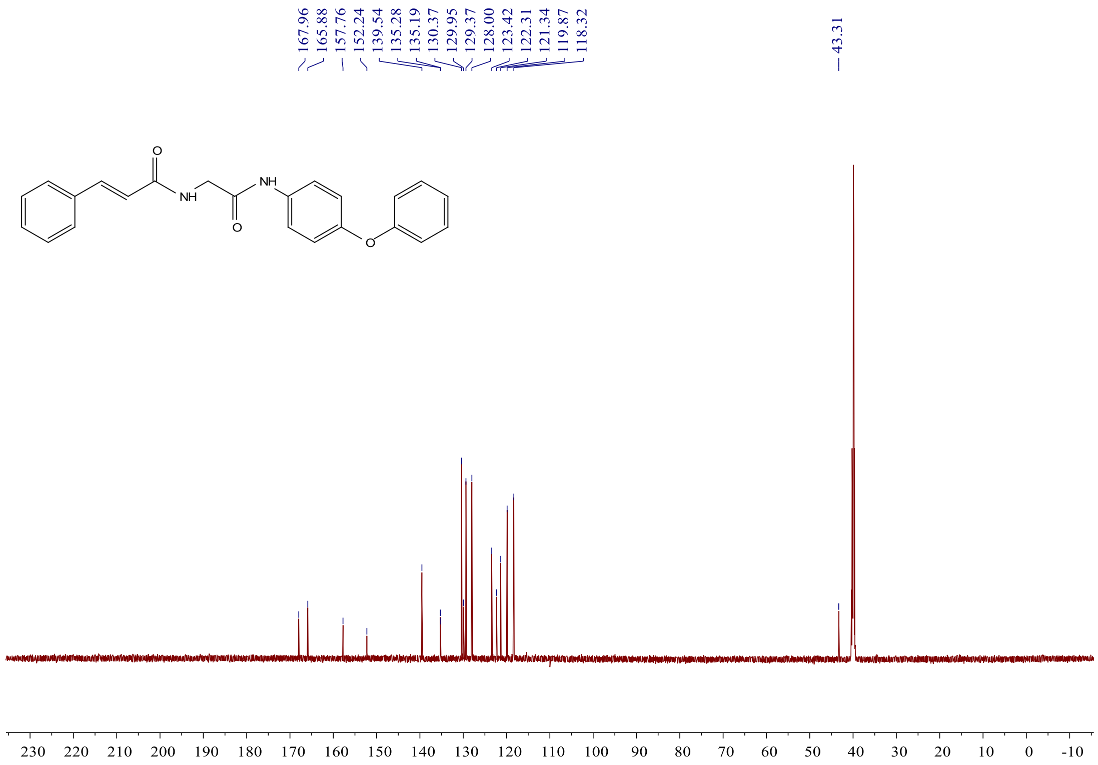
167.96
165.88
157.76
152.24
139.54
135.28
135.19
130.37
129.95
129.37
128.00
123.42
122.31
121.34
119.87
118.32
43.31
O
NH
NH
O
O
230 220 210 200 190 180 170 160 150 140 130 120 110 100 90 80 70 60 50 40 30 20 10 0 -10

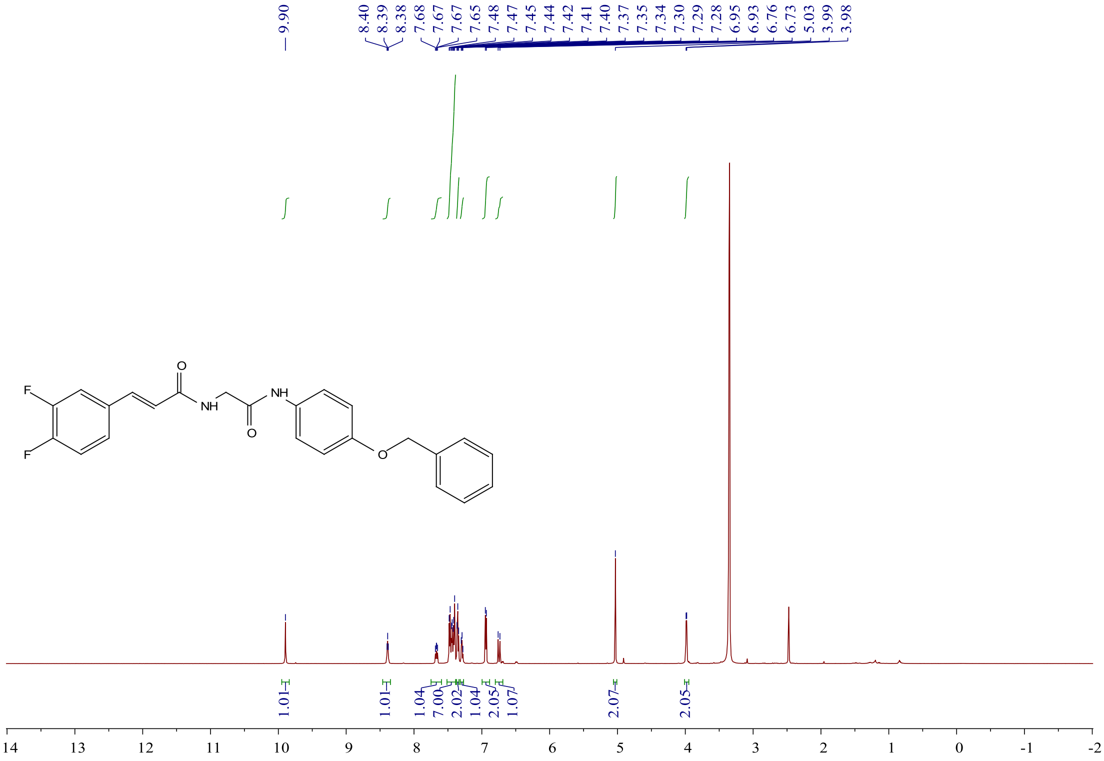
9.90
8.40
8.39
8.38
7.68
7.67
7.67
7.65
7.48
7.47
7.45
7.44
7.42
7.41
7.40
7.37
7.35
7.34
7.30
7.29
7.28
6.95
6.93
6.76
6.73
5.03
3.99
3.98
F
F
O
NH
NH
O
O
1.01
1.01
1.04
7.00
2.02
1.04
2.05
1.07
2.07
2.05
14 13 12 11 10 9 8 7 6 5 4 3 2 1 0 -1 -2

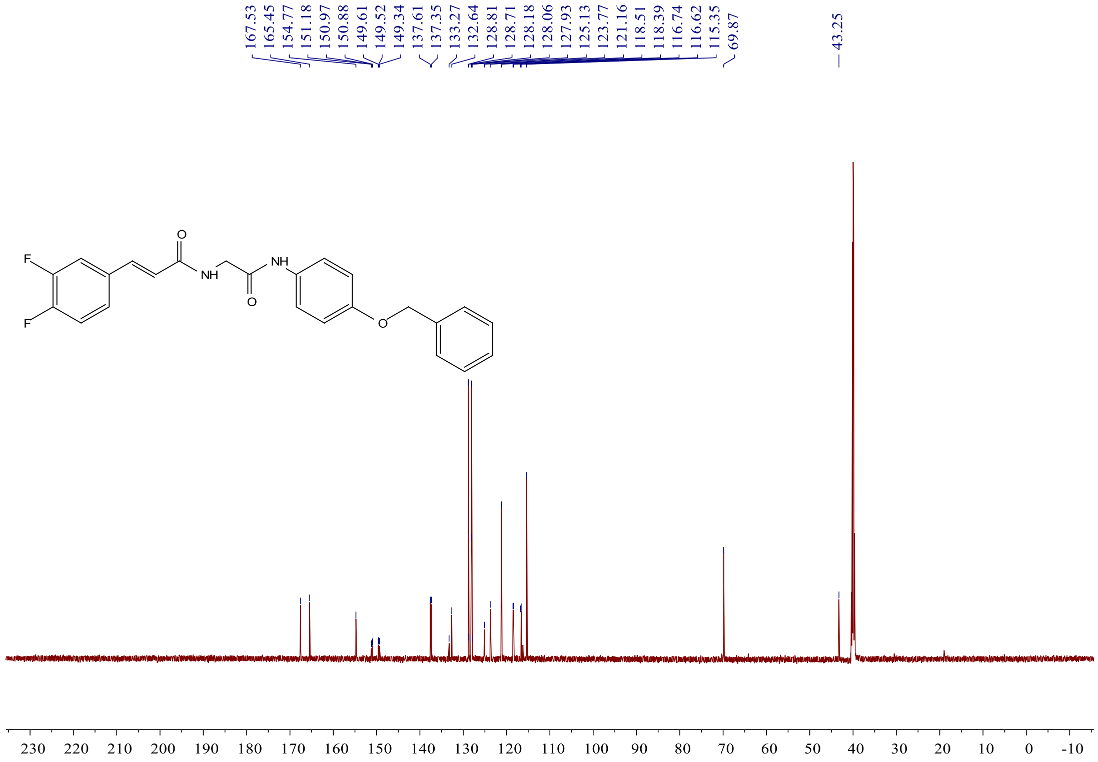
167.53
165.45
154.77
151.18
150.97
150.88
149.61
149.52
149.34
137.61
137.35
133.27
132.64
128.81
128.71
128.18
128.06
127.93
125.13
123.77
121.16
118.51
118.39
116.74
116.62
115.35
69.87
43.25
F
F
O
NH
O
NH
O
230 220 210 200 190 180 170 160 150 140 130 120 110 100 90 80 70 60 50 40 30 20 10 0 -10

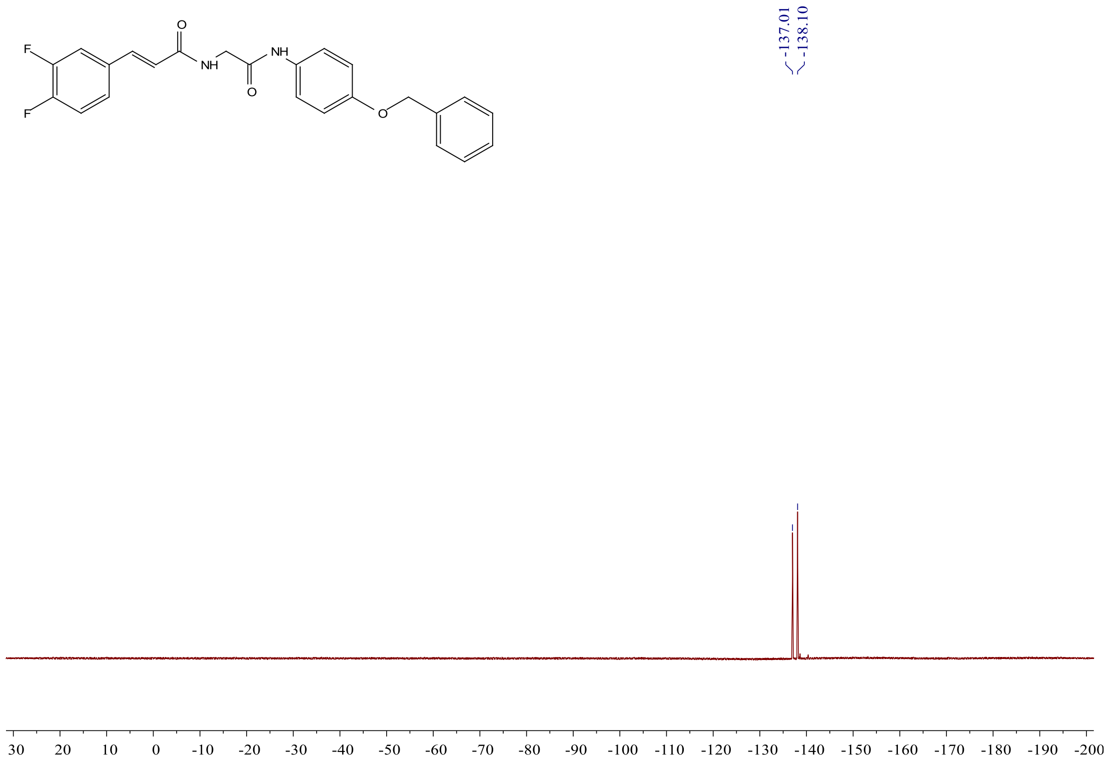
-137.01
-138.10
F
F
O
NH
O
NH
O
30 20 10 0 -10 -20 -30 -40 -50 -60 -70 -80 -90 -100 -110 -120 -130 -140 -150 -160 -170 -180 -190 -200

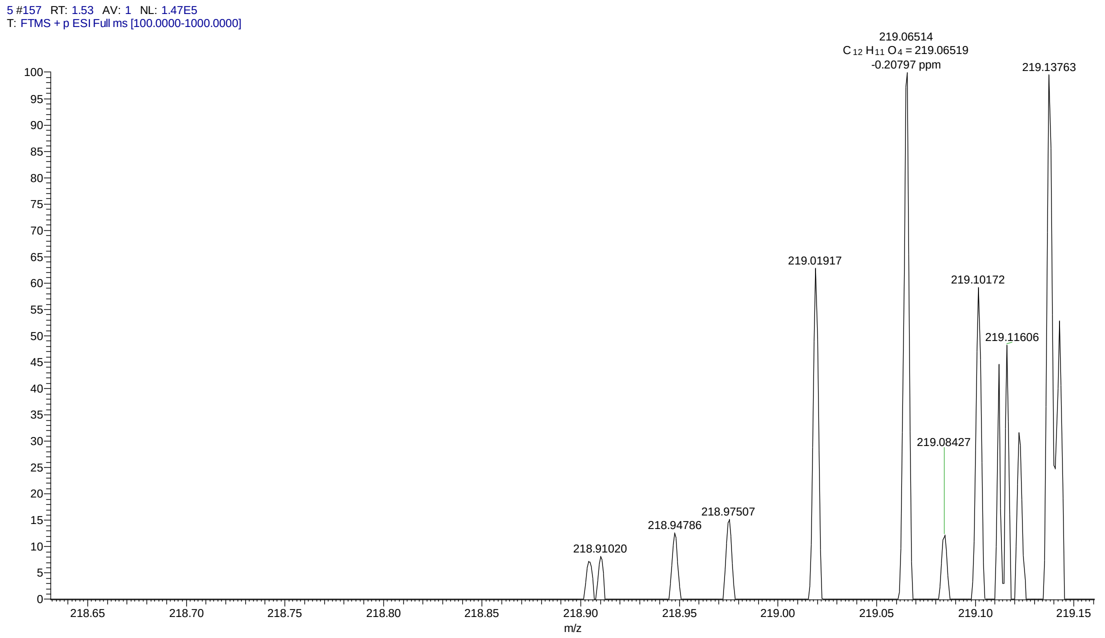

5 #157 RT: 1.53 AV: 1 NL: 1.47E5
T: FTMS + p ESI Full ms [100.0000-1000.0000]
219.06514
C 12 H 11 O 4 = 219.06519
-0.20797 ppm
219.13763
219.01917
219.10172
219.11606
219.08427
218.97507
218.94786
218.91020
m/z

C

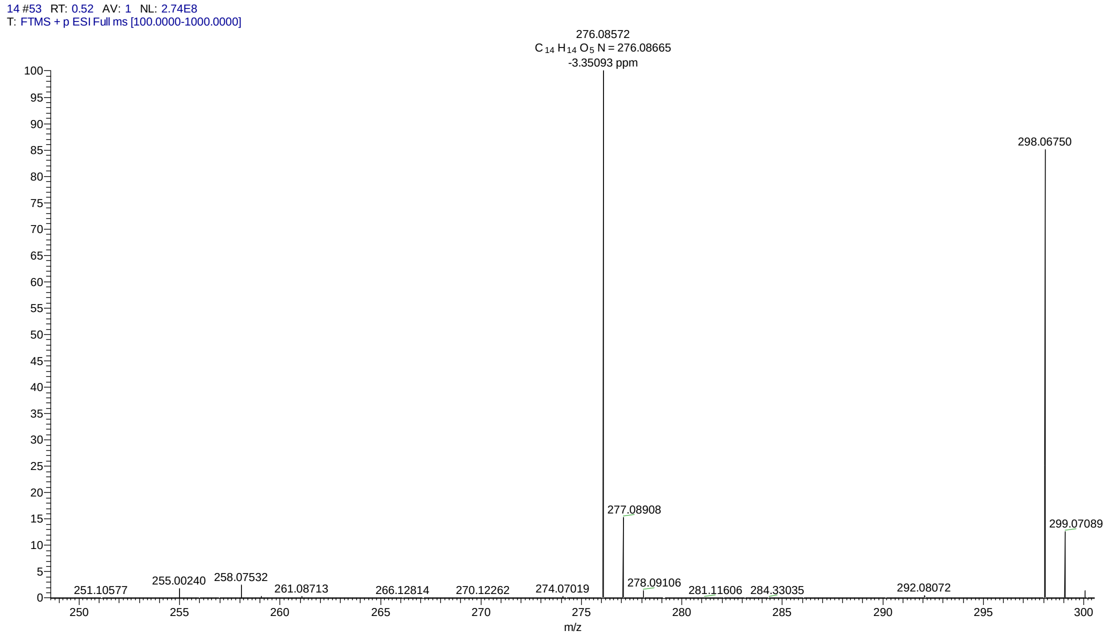

14 #53 RT: 0.52 AV: 1 NL: 2.74E8
T: FTMS + p ESI Full ms [100.0000-1000.0000]
276.08572
C 14 H 14 O 5 N = 276.08665
-3.35093 ppm
298.06750
277.08908
299.07089
251.10577
255.00240
258.07532
261.08713
266.12814
270.12262
274.07019
278.09106
281.11606
284.33035
292.08072
m/z

F_1

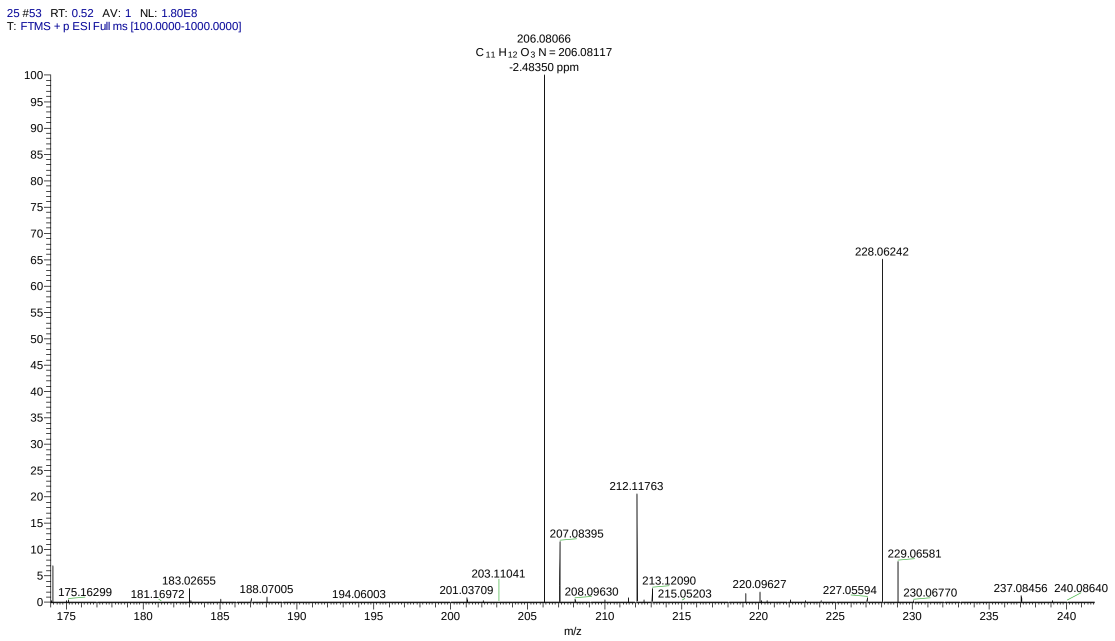
25 #53 RT: 0.52 AV: 1 NL: 1.80E8
T: FTMS + p ESI Full ms [100.0000-1000.0000]
206.08066
C 11 H 12 O 3 N = 206.08117
-2.48350 ppm
228.06242
212.11763
207.08395
229.06581
183.02655
203.11041
213.12090
175.16299
181.16972
188.07005
194.06003
201.03709
208.09630
215.05203
220.09627
227.05594
230.06770
237.08456
240.08640
m/z

F2

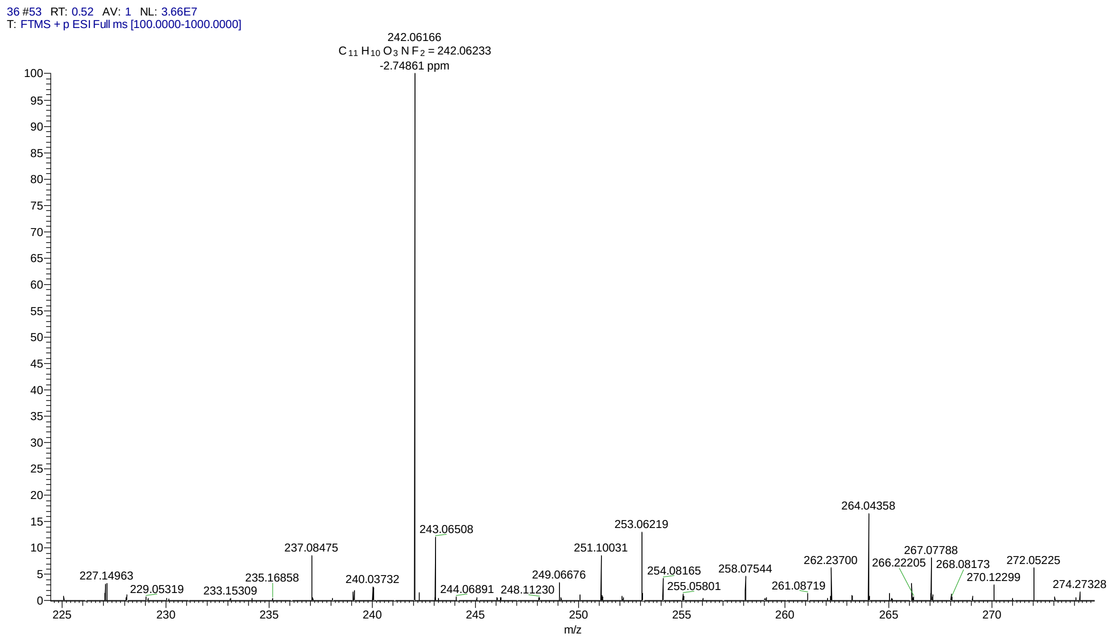
36 #53 RT: 0.52 AV: 1 NL: 3.66E7
T: FTMS + p ESI Full ms [100.0000-1000.0000]
242.06166
C 11 H 10 O 3 N F 2 = 242.06233
-2.74861 ppm
264.04358
253.06219
243.06508
237.08475
251.10031
267.07788
262.23700
272.05225
227.14963
235.16858
240.03732
249.06676
254.08165
258.07544
266.22205
268.08173
229.05319
233.15309
244.06891
248.11230
255.05801
261.08719
270.12299
274.27328
m/z

D1

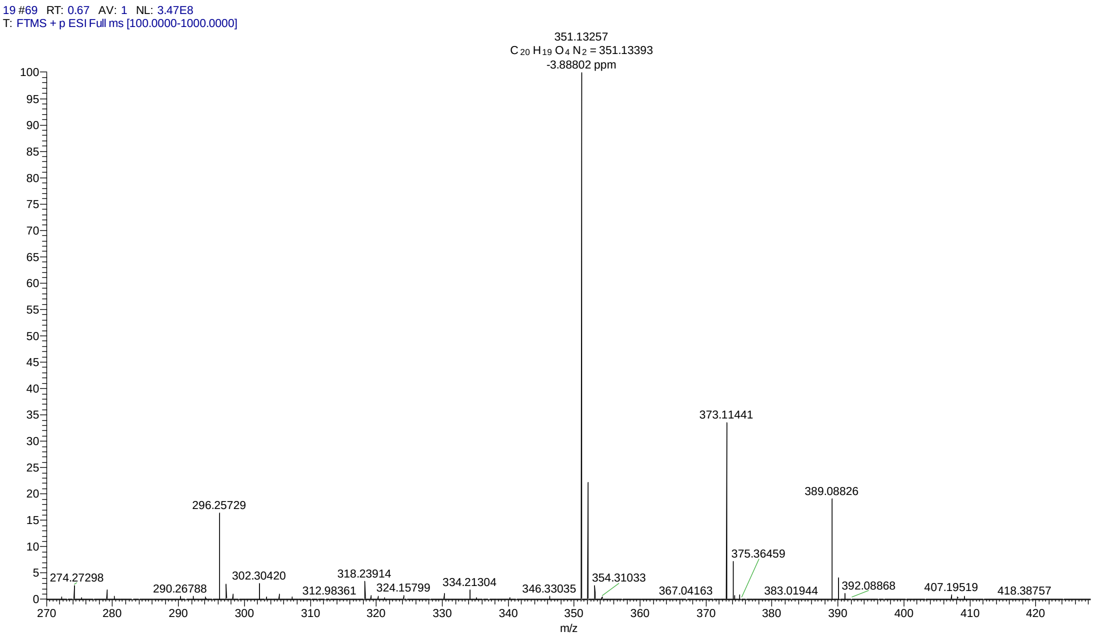
19 #69 RT: 0.67 AV: 1 NL: 3.47E8
T: FTMS + p ESI Full ms [100.0000-1000.0000]
351.13257
C 20 H 19 O 4 N 2 = 351.13393
-3.88802 ppm
373.11441
296.25729
389.08826
375.36459
274.27298
290.26788
302.30420
312.98361
318.23914
324.15799
334.21304
346.33035
354.31033
367.04163
383.01944
392.08868
407.19519
418.38757
m/z

D2

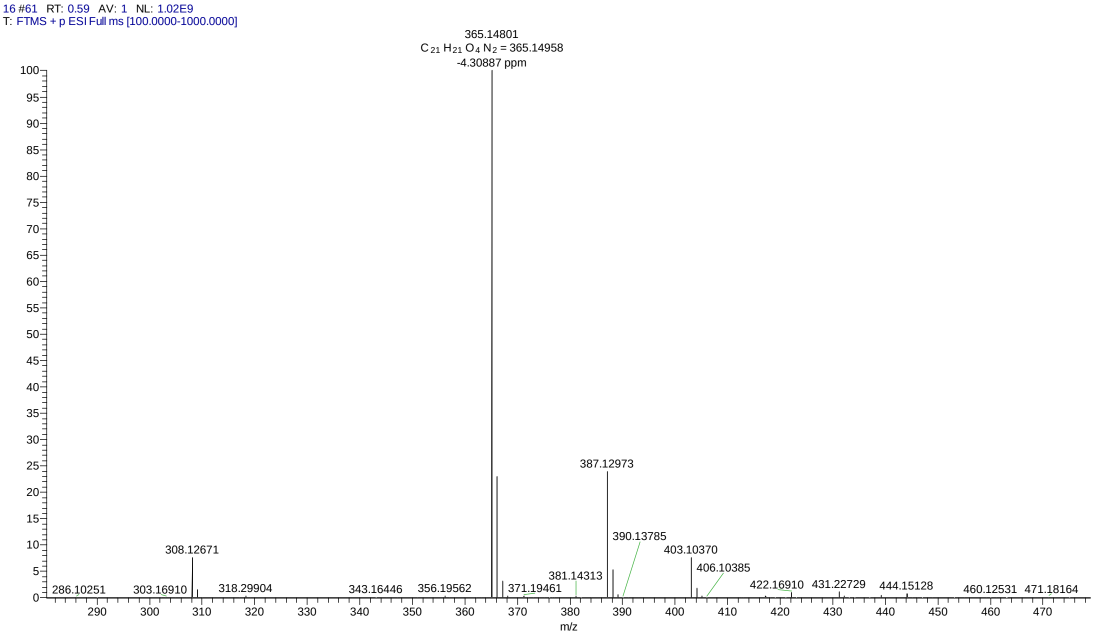
16 #61 RT: 0.59 AV: 1 NL: 1.02E9
T: FTMS + p ESI Full ms [100.0000-1000.0000]
365.14801
C 21 H 21 O 4 N 2 = 365.14958
-4.30887 ppm
387.12973
308.12671
390.13785
403.10370
406.10385
381.14313
286.10251
303.16910
318.29904
343.16446
356.19562
371.19461
422.16910
431.22729
444.15128
460.12531
471.18164
m/z

D3

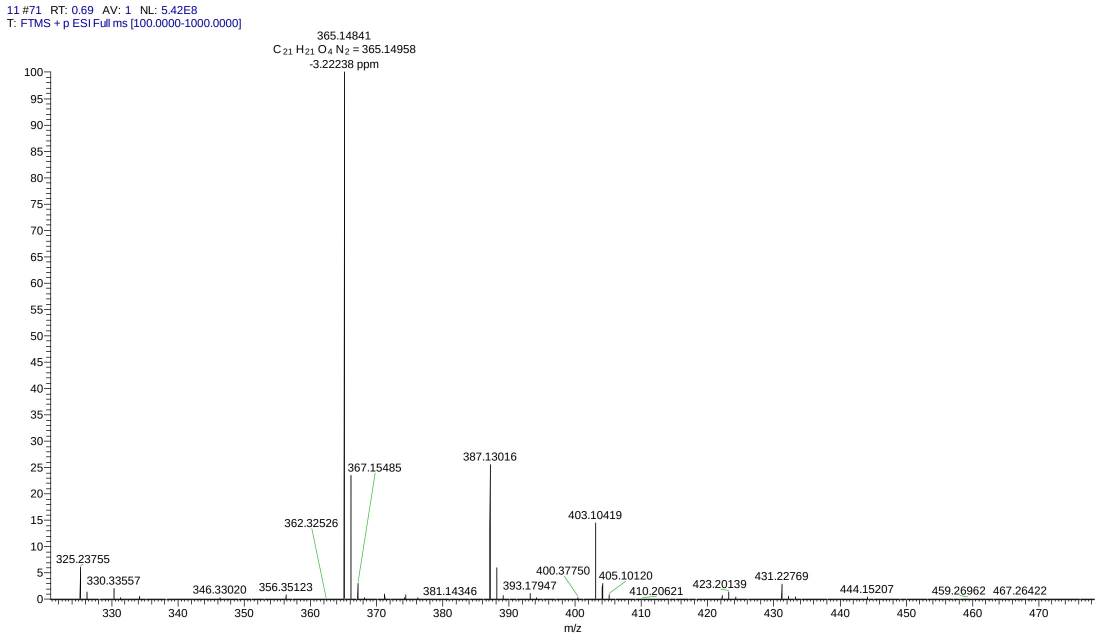
11 #71 RT: 0.69 AV: 1 NL: 5.42E8
T: FTMS + p ESI Full ms [100.0000-1000.0000]
365.14841
$C_{21}H_{21}O_4N_2$ = 365.14958
-3.22238 ppm
367.15485
387.13016
362.32526
403.10419
325.23755
330.33557
346.33020
356.35123
381.14346
393.17947
400.37750
405.10120
410.20621
423.20139
431.22769
444.15207
459.26962
467.26422
m/z

D4

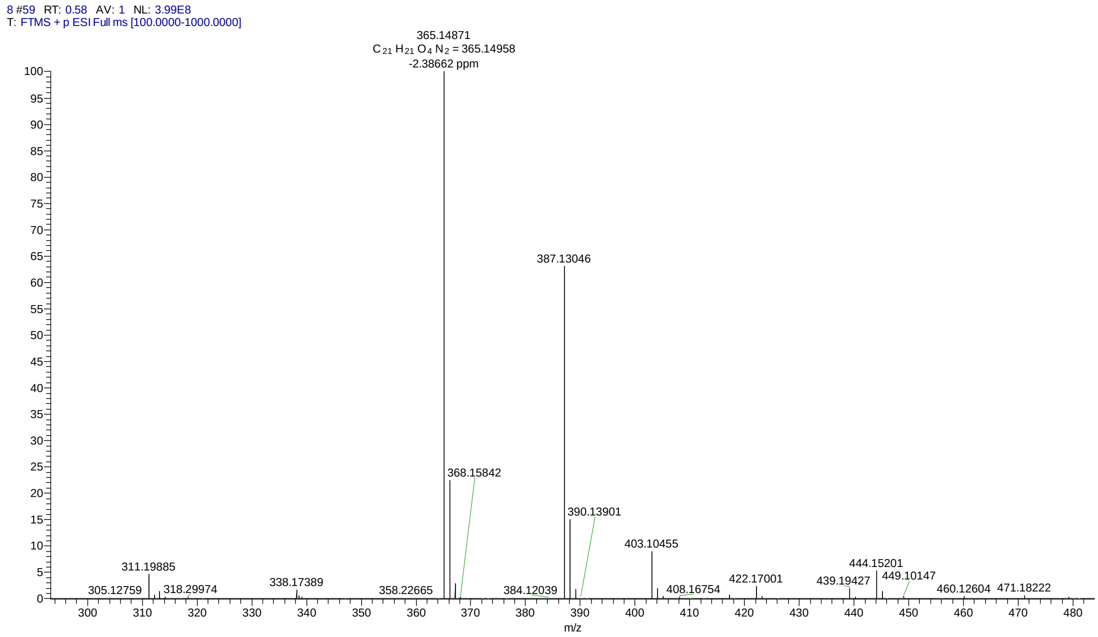
8 #59 RT: 0.58 AV: 1 NL: 3.99E8
T: FTMS + p ESI Full ms [100.0000-1000.0000]
365.14871
$C_{21}H_{21}O_4N_2$ = 365.14958
-2.38662 ppm
387.13046
368.15842
390.13901
403.10455
311.19885
305.12759
318.29974
338.17389
358.22665
384.12039
408.16754
422.17001
439.19427
444.15201
449.10147
460.12604
471.18222
m/z

D5

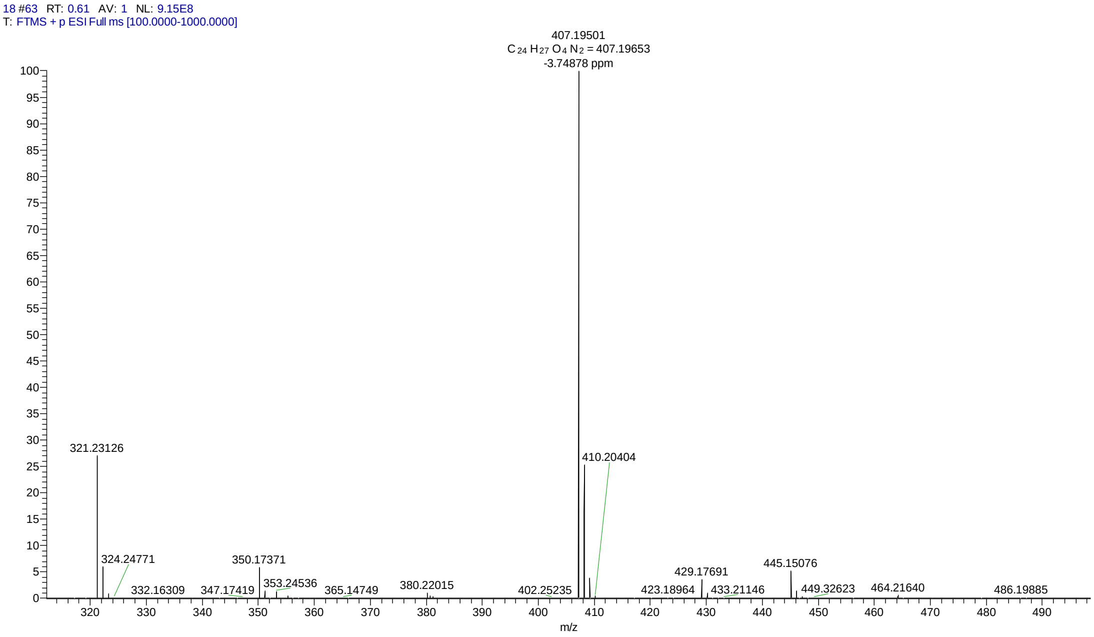
18 #63 RT: 0.61 AV: 1 NL: 9.15E8
T: FTMS + p ESI Full ms [100.0000-1000.0000]
407.19501
C 24 H 27 O 4 N 2 = 407.19653
-3.74878 ppm
321.23126
410.20404
324.24771
350.17371
429.17691
445.15076
332.16309
347.17419
353.24536
365.14749
380.22015
402.25235
423.18964
433.21146
449.32623
464.21640
486.19885
m/z

D6

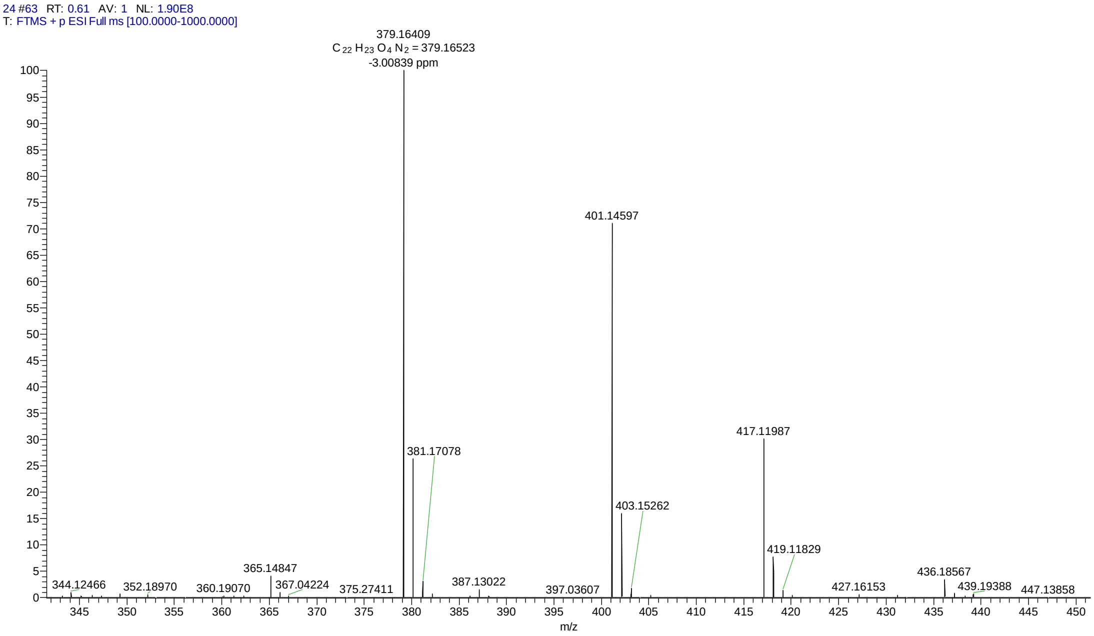
24 #63 RT: 0.61 AV: 1 NL: 1.90E8
T: FTMS + p ESI Full ms [100.0000-1000.0000]
379.16409
C 22 H 23 O 4 N 2 = 379.16523
-3.00839 ppm
401.14597
417.11987
381.17078
403.15262
419.11829
365.14847
387.13022
436.18567
344.12466
352.18970
360.19070
367.04224
375.27411
397.03607
427.16153
439.19388
447.13858
m/z

D7

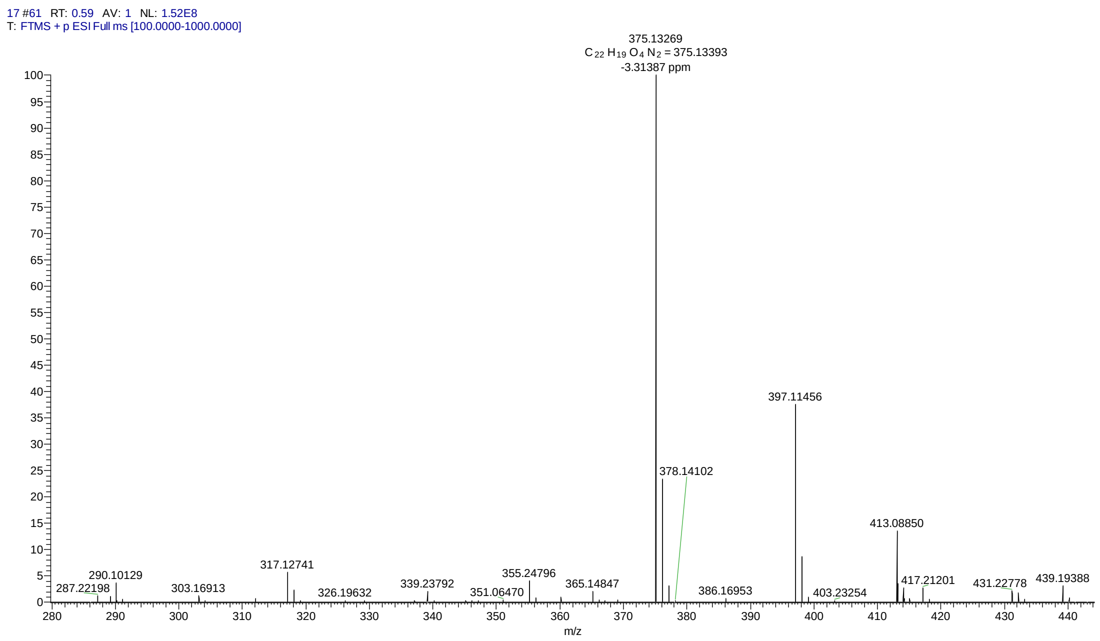

17 #61 RT: 0.59 AV: 1 NL: 1.52E8
T: FTMS + p ESI Full ms [100.0000-1000.0000]
375.13269
C 22 H19 O 4 N 2 = 375.13393
-3.31387 ppm
397.11456
378.14102
413.08850
317.12741
290.10129
287.22198
303.16913
326.19632
339.23792
351.06470
355.24796
365.14847
386.16953
403.23254
417.21201
431.22778
439.19388
m/z

D8

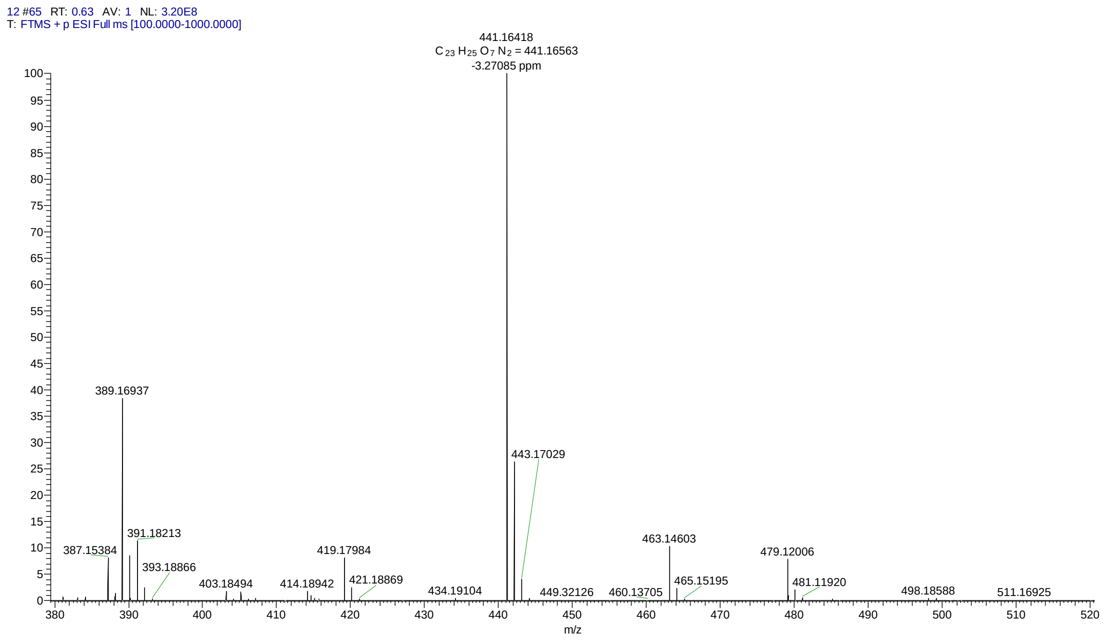

12 #65 RT: 0.63 AV: 1 NL: 3.20E8
T: FTMS + p ESI Full ms [100.0000-1000.0000]
441.16418
C 23 H25 O 7 N 2 = 441.16563
-3.27085 ppm
389.16937
443.17029
391.18213
387.15384
393.18866
419.17984
403.18494
414.18942
421.18869
434.19104
449.32126
460.13705
463.14603
465.15195
479.12006
481.11920
498.18588
511.16925
m/z

D9

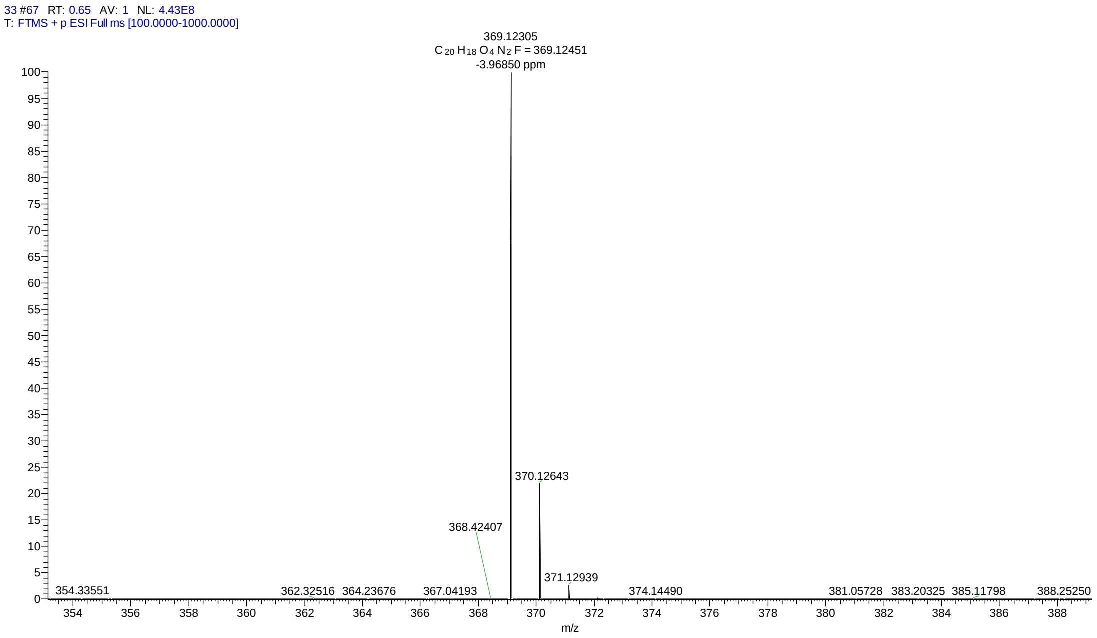
33 #67 RT: 0.65 AV: 1 NL: 4.43E8
T: FTMS + p ESI Full ms [100.0000-1000.0000]
369.12305
C 20 H18 O4 N2 F = 369.12451
-3.96850 ppm
370.12643
368.42407
371.12939
354.33551
362.32516
364.23676
367.04193
374.14490
381.05728
383.20325
385.11798
388.25250
m/z

D_{10}

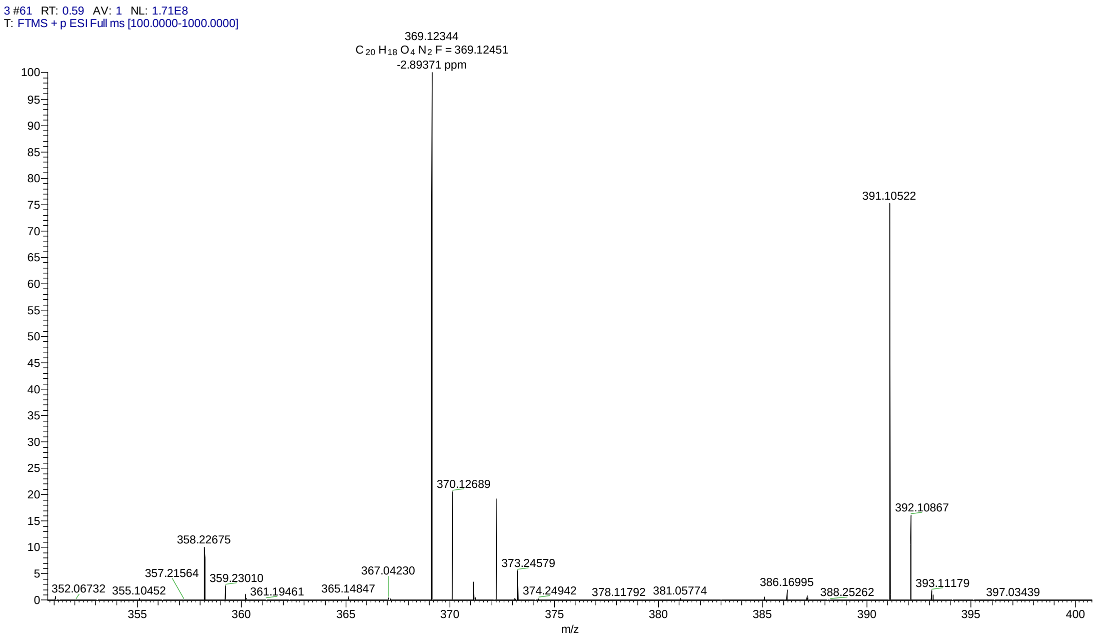
3 #61 RT: 0.59 AV: 1 NL: 1.71E8
T: FTMS + p ESI Full ms [100.0000-1000.0000]
369.12344
C 20 H18 O4 N2 F = 369.12451
-2.89371 ppm
391.10522
370.12689
392.10867
358.22675
373.24579
357.21564
359.23010
367.04230
352.06732
355.10452
361.19461
365.14847
374.24942
378.11792
381.05774
386.16995
388.25262
393.11179
397.03439
m/z

D_{11}

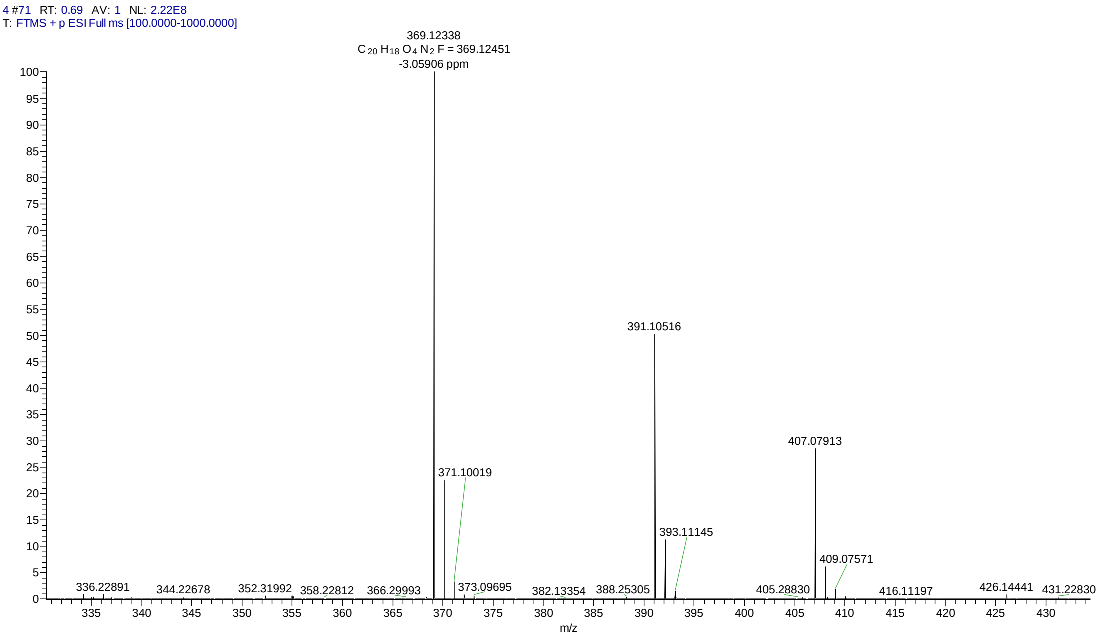
4 #71 RT: 0.69 AV: 1 NL: 2.22E8
T: FTMS + p ESI Full ms [100.0000-1000.0000]
369.12338
C 20 H 18 O 4 N 2 F = 369.12451
-3.05906 ppm
391.10516
407.07913
371.10019
393.11145
409.07571
336.22891
344.22678
352.31992
358.22812
366.29993
373.09695
382.13354
388.25305
405.28830
416.11197
426.14441
431.22830
m/z

D12

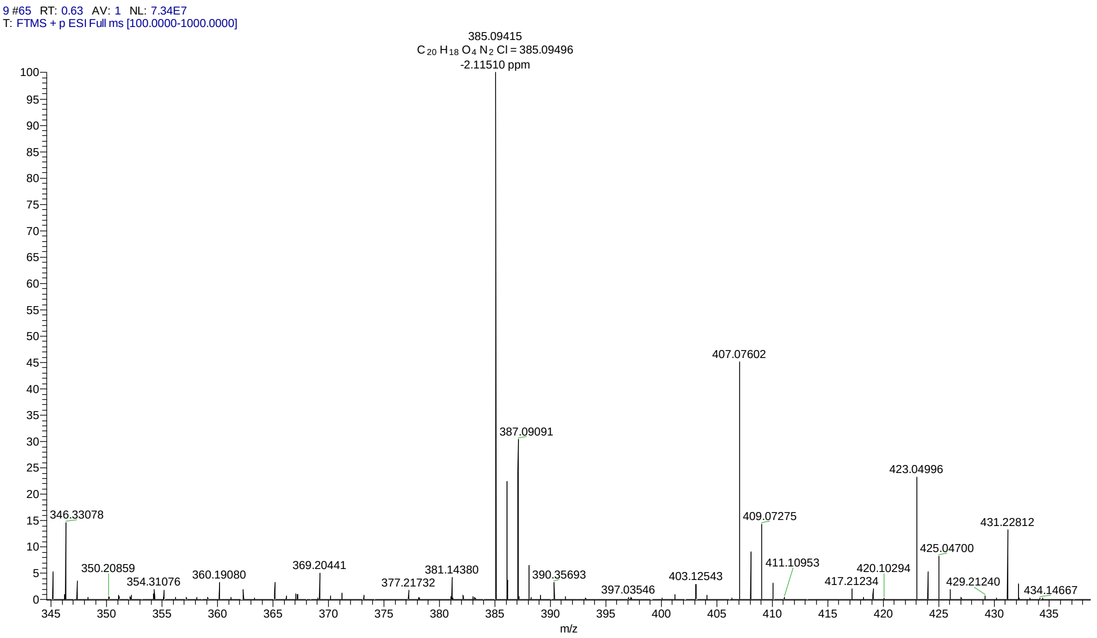
9 #65 RT: 0.63 AV: 1 NL: 7.34E7
T: FTMS + p ESI Full ms [100.0000-1000.0000]
385.09415
C 20 H 18 O 4 N 2 Cl = 385.09496
-2.11510 ppm
407.07602
387.09091
423.04996
346.33078
409.07275
431.22812
425.04700
350.20859
354.31076
360.19080
369.20441
377.21732
381.14380
390.35693
397.03546
403.12543
411.10953
417.21234
420.10294
429.21240
434.14667
m/z

D13

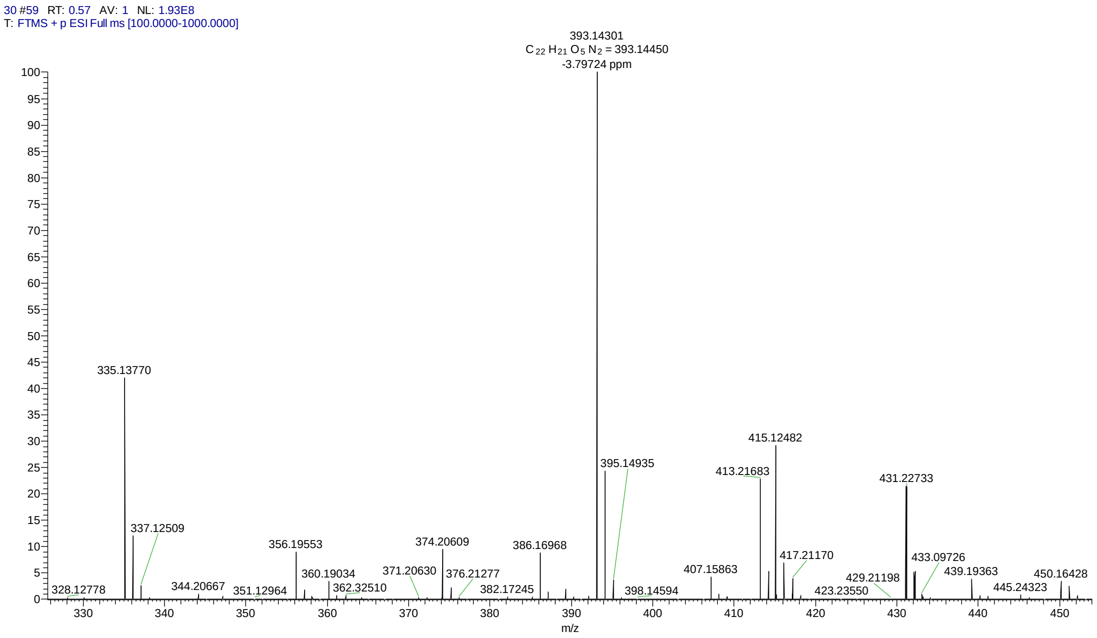
30 #59 RT: 0.57 AV: 1 NL: 1.93E8
T: FTMS + p ESI Full ms [100.0000-1000.0000]
393.14301
C 22 H 21 O 5 N 2 = 393.14450
-3.79724 ppm
335.13770
337.12509
328.12778
344.20667
351.12964
356.19553
360.19034
362.32510
371.20630
374.20609
376.21277
382.17245
386.16968
395.14935
398.14594
407.15863
413.21683
415.12482
417.21170
423.23550
429.21198
431.22733
433.09726
439.19363
445.24323
450.16428
m/z

D14

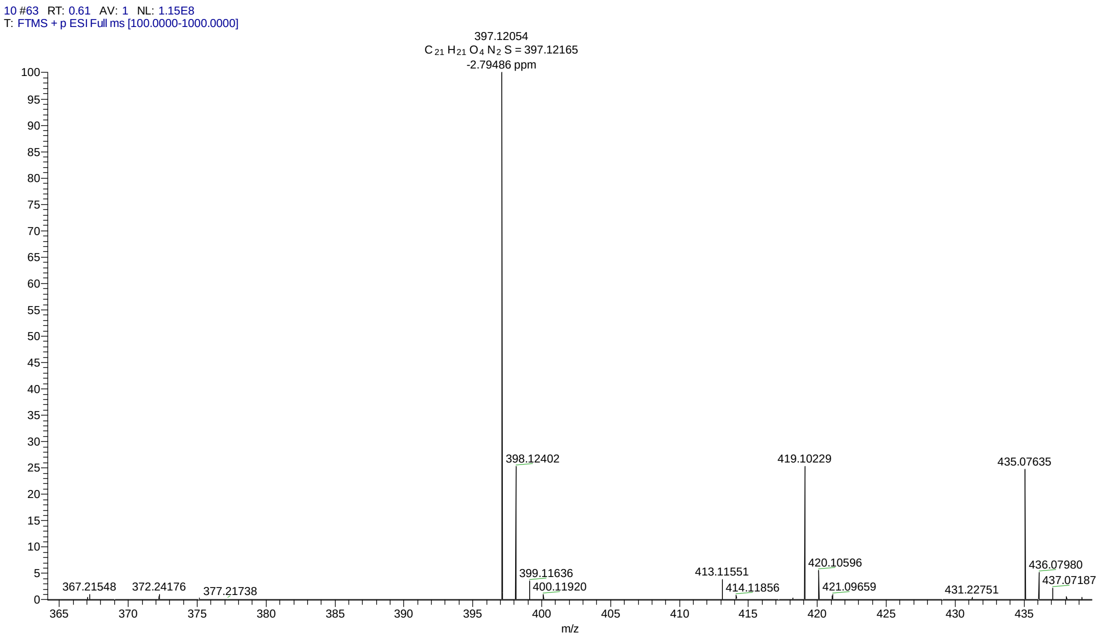
10 #63 RT: 0.61 AV: 1 NL: 1.15E8
T: FTMS + p ESI Full ms [100.0000-1000.0000]
397.12054
C 21 H 21 O 4 N 2 S = 397.12165
-2.79486 ppm
398.12402
399.11636
400.11920
367.21548
372.24176
377.21738
413.11551
414.11856
419.10229
420.10596
421.09659
431.22751
435.07635
436.07980
437.07187
m/z

D15

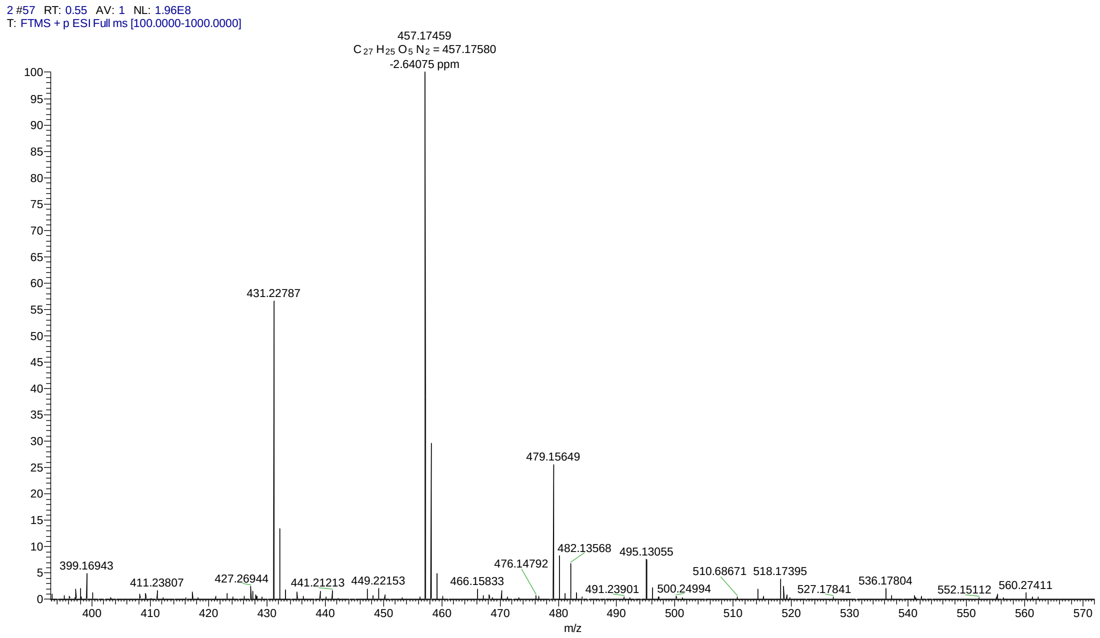
2 #57 RT: 0.55 AV: 1 NL: 1.96E8
T: FTMS + p ESI Full ms [100.0000-1000.0000]
457.17459
C 27 H25 O5 N2 = 457.17580
-2.64075 ppm
431.22787
479.15649
399.16943
411.23807
427.26944
441.21213
449.22153
466.15833
476.14792
482.13568
495.13055
491.23901
500.24994
510.68671
518.17395
527.17841
536.17804
552.15112
560.27411
m/z

D16

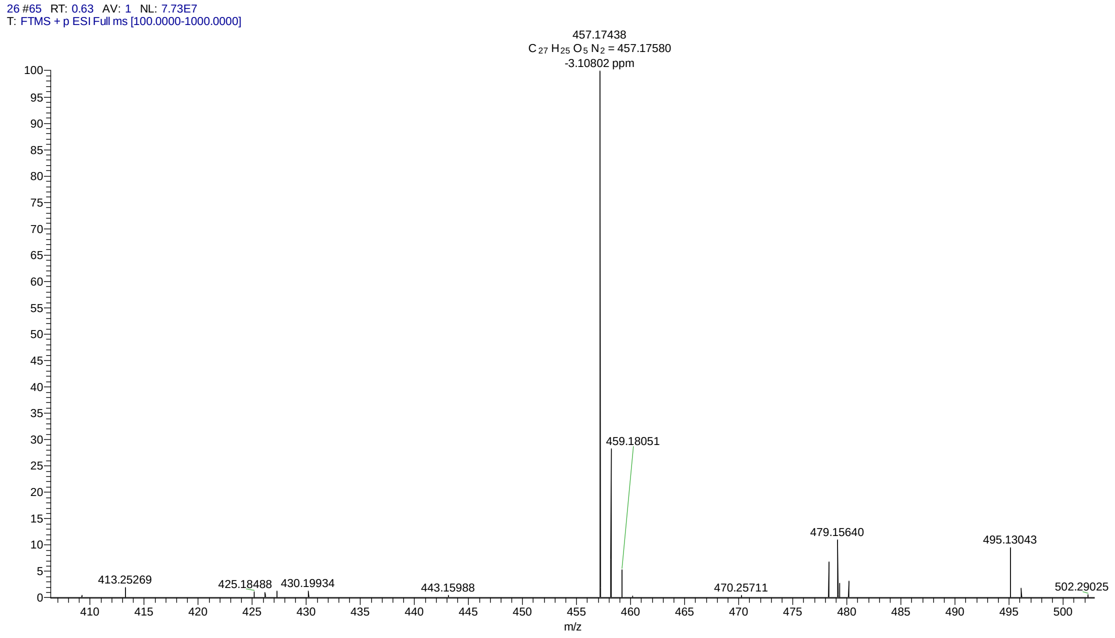
26 #65 RT: 0.63 AV: 1 NL: 7.73E7
T: FTMS + p ESI Full ms [100.0000-1000.0000]
457.17438
C 27 H25 O5 N2 = 457.17580
-3.10802 ppm
459.18051
479.15640
495.13043
413.25269
425.18488
430.19934
443.15988
470.25711
502.29025
m/z

D17

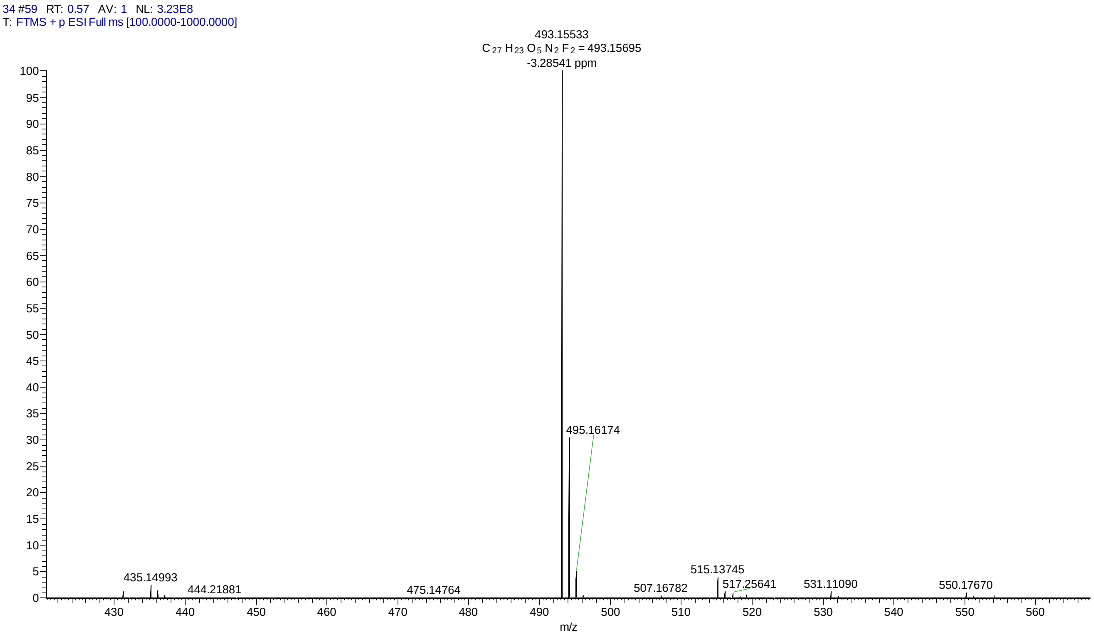
34 #59 RT: 0.57 AV: 1 NL: 3.23E8
T: FTMS + p ESI Full ms [100.0000-1000.0000]
493.15533
C 27 H 23 O 5 N 2 F 2 = 493.15695
-3.28541 ppm
495.16174
435.14993
444.21881
475.14764
507.16782
515.13745
517.25641
531.11090
550.17670
m/z

D18

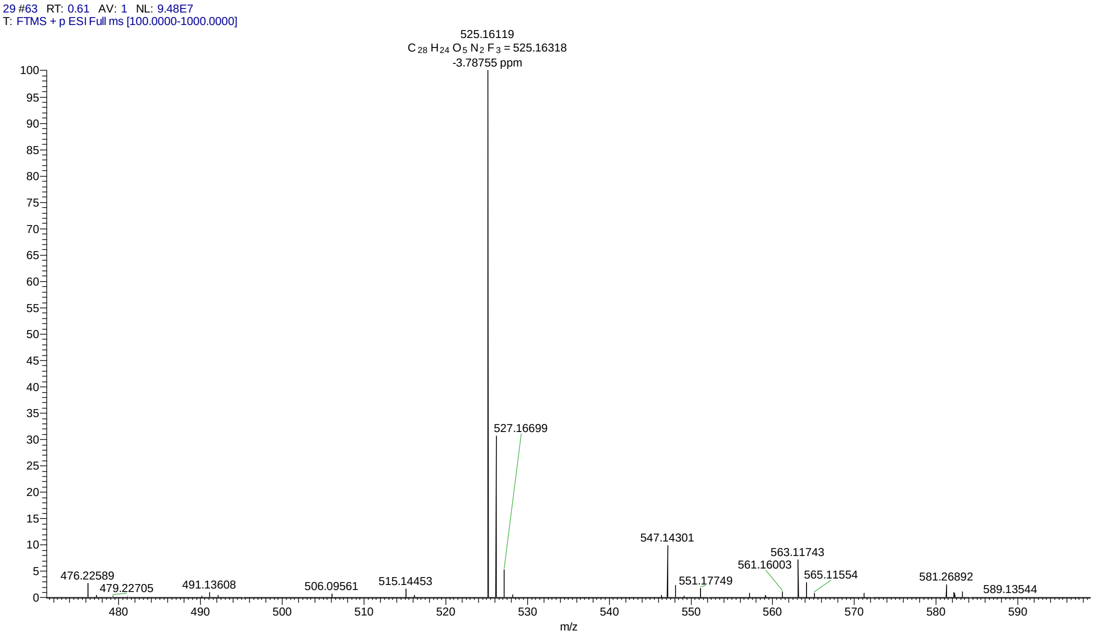
29 #63 RT: 0.61 AV: 1 NL: 9.48E7
T: FTMS + p ESI Full ms [100.0000-1000.0000]
525.16119
C 28 H 24 O 5 N 2 F 3 = 525.16318
-3.78755 ppm
527.16699
547.14301
563.11743
561.16003
565.11554
551.17749
581.26892
589.13544
476.22589
479.22705
491.13608
506.09561
515.14453
m/z

D19

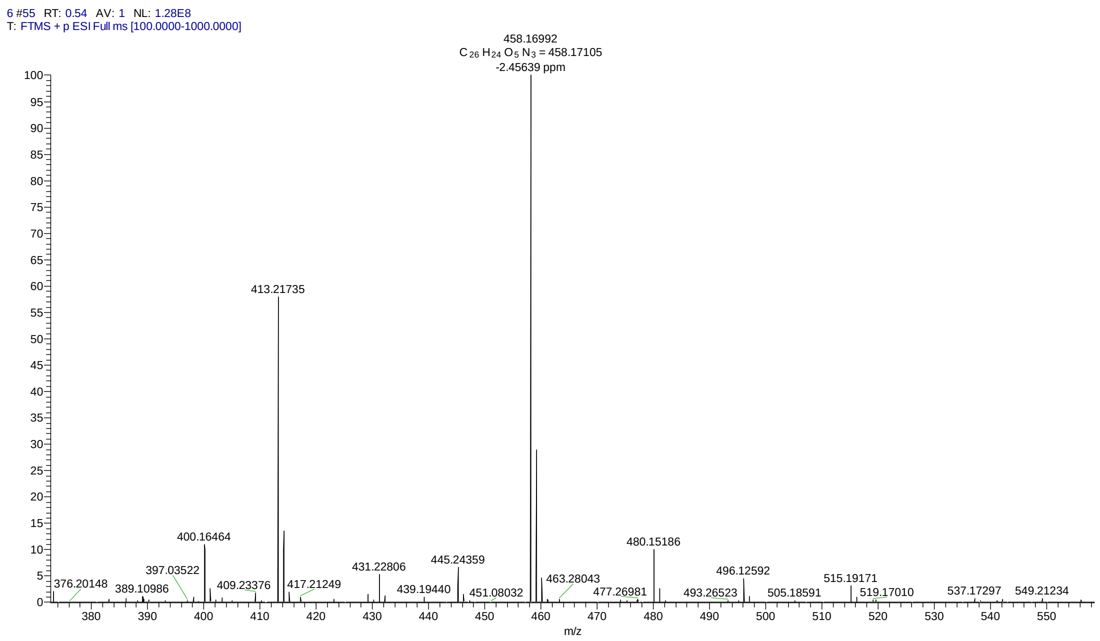
6 #55 RT: 0.54 AV: 1 NL: 1.28E8
T: FTMS + p ESI Full ms [100.0000-1000.0000]
458.16992
C 26 H 24 O 5 N 3 = 458.17105
-2.45639 ppm
413.21735
400.16464
480.15186
397.03522
431.22806
445.24359
496.12592
376.20148
389.10986
409.23376
417.21249
439.19440
451.08032
463.28043
477.26981
493.26523
505.18591
515.19171
519.17010
537.17297
549.21234
m/z

D20

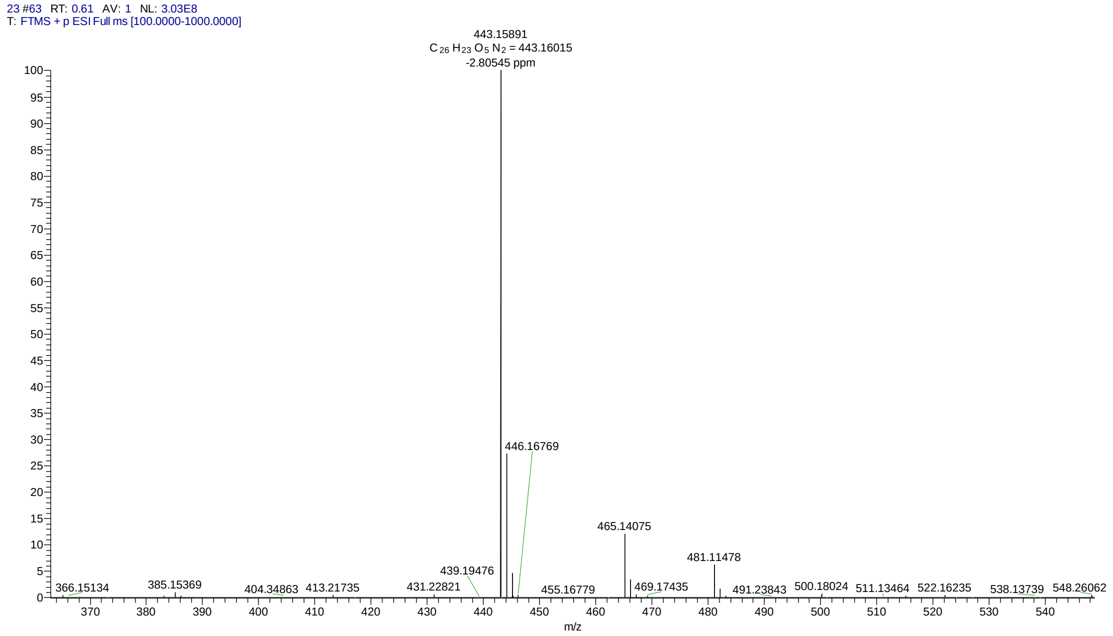
23 #63 RT: 0.61 AV: 1 NL: 3.03E8
T: FTMS + p ESI Full ms [100.0000-1000.0000]
443.15891
C 26 H 23 O 5 N 2 = 443.16015
-2.80545 ppm
446.16769
465.14075
481.11478
439.19476
366.15134
385.15369
404.34863
413.21735
431.22821
455.16779
469.17435
491.23843
500.18024
511.13464
522.16235
538.13739
548.26062
m/z

D21

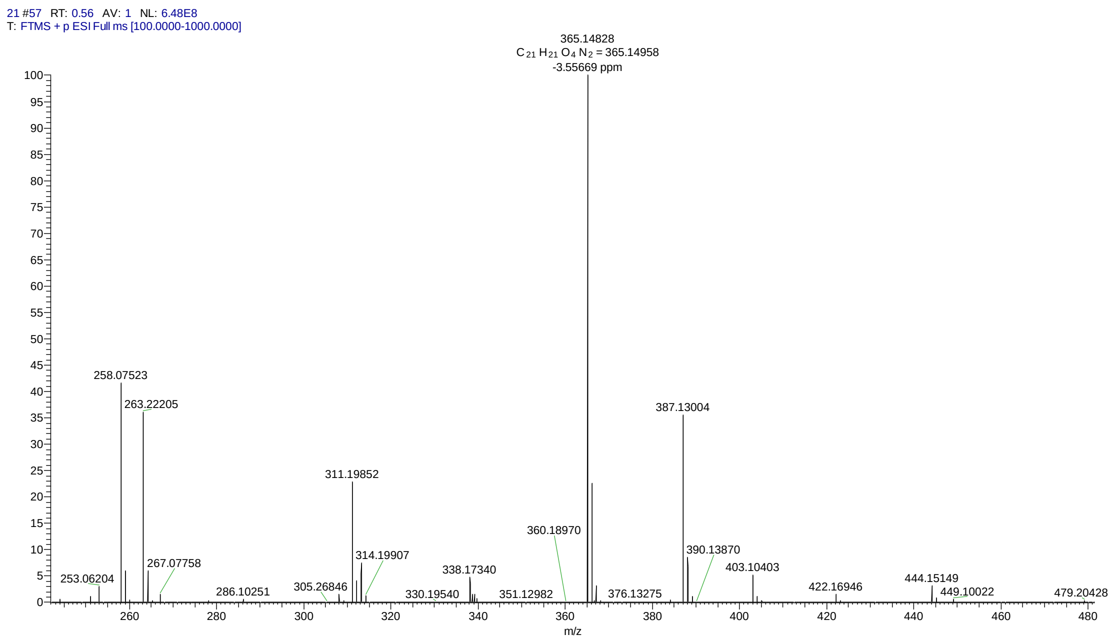
21 #57 RT: 0.56 AV: 1 NL: 6.48E8
T: FTMS + p ESI Full ms [100.0000-1000.0000]
365.14828
C 21 H 21 O 4 N 2 = 365.14958
-3.55669 ppm
258.07523
263.22205
387.13004
311.19852
360.18970
390.13870
314.19907
267.07758
338.17340
403.10403
253.06204
444.15149
286.10251
305.26846
422.16946
449.10022
479.20428
330.19540
351.12982
376.13275
m/z

D22

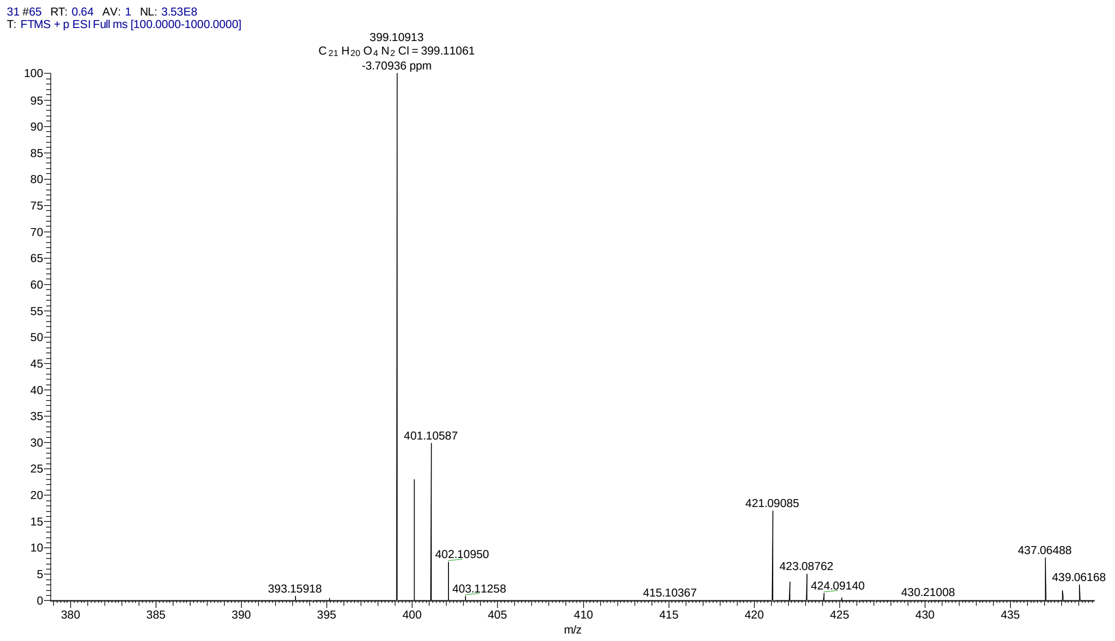
31 #65 RT: 0.64 AV: 1 NL: 3.53E8
T: FTMS + p ESI Full ms [100.0000-1000.0000]
399.10913
C 21 H 20 O 4 N 2 Cl = 399.11061
-3.70936 ppm
401.10587
421.09085
402.10950
437.06488
423.08762
439.06168
424.09140
393.15918
403.11258
415.10367
430.21008
m/z

D23

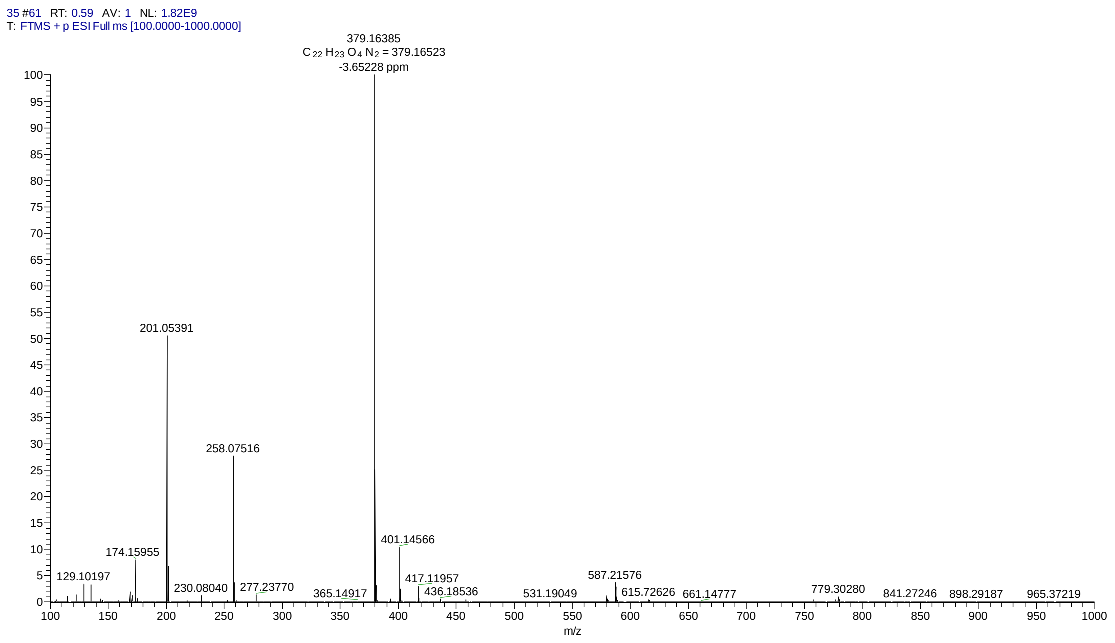
35 #61 RT: 0.59 AV: 1 NL: 1.82E9
T: FTMS + p ESI Full ms [100.0000-1000.0000]
379.16385
C 22 H23 O4 N2 = 379.16523
-3.65228 ppm
201.05391
258.07516
174.15955
129.10197
230.08040
277.23770
365.14917
401.14566
417.11957
436.18536
531.19049
587.21576
615.72626
661.14777
779.30280
841.27246
898.29187
965.37219
m/z

D24

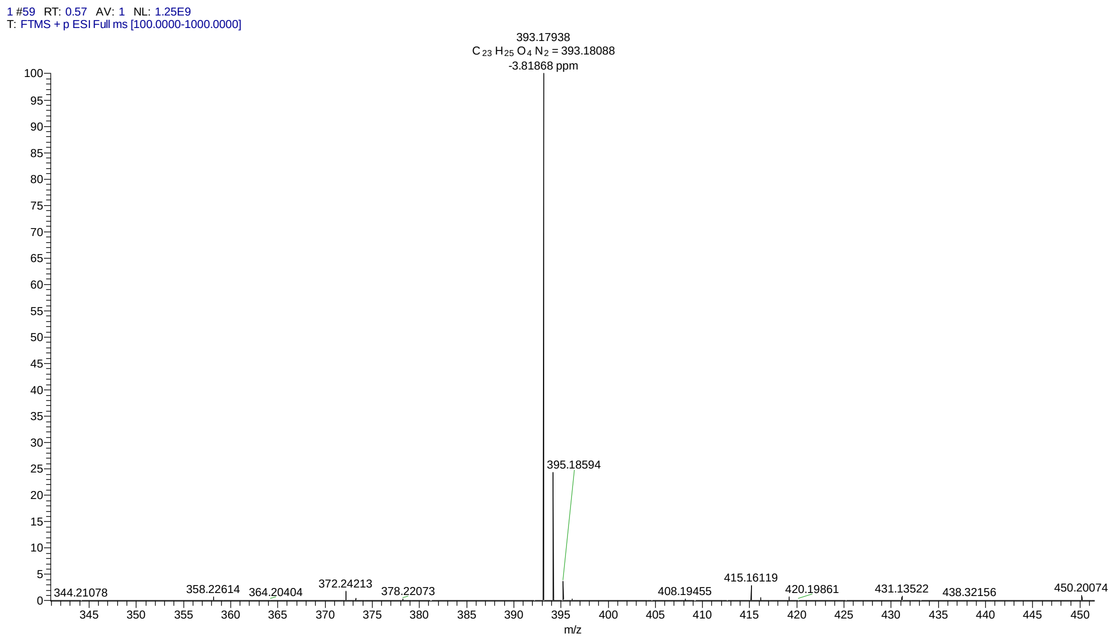
1 #59 RT: 0.57 AV: 1 NL: 1.25E9
T: FTMS + p ESI Full ms [100.0000-1000.0000]
393.17938
C 23 H25 O4 N2 = 393.18088
-3.81868 ppm
395.18594
344.21078
358.22614
364.20404
372.24213
378.22073
408.19455
415.16119
420.19861
431.13522
438.32156
450.20074
m/z

D25

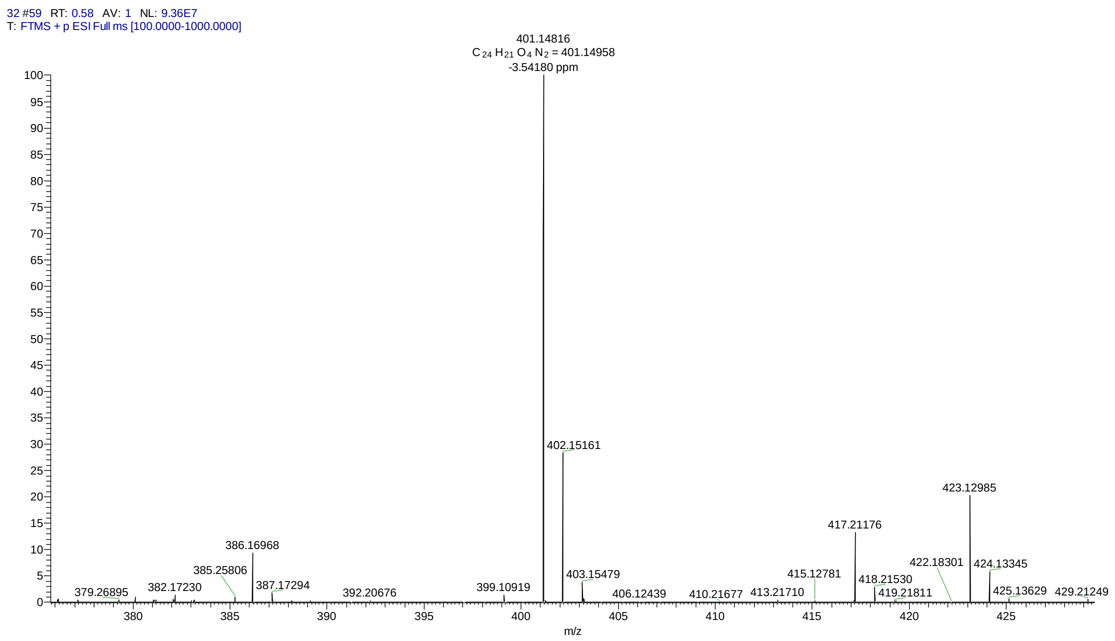
32 #59 RT: 0.58 AV: 1 NL: 9.36E7
T: FTMS + p ESI Full ms [100.0000-1000.0000]
401.14816
C 24 H 21 O 4 N 2 = 401.14958
-3.54180 ppm
402.15161
423.12985
417.21176
386.16968
385.25806
387.17294
379.26895
382.17230
392.20676
399.10919
403.15479
406.12439
410.21677
413.21710
415.12781
418.21530
419.21811
422.18301
424.13345
425.13629
429.21249
m/z

D26

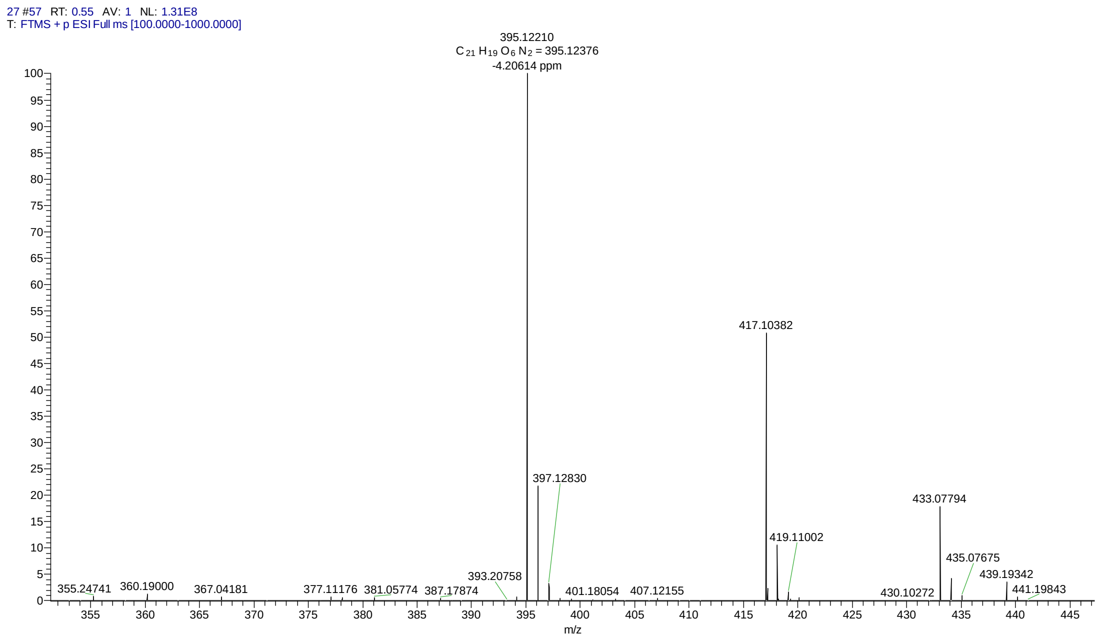
27 #57 RT: 0.55 AV: 1 NL: 1.31E8
T: FTMS + p ESI Full ms [100.0000-1000.0000]
395.12210
C 21 H 19 O 6 N 2 = 395.12376
-4.20614 ppm
417.10382
397.12830
433.07794
419.11002
435.07675
439.19342
393.20758
355.24741
360.19000
367.04181
377.11176
381.05774
387.17874
401.18054
407.12155
430.10272
441.19843
m/z

D27

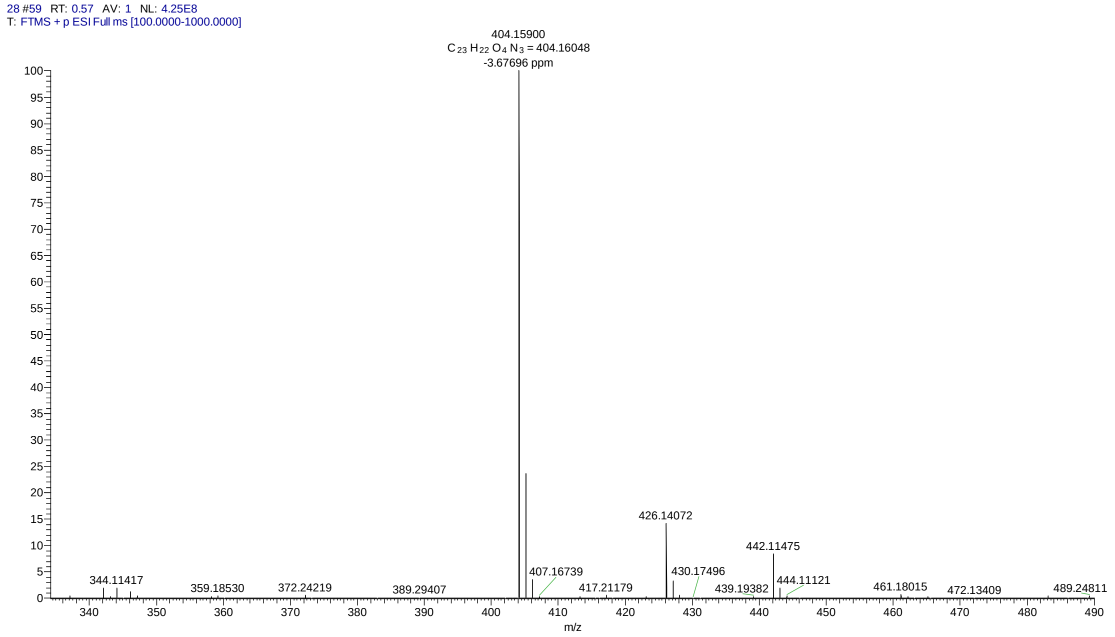
28 #59 RT: 0.57 AV: 1 NL: 4.25E8
T: FTMS + p ESI Full ms [100.0000-1000.0000]
404.15900
$C_{23}H_{22}O_4N_3$ = 404.16048
-3.67696 ppm
426.14072
442.11475
344.11417
359.18530
372.24219
389.29407
407.16739
417.21179
430.17496
439.19382
444.11121
461.18015
472.13409
489.24811
m/z

D_{28}

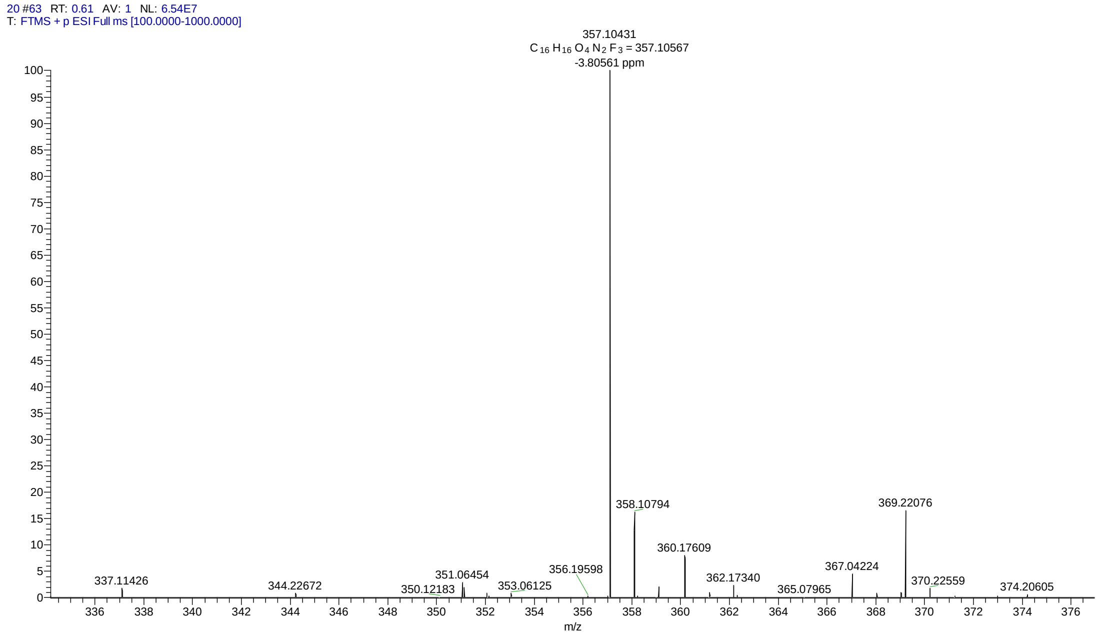
20 #63 RT: 0.61 AV: 1 NL: 6.54E7
T: FTMS + p ESI Full ms [100.0000-1000.0000]
357.10431
$C_{16}H_{16}O_4N_2F_3$ = 357.10567
-3.80561 ppm
358.10794
369.22076
360.17609
337.11426
344.22672
350.12183
351.06454
353.06125
356.19598
362.17340
365.07965
367.04224
370.22559
374.20605
m/z

J_1

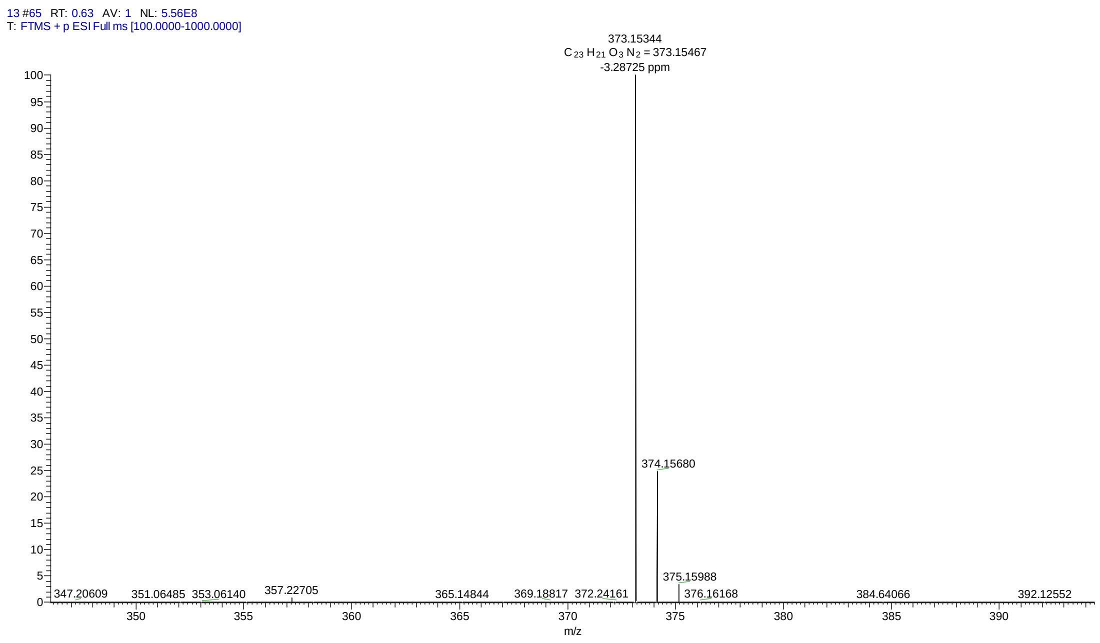
13 #65 RT: 0.63 AV: 1 NL: 5.56E8
T: FTMS + p ESI Full ms [100.0000-1000.0000]
373.15344
C 23 H 21 O 3 N 2 = 373.15467
-3.28725 ppm
374.15680
375.15988
376.16168
347.20609
351.06485
353.06140
357.22705
365.14844
369.18817
372.24161
384.64066
392.12552
m/z
